# Whole-transcriptome sequencing in neural and non-neural tissues of a mouse model identifies miR-34a as a key regulator in SMA pathogenesis

**DOI:** 10.1016/j.omtn.2025.102490

**Published:** 2025-02-20

**Authors:** Liucheng Wu, Junjie Sun, Li Wang, Zhiheng Chen, Zeyuan Guan, Lili Du, Ruobing Qu, Chun Liu, Yixiang Shao, Yimin Hua

**Affiliations:** 1 Department of Neurology and Suzhou Clinical Research Center of Neurological Disease, The Second Affiliated Hospital of Soochow University, Suzhou 215004, China; 2 Laboratory Animal Center, Nantong University, Nantong 226001, China; 3 Institute of Neuroscience, Soochow University, 199 Renai Road, Suzhou, Jiangsu 215123, China; 4 Key Laboratory of Neuroregeneration of Jiangsu and Ministry of Education, Co-Innovation Center of Neuroregeneration, Nantong University, Nantong 226001, China; 5 Jiangsu Key Laboratory for Molecular and Medical Biotechnology, College of Life Sciences, Nanjing Normal University, Nanjing 210023, China; 6 Laboratory Animal Center, Nanjing University of Chinese Medicine, Nanjing, Jiangsu 210023, China

**Keywords:** MT: Non-coding RNAs, spinal muscular atrophy, SMN, miR-34a, ceRNA network, cell cycle, *Spag5*

## Abstract

Spinal muscular atrophy (SMA) is a severe neurodegenerative disorder caused by deficiency of survival of motor neuron (SMN). While significant progress has been made in SMA therapy by rescuing SMN expression, limited knowledge about SMN downstream genes has hindered the development of alternative therapies. Here, we conducted whole-transcriptome sequencing of spinal cord, heart, and liver tissues of a severe SMA mouse model at early postnatal ages to explore critical coding and non-coding RNAs (ncRNAs). A large number of differentially expressed RNAs (DE-RNAs) were obtained, including 2,771 mRNAs, 382 microRNAs (miRNAs), 1,633 long ncRNAs, and 1,519 circular RNAs. Through in-depth data mining, we unveiled deregulation of miR-34a in all tissues. Analysis of competitive endogenous RNA networks of DE-RNAs identified multiple novel targets of miR-34a including *Spag5* mRNA, lncRNA00138536, and circRNA007386. Further *in vitro* studies using mouse myoblast and human cardiomyocyte cell lines showed that knockdown of SMN upregulated miR-34a-5p and overexpression of miR-34a-5p alone disrupted cell-cycle progression through regulating its targets, recapitulating gene expression patterns observed in cardiac tissue of SMA mice. Our results identified a critical miRNA involved in SMA pathology, which sheds insights into the molecular basis of widespread tissue abnormalities observed in severe forms of SMA.

## Graphical abstract

**Figure:**
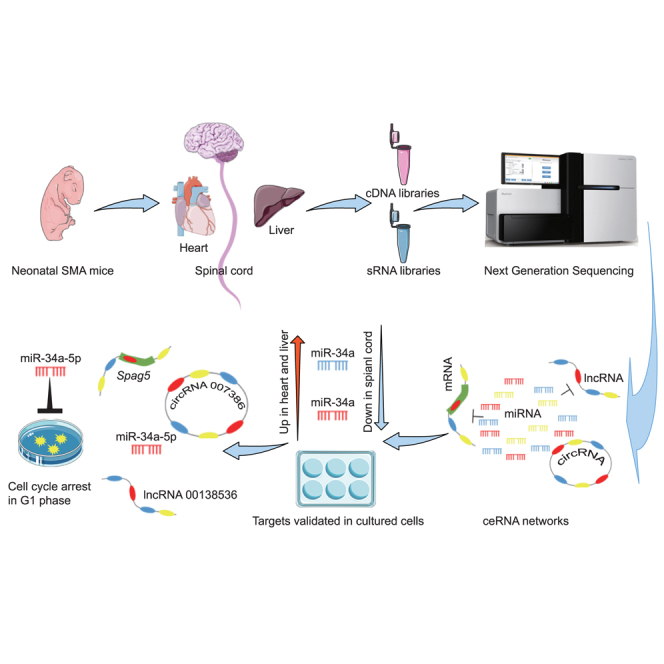

## Teaser

Whole transcriptome RNA-seq was performed to explore both coding and ncRNAs deregulated in neural and non-neural tissues derived from an SMA mouse model, and a large number of demRNAs/ncRNAs were uncovered. ceRNET analysis identified miR-34a and its novel targets that halt cell cycle progression of cardiomyocytes.

## Introduction

Spinal muscular atrophy (SMA) is a devastating genetic disease characterized by degeneration of α-motor neurons in the anterior horn of the spinal cord, leading to progressive skeletal muscle weakness and atrophy. It is caused by mutations in the *survival of motor neuron 1* (*SMN1*) gene. The incidence of SMA is 1/6-000–1/10,000 in newborns with a carrier rate of 1/40–1/60. According to the age of onset and the severity of the disease, it is divided into three main types and two less common types, with type 0 being the most severe form and type IV being the mildest adult-onset one.^1^ Humans have a closely related paralogous gene called *SMN2* and the two genes encode an identical SMN protein. However, owing to two single-nucleotide substitutions relative to *SMN1*, C6T in exon 7, and to a lesser extent G-44A in intron 6,^2^^,^^3^ *SMN2* exon 7 is predominantly skipped during pre-mRNA splicing and the truncated protein isoform is unstable and dysfunctional. *SMN2* produces only approximately 10% of the full-length functional SMN, which is not sufficient to compensate for the loss of function of *SMN1*.

SMN is a house-keeping protein that is ubiquitously expressed and localized in both the cytoplasm and nucleus. In the nucleus, it often concentrates into membraneless organelles called gems and Cajal bodies.^4^^,^^5^^,^^6^ SMN self-oligomerizes and interacts with a large number of proteins through its distinct domains including the basic/lysine-rich region, Tudor domain, proline-rich region, and YG-box. SMN is the central component of a tight macromolecular complex including GEMIN2-8 and Unrip.^7^ The major function of SMN is to facilitate the biogenesis of various ribonucleoproteins (RNPs), especially the spliceosomal U-rich small nuclear RNPs.^8^ SMN has also been implicated in mRNP intracellular trafficking in neurites,^9^^,^^10^ R-loop resolution in transcription termination,^11^ and protein translation of a subset of mRNAs.^12^ The past three decades have witnessed great advances in understanding the functions of SMN, as well as regulation of *SMN2* splicing, and the latter leads to development of two splicing-modulating drugs to treat the disease.^7^^,^^13^^,^^14^ However, the exact molecular mechanism downstream of SMN deficiency that causes motor neuron death remains a mystery. Observations of widespread defects in non-neural tissues including heart, liver, pancreas, intestine, and lung in severe forms in human patients, and more prominently in animal models,^15^ not only establish SMA as a multisystem disease but also further complicates understanding of the pathogenesis of the disease.

Gene expression is regulated at multiple levels. The tremendous advances in high-throughput sequencing technology allow us to analyze the complex network of interactions among different types of RNA within the cell. Competing endogenous RNAs (ceRNAs) are a group of RNAs that harbor similar if not identical microRNA (miRNA)-responsive elements (MREs) for common miRNAs and thus regulate each other through competitive binding to miRNAs.^16^ Major components of ceRNAs are various non-coding RNAs (ncRNAs), including pseudogene RNAs, long ncRNAs (lncRNAs), and circular RNAs (circRNAs), which can serve as miRNA sponges to regulate mRNA abundance. As post-transcriptional regulators, ceRNAs are involved in various biological processes and play an important role in development and disease.^17^ Particularly, ceRNA crosstalk has been found involved in a broad spectrum of neurodegenerative diseases. For example, Tan et al.^18^ revealed that mutations in *ATXN7* disrupt miR-124-mediated crosstalk between a conserved lncRNA (lnc-SCA7) and the *ATXN7* mRNA, providing an explanation why mutations in a house-keeping gene cause tissue-specific defects in the retina and cerebellum. Another typical ceRNA example is the *GBA* pseudogene *GBAP1*. *GBA* encodes glucocerebrosidase, deficiency of which is implicated in several medical conditions, such as Gaucher’s disease, Parkinson’s disease, dementia with Lewy bodies, and REM sleep behavior disorders. Straniero et al. unveiled that miR-22-3p binds to the 3′ UTR) of both *GBA* and *GBAP1* transcripts, and *GBAP1* 3′ UTR over-expression upregulates *GBA*, suggesting a promising approach for targeted drug development.^19^

Prior studies using SMA mouse tissues and/or cultured cells including patients’ cells have demonstrated that SMN deficiency causes deregulation of multiple miRNAs.^20^^,^^21^^,^^22^ Some of them have been considered as potential circulating biomarkers in SMA.^20^^,^^23^ To our knowledge, aside from these miRNAs, no other deregulated ncRNA types associated with SMA have been reported. d’Ydewalle et al.^24^ uncovered that a neuronally enriched antisense transcript is transcribed from the antisense strand at the *SMN1/2* loci, which represses expression of the sense transcripts by recruiting the Polycomb repressive complex 2 (PRC2) to their loci. Indeed, antisense oligonucleotides that disrupt the interaction between the antisense RNA and PRC2 increase SMN levels in cultured primary neurons.^25^ The *SMN1/2* loci also produce a vast repertoire of circRNAs and some of them mildly affect expression of their linear counterparts.^26^^,^^27^ Overall, comprehensive studies on ncRNAs and their roles in SMA pathogenesis are lacking.

Analysis of ceRNA networks (ceRNETs) in SMA mouse tissues has potential to identify critical nodes or genes in signaling pathways that are responsible or contribute to SMA. In this study, we performed a whole transcriptome RNA sequencing (RNA-seq) study to explore both coding and ncRNAs deregulated in spinal cord, heart, and liver tissues derived from a severe SMA mouse model, and uncovered a large number of differentially expressed lncRNAs, miRNAs, circRNAs, and mRNAs. Among them, miR-34a is deregulated in all three tissues. We further constructed miR-34a ceRNETs and found that multiple deregulated coding and non-coding genes involved in cell-cycle regulation are novel targets of miR-34a-5p. *In vitro* studies using siRNA knockdown of SMN and an oligonucleotide that mimics miR-34a-5p in both mouse and human cell lines recapitulated the gene expression pattern in the heart of SMA mice. Our data not only revealed a critical miRNA in SMA pathogenesis but also provided a resource for further studies on the molecular mechanisms of phenotypes present in neural and non-neural tissues.

## Results

### RNA-seq differential expression analysis in a severe SMA mouse model

**Figure 1:**
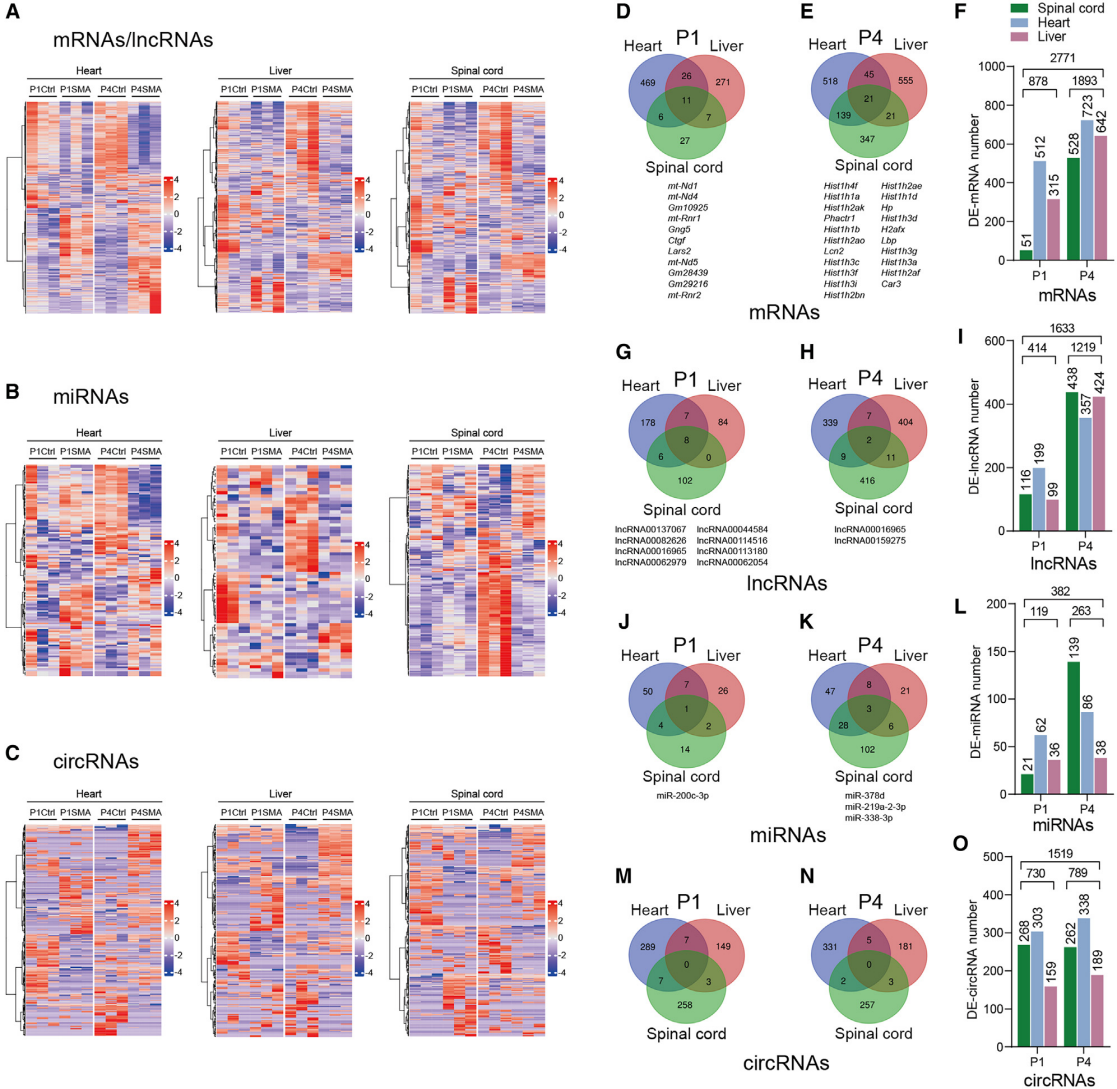
Transcriptome RNA-seq analysis of DE-mRNAs and DE-ncRNAs in the spinal cord, heart, and liver of SMA mice (A–C) Heatmaps displaying DE-mRNAs/DE-lncRNAs, DE-miRNAs, and DE-circRNAs in spinal cord, heart, and liver samples collected at P1 and P4. Each column represents a sample and each row represents an RNA; red represents upregulated DEGs and blue represents downregulated DEGs; the darker the color, the greater the magnitude of change. RNA expression values of SMA mouse tissues are presented as alog2 ratio compared with heterozygous mice (Ctrl). (D–O) Venn diagrams and histograms displaying the numbers of the four types of DE-RNAs in different tissues and two time points; DE-RNAs overlapping in all three tissues are listed below.

A Taiwanese mouse model with a lifespan of approximately 10–11 days has been widely used for pathogenesis study and drug development of SMA.^13^^,^^28^^,^^29^^,^^30^ Previous studies reported structural and functional pathologies in both CNS and non-neural tissues in the severe model.^13^^,^^31^^,^^32^^,^^33^^,^^34^^,^^35^ Our recent histological examinations of neonatal tissues of the same model confirmed histological abnormalities in the spinal cord, liver, and heart (Figure S1). These phenotypical features manifested in the early symptomatic stages prompted us to investigate deregulated RNAs in the three tissues using the whole-transcriptome RNA-seq method, attempting to identify early gene alterations and defective cellular events shared by multiple affected tissues. Tissues were collected at postnatal day 1 (P1) and P4 with heterozygous mice from the same litter being used as controls and 36 total RNA samples were extracted (Figures S2A and S2B). We constructed a total of 72 RNA-seq libraries including 36 cDNA libraries for small RNAs (sRNAs), and obtained 16,627 and 17,390 genes in P1 and P4 spinal cord samples, respectively, 15,757 and 15,677 genes in P1 and P4 heart samples, respectively, and 15,528 and 16,506 genes in P1 and P4 liver samples. Both expression correlation and principal component analyses indicate good repeatability between the samples (Figures S2C–S2F). For mRNAs, only those with a q value of less than 0.05 and fold changes of more than 2 were considered differentially expressed; for ncRNAs, the threshold is a *p* value of less than 0.05 and fold changes of more than 2. We identified a total of 2,771 differentially expressed mRNAs (DE-mRNAs): 878 detected in P1 samples and 1,893 in P4 samples (Figure 1A). Among them, 579 DE-mRNAs were detected in spinal cord with 51 in P1 and 528 in P4, 1235 in heart with 357 in P1 and 878 in P4, and 957 in liver with 315 in P1 and 642 in P4 (Figures 1D–1F). On the other hand, 3,534 ncRNAs exhibited expression changes, including 1,633 DE-lncRNAs, 382 DE-miRNAs, and 1,519 DE-circRNAs (Figures 1A–1O). Among the 16,33 DE-lncRNAs, 554 were detected in spinal cord with 116 in P1 and 438 in P4, 556 in heart with 199 in P1 and 357 in P4, and 523 in liver with 99 in P1 and 424 in P4 (Figure 1I). Among the 382 DE-miRNAs, 160 were detected in spinal cord with 21 in P1 and 139 in P4, 148 in heart with 65 in P1 and 86 in P4, and 74 in liver with 36 in P1 and 38 in P4 (Figure 1L). As for the 1519 DE-circRNAs, 530 were identified in spinal cord with 268 in P1 and 262 in P4, 641 in heart with 303 in P1 and 338 in P4, and 348 in liver with 159 in P1 and 189 in P4 (Figure 1O).

Overall, more differentially expressed genes (DEGs) were observed in non-neural tissues, particularly in heart, than in spinal cord, which is more evident at the age of P1 than P4.

### Functional enrichment analysis of DE-mRNAs

**Figure 2:**
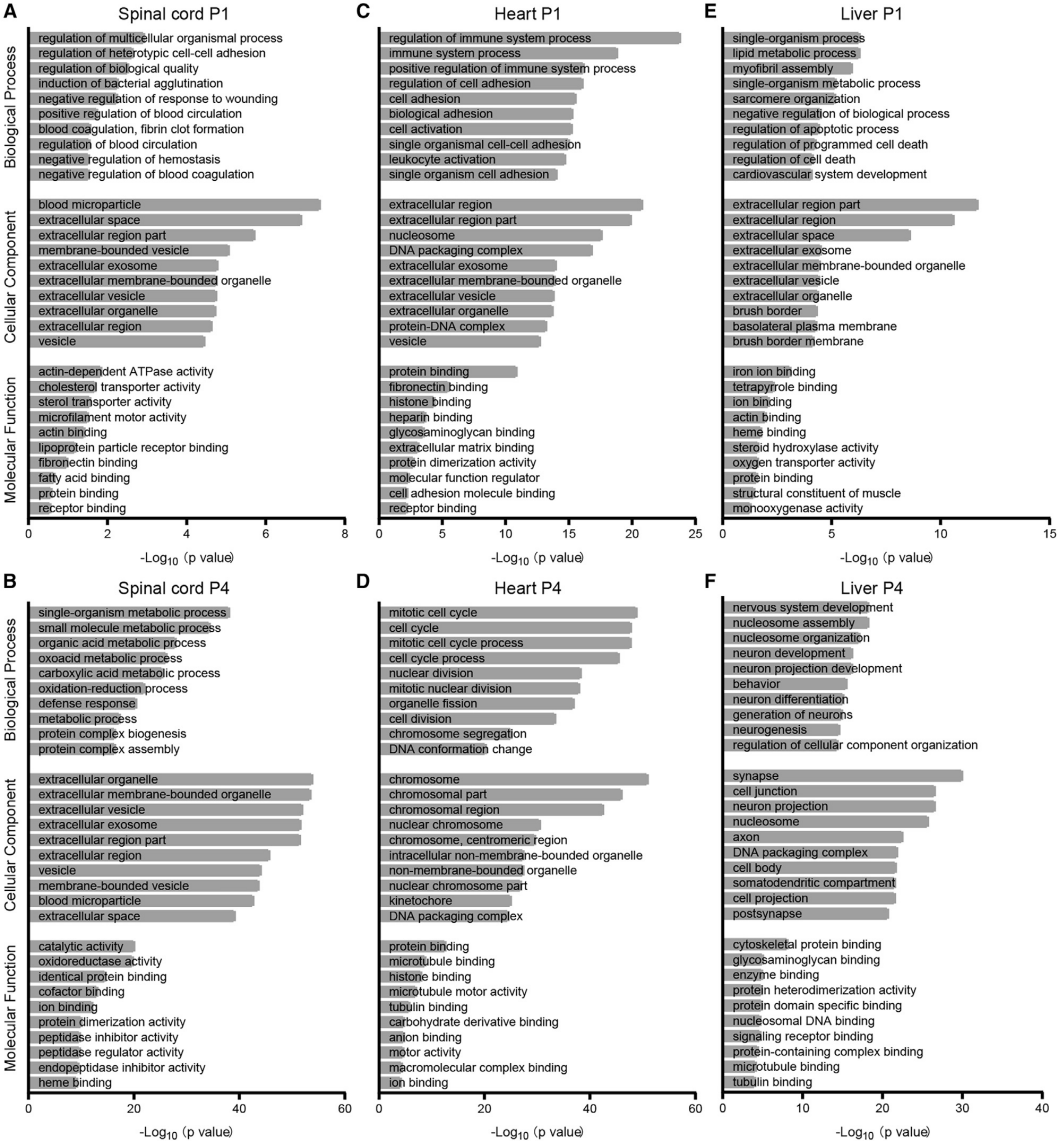
GO term enrichment analysis of DE-mRNAs in the spinal cord, heart, and liver of SMA mice GO term analysis revealed top 10 enriched GO-BP, -CC, and -MF terms for DE-mRNAs of P1 spinal cord (A), P4 spinal cord (B), P1 heart (C), P4 heart (D), P1 liver (E), and P4 liver (F) tissues of SMA mice compared with heterozygous mice. The enrichment score was calculated as −log_10_ (*p* value).

To explore the potential roles of these detected DE-mRNAs in SMA pathogenesis, we performed a functional enrichment analysis of Gene Ontology (GO) terms using the GO Knowledgebase webserver.^36^ Figure 2 presents the top 10 GO biological process (GO-BP), cellular component (GO-CC), and molecular function (GO-MF) terms enriched for the DE-mRNA genes. Enriched GO-CC terms in spinal cord have much lower *p* values compared with GO-BP and GO-MF terms, and multiple terms related to the extracellular space were enriched at both time points, which is more pronounced at P4 than P1, suggesting that processes occurring in the extracellular space, such as cell adhesion or cell-to-cell communication, may be affected by a lack of SMN (Figures 2A and 2B). GO-BP terms related to metabolic processes were also enriched in spinal cord at P4 but not at P1. For heart DE-mRNAs, the most enriched GO-BP terms are related to immune system regulation and cell adhesion (P1), as well as mitotic cell cycle (P4), while most GO-CC terms are highly associated with extracellular region (P1) and chromosome (P4) (Figures 2C and 2D). For liver DE-mRNAs, the top GO-BP terms are metabolic processing and cell death (P1), as well as neuron development and nucleosome/chromosome assembly (P4); the most enriched GO-CC terms are related to the extracellular region (P1), similar to in P1 heart, and neuronal development and function (P4), which is unexpected (Figures 2E and 2F). The *p* values of spinal cord DE-mRNAs annotated in GO-BP terms are generally low compared with those of heart and liver tissues. The *p* values of DE-mRNAs in GO-MF analysis were much smaller in all groups compared with the other two GO categories. The three tissues share a large number of DE-mRNAs associated with binding such as protein binding and ion binding, although each tissue has their own specific GO-MF terms.

### Identification of miR-34a as a widespread deregulated miRNA in all tissues

**Figure 3:**
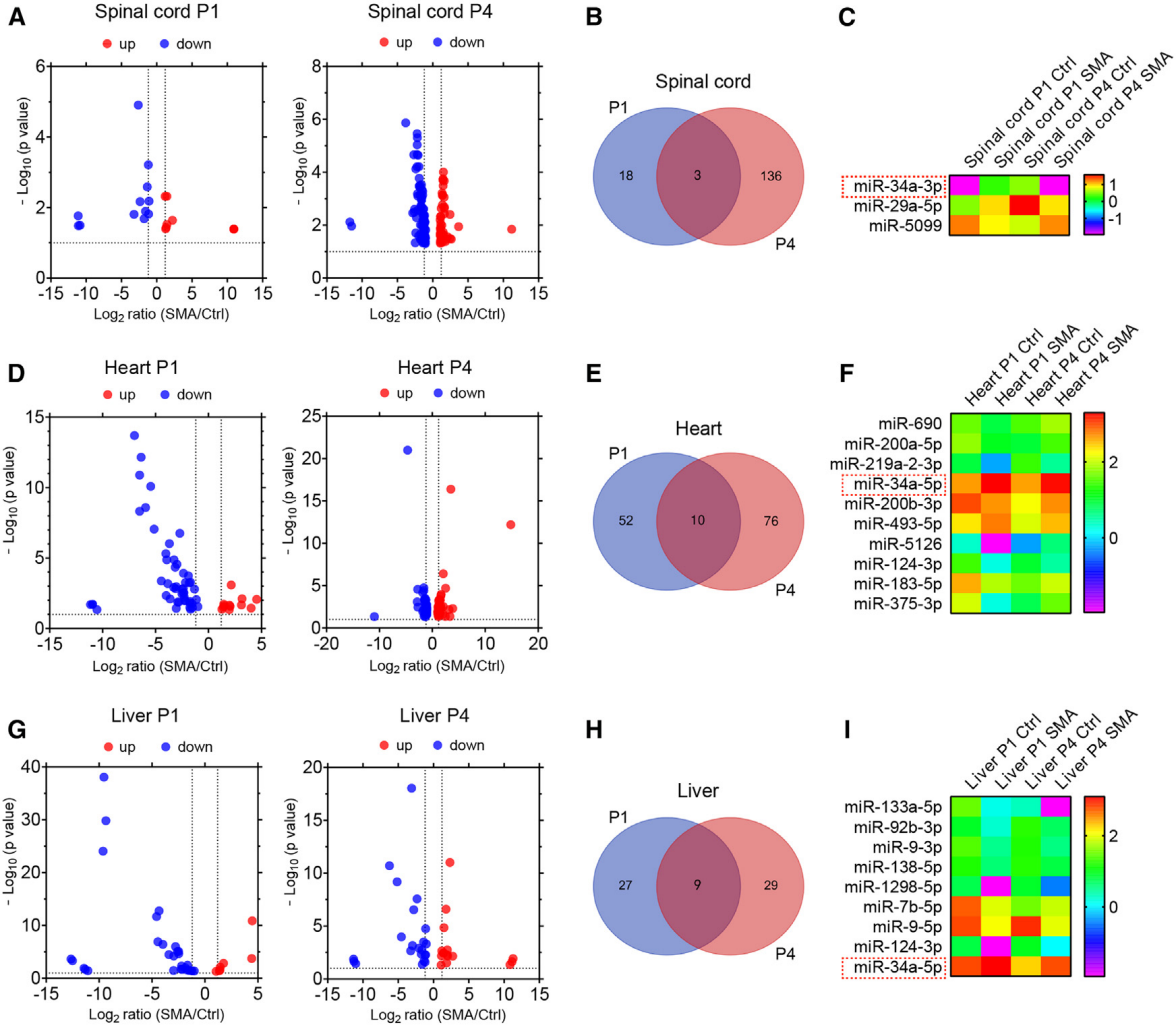
A plethora of miRNAs deregulated in SMA mouse tissues Volcano plots showing all DE-miRNAs of the RNA-seq data and Venn diagrams and heatmaps showing miRNAs deregulated at both P1 and P4 in the spinal cord (A–C), heart (D–F), and liver (G–I) of SMA mice.

Considering the key role of miRNAs in ceRNET interactions, we next sought to identify key DE-miRNAs in the three tissues. Spinal cord samples at both time points had 3 DE-miRNAs in common: miR-34a-3p, miR-29a-5p, and miR-5099; heart P1 and P4 samples shared 10 DE-miRNAs: miR-124-3p, miR-34a-5p, miR-493-5p, miR-690, miR-200a-5p, miR-200b-3p, miR-219a-2-3p, miR-5126, miR-183-5p, and miR-375-3p; and liver P1 and P4 samples shared 8 miRNAs: miR-133a-5p, miR-92b-3p, miR-9-3p, miR-138-5p, miR-1298-5p, miR-7b-5p, miR-9-5p, miR-124-3p, and miR-34a-5p (Figures 3A–3I). One miRNA, miR-34a, is present in all the shared DE-miRNAs in the three tissues. Interestingly, we detected upregulation of miR-34a-3p but not its guide strand in P1 spinal cord and it became downregulated at P4. On the other hand, miR-34a-5p but not its passenger strand was upregulated in two non-neural tissues, and it was consistent between the two time points. We validated miR-34a-5p expression in multiple tissues using real-time quantitative RT-PCR (RT-qPCR) and found that it was upregulated more or less in all examined tissues compared with heterozygous mice; among them, heart and spleen were the two most upregulated tissues both with a more than 5-fold increase (Figure S3).

**Figure 4:**
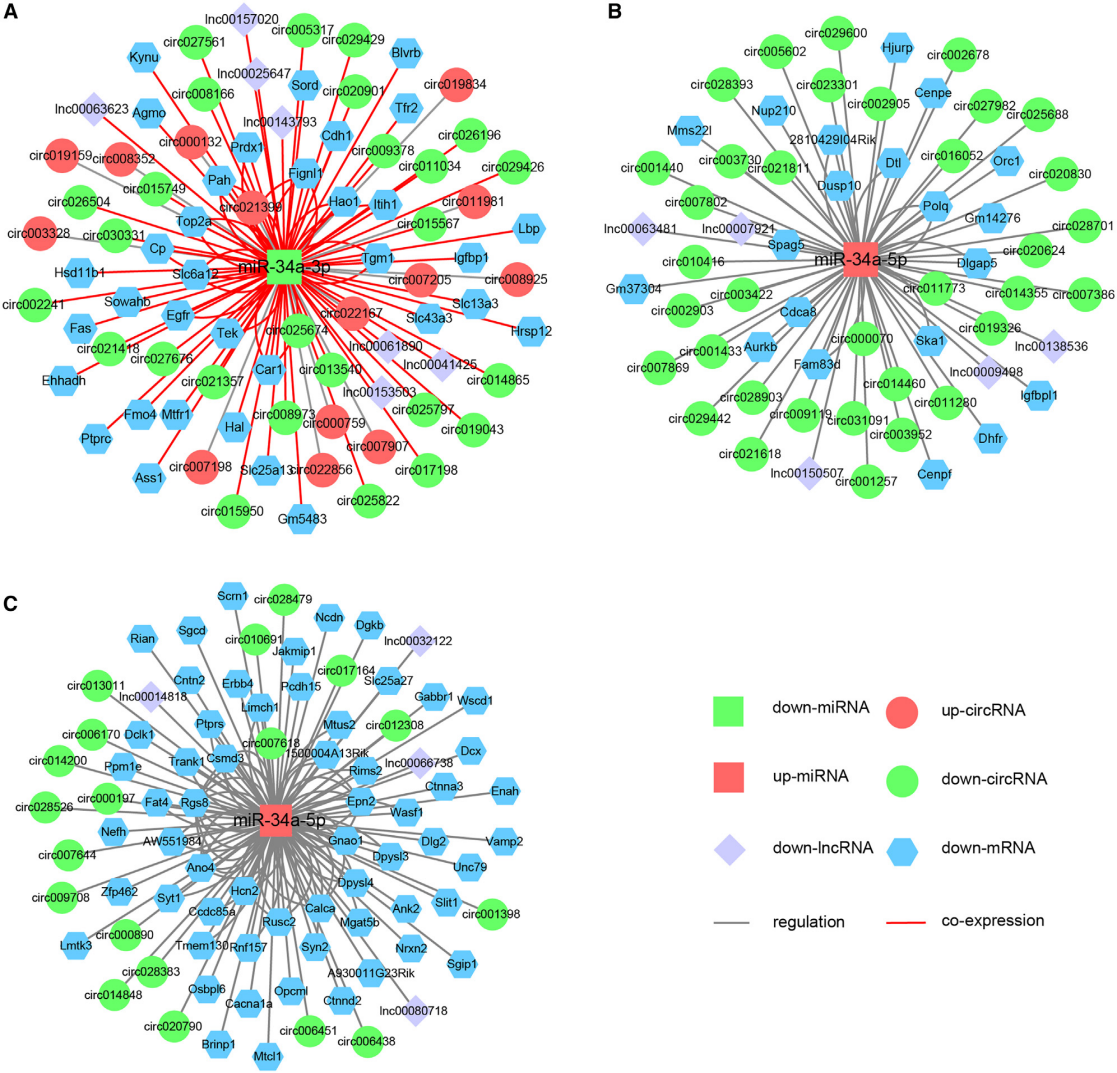
Constructed miR-34a ceRNETs with DEGs in the spinal cord, heart, and liver of SMA mice (A) The differentially expressed lncRNA-circRNA-mRNA network regulated by miR-34a-3p in the P4 spinal cord samples was constructed, including 53 mRNAs, 7 lncRNAs, and 40 circRNAs. (B and C) The DEG networks regulated by miR-34a-5p were constructed for P4 heart samples including 20 mRNAs, 5 lncRNAs, and 36 circRNAs, and for P4 liver samples including 47 mRNAs, 4 lncRNAs, and 20 circRNAs.

We subsequently constructed six ceRNETs of detected DEGs in the three tissues. Targets of miRNAs were predicted by databases TargetScan^37^ and miRanda.^38^ Using Cytoscape,^39^ we acquired significant network modules in the three tissues (Figures S4–S9 and Table S1). The proportion of DE-mRNAs in ceRNETs is the largest, followed by circRNAs, lncRNAs, and miRNAs (Figure S10). The P1 spinal cord ceRNET contains only 160 DEGs while P1 heart and liver ceRNETs contains 958 and 305 DEGs, respectively (Figures S10A–S10F). At P4, the DEG numbers in the spinal cord, heart, and liver ceRNETs are 1,303, 1,689, and 1,333, respectively, which are approximately 10-, 2-, and 4-fold of the number of each corresponding P1 network (Figures S10G–S10L). We looked at miR-34a regulatory networks with DEGs detected at P4. In spinal cord, downregulated miR-34a-3p likely acts as an important node since it displayed 100 interactions, including interactions with 40 DE-circRNAs, 7 DE-lncRNAs, and 53 DE-mRNAs (Figure 4A and Table S2). In heart, 60 DEGs are linked to miR-34a-5p, including 35 DE-circRNAs, 5 DE-lncRNAs, and 20 DE-mRNAs (Figure 4B and Table S2); in liver, 131 DEGs linked to miR-34a-5p includes 29 DE-circRNAs, 4 DE-lncRNAs, and 112 DE-mRNAs (Figure 4C and Table S2). Notably, the ceRNET of miR-34a-5p in P4 heart differs substantially from that in P4 liver, reflecting tissue- and context-specific regulatory roles of the miRNA.

### Experimental validation of miR-34a-5p and its target ceRNAs in cardiac tissue of SMA mice

**Figure 5:**
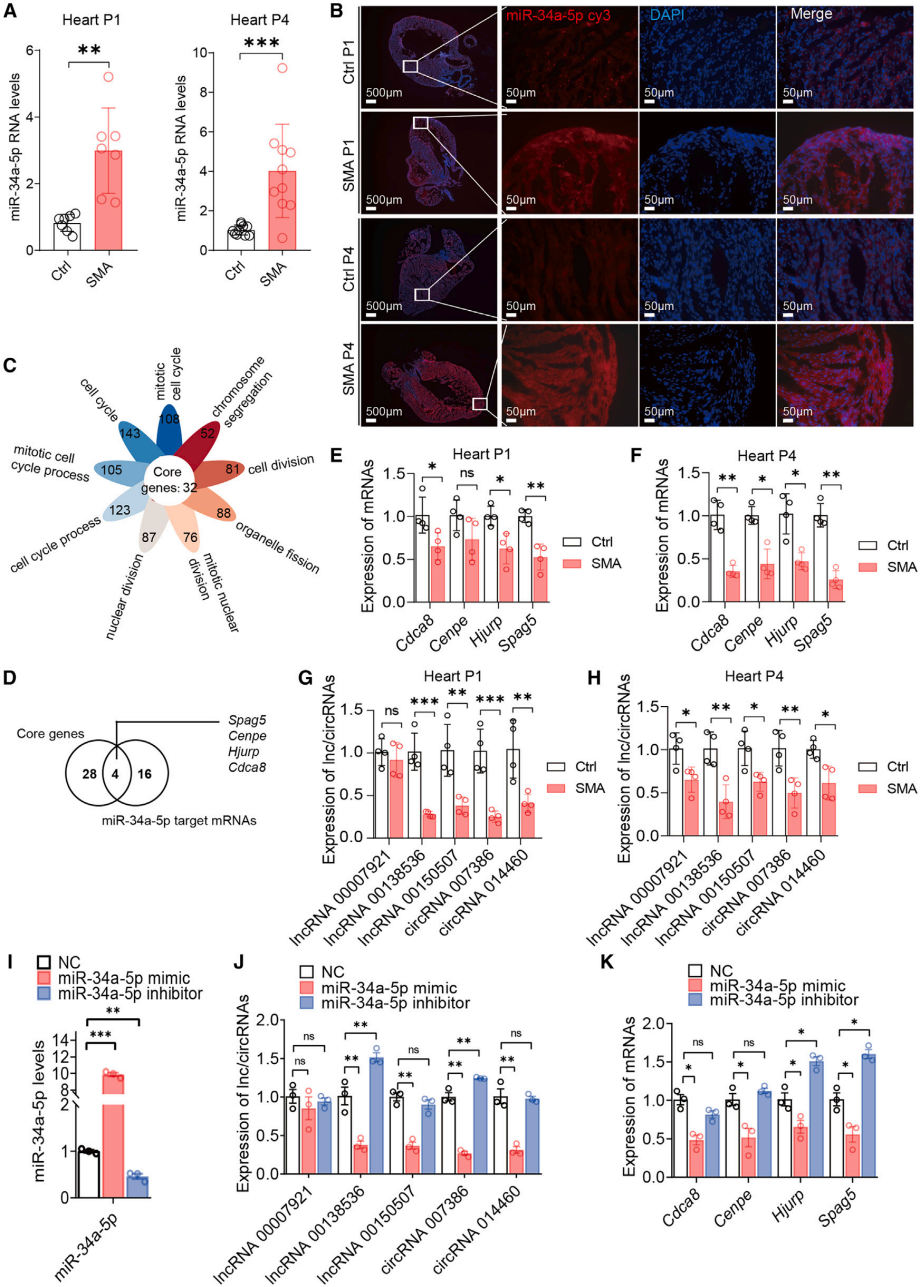
Validation of expression changes of miR-34a-5p and its targets in the heart of SMA mice and C2C12 cells (A) Histograms of RT-qPCR analysis showing robust upregulation of miR-34a-5p in both P1 (*n* = 7) and P4 (*n* = 10) heart samples. (B) FISH detected strong signals of miR-34a-5p in P1 and P4 SMA heart samples compared with heterozygous mice (Ctrl, *n* = 3) using a Cy3-labelled probe (red). DAPI was used to stain the nucleus (blue). Scale bar, 500 or 50 μm as indicated. (C) Venn diagrams of DE-mRNAs detected in P4 heart samples (see Figure 2D) showing 32 core genes involved in all cell-cycle-related processes. (D) Venn diagram showing four genes overlapping between the 32 core genes and 20 DE-mRNAs that are predicted as targets of miR-34a-5p. (E–H) Histograms showing RT-qPCR analysis of nine targets of miR-34a-5p including four mRNAs (*n* = 4), three lncRNAs, and two circRNAs, as indicated. (I) Detection of miR-34a-5p levels in C2C12 cells after treatment with 50 nM of miR-mimic, miR-inhibitor, or NC-oligo (NC) (*n* = 3). (J and K) Expression changes of the target mRNAs, lncRNAs, and circRNAs in treated C2C12 cells as in (I). ∗*p* < 0.05, ∗∗*p* < 0.01, ∗∗∗*p* < 0.001.

Studies in past years have established SMA as a multi-organ disease, particularly the severe forms. Cardiovascular abnormalities in SMA patients and animal models have been widely documented.^40^^,^^41^ We previously showed that cardiomyocytes of the severe Taiwan model undergo cell-cycle arrest, which is partly caused by downregulation of the Survivin-encoding gene *Birc5*^32^; however, the underlying mechanism has not been fully understood. Interestingly, multiple studies have reported that miR-34a-5p inhibits Survivin expression.^42^^,^^43^^,^^44^ We performed RT-qPCR with heart samples and observed an approximately 3-fold increase of miR-34a at P1 and approximately 4-fold at P4 (Figure 5A), consistent with the RNA-seq data. Moreover, fluorescence *in situ* hybridization (FISH) assay using a Cy3 RNA probe revealed a much stronger fluorescence signal of the miRNA in the heart sections of SMA mice than in the control group (Figures 5B and S11). These data confirm upregulation of miR-34a in heart tissue of the mouse model.

GO term analysis in P4 cardiac samples revealed that the nine most enriched GO-BP terms are all related to the cell cycle (Figure 2D), and 32 DE-mRNAs were detected in all nine GO-BP categories (Figure 5C). In the predicted ceRNETs, 20 DE-mRNAs are targeted by miR-34a-5p. We eventually identified four genes (*Cdca8*, *Cenpe*, *Hjurp*, and *Spag5*) that are involved in cell-cycle progression and potentially regulated by miR-34a-5p (Figure 5D). Expression of the four genes in the heart of SMA mice were validated by RT-qPCR, and they were all markedly downregulated at both P1 and P4, with *Spag5* being the most downregulated one (Figures 5E and 5F). Both Western blotting and immunofluorescence confirmed marked reduction of SPAG5 protein levels in heart tissue at both P1 and P4 (Figures S12A–S12E), although expression change of HJURP was not observed (Figures S12F–S12I).

We also validated all five DE-lncRNAs and eight selected DE-circRNAs with a *p* value of less than 0.03 for heart samples (Figure 4B). Four of the 13 ncRNAs (lncRNA00138536, lncRNA00150507, circRNA007386, and circRNA014460) were markedly downregulated at both P1 and P4, and one (lncRNA00007921) was downregulated only at P4 (Figures 5G and 5H).

### Validation of effective ceRNAs associated with miR-34a-5p

To identify effective ceRNAs of miR-34a-5p, we took advantage of a mouse myoblast cell line C2C12 and treated cells with a miR-34a-5p oligonucleotide mimic, an oligonucleotide inhibitor, or a non-related oligonucleotide control (hereafter referred to as miR-mimic, miR-inhibitor, and NC-oligo, respectively) to examine expression changes of the above putative ceRNAs using RT-qPCR. As shown in Figures 5I–5K, lncRNA00138536, lncRNA00150507, circRNA007386, circRNA014460, and mRNAs expressed from *Cdca8*, *Cenpe, Hjurp*, and *Spag5* were markedly downregulated in the miR-mimic-treated cells with exception of lncRNA00007921 whose expression was not altered, while lncRNA00138536, circRNA007386, and mRNAs from *Hjurp* and *Spag5* were markedly upregulated in the miR-inhibitor-treated cells with the exception of the *Cdca8* and *Cenpe* mRNAs being non-altered. Two ncRNAs (lncRNA00138536 and circRNA007386) responded to both treatments. Upregulation of the two ncRNAs in heart samples of SMA mice was also confirmed by RT-qPCR (Figure S13). Moreover, we conducted western blotting using the above treated cells and found that SPAG5 protein levels were markedly decreased after miR-mimic treatment but moderately increased after miR-inhibitor treatment (Figure S14). Taken together, these data demonstrate that at least lncRNA00138536, circRNA007386, and two mRNAs (*Spag5* and *Hjurp*) are authentic targets of miR-34a-5p.

**Figure 6:**
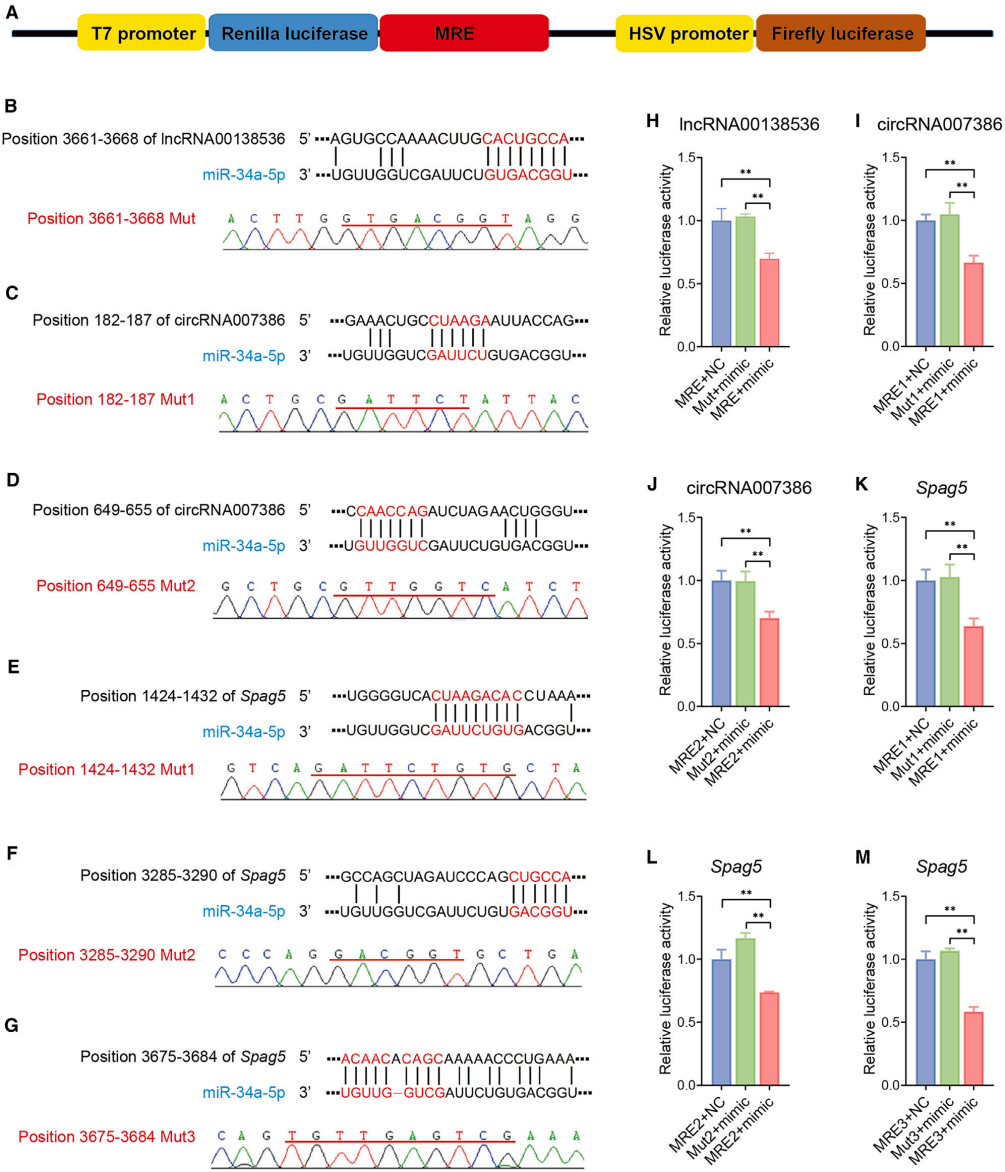
Dual luciferase assay verified miR-34a-5p MREs in three target genes in HEK293T cells (A) Schematic diagram of the dual luciferase reporter construct. (B–G) Predicted miR-34a-5p MRE sites in lncRNA00138536, circRNA007386, and the *Spag5* mRNA that are well base-paired with the corresponding mouse miR-34a-5p sequence. Sequencing results of the MRE mutation sites in mutant reporters were shown below. (H–M) Dual luciferase analysis in HEK293T cells co-transfected with 1 μg of a WT or mutant reporter plasmid and 50 nM miR-mimic or NC-oligo. MRE mutation (Mut) in lncRNA00138536 is shown in (B); mutations in circRNA007386 (Mut1 and Mut2) are shown in (C) and (D), respectively; mutations in the *Spag5* mRNA (Mut 1, Mut2, and Mut3) are shown in (E), (F), and (G), respectively. Expression levels are expressed as relative luciferase activity (firefly/Renilla) with the NC-oligo control being normalized to 1. For all samples, *n* = 3, ∗∗*p* < 0.01.

We also employed a dual luciferase assay to examine the direct interaction between miR-34a-5p and the three ceRNAs (Figure 6A). The predicted MREs of miR-34a-5p are an 8-nt motif CACUGCCA at positions 3,661–3,668 in lncRNA00138536, two motifs (CUAAGA at positions 182–187 and CAACCAG at positions 649–655) in circRNA007386, and three motifs (CUAAGAGAGA at positions 1,424–1,432 and CUGCCA at positions 3,285–32,90, both located in the coding region, and ACAACACAGC at positions 3,675–3,684 in the 3′ UTR region) in *Spag5* (Figures 6B–6G). Each MRE sequence was cloned into a luciferase reporter in the pmiR-REPORT vector. For each MRE, a mutant was generated by replacing each nucleotide with its complementary counterpart. Each plasmid was con-transfected with miR-mimic or NC-oligo into HEK293T cells. In the presence of miR-34a-5p overexpression, the fluorescence signal of all reporters with an MRE was strongly inhibited compared with that with a mutant MRE, demonstrating that MREs in the three ceRNAs (lncRNA00138536, circRNA007386, and *Spag5* mRNA) are direct targets of miR-34a-5p (Figures 6H–6M).

**Figure 7:**
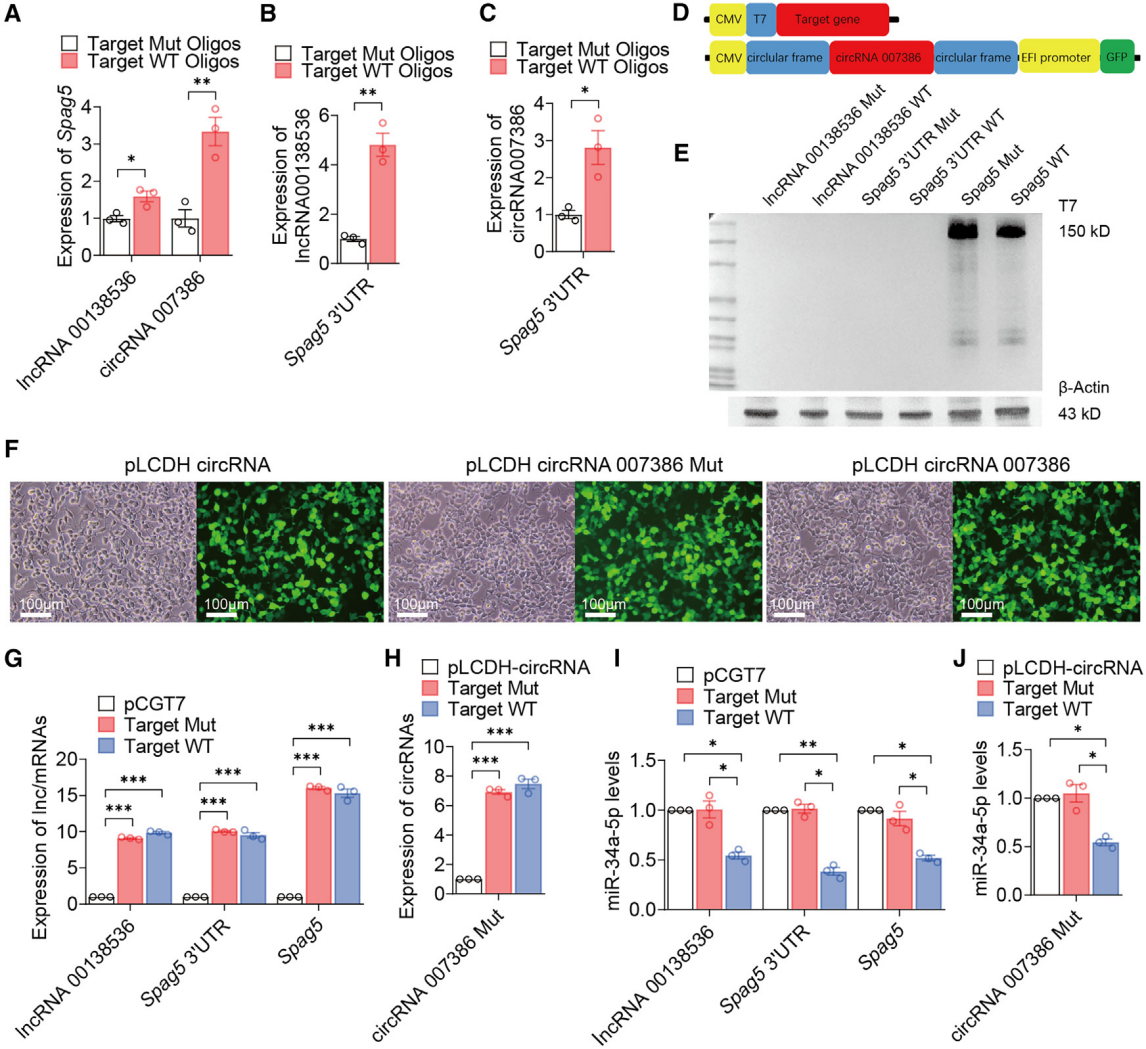
Effects of the putative ceRNAs on the expression of one another and miR-34a in C2C12 cells (A–C) Effect of oligonucleotide decoys on the expression of other miR34a targets. Four oligonucleotides were synthesized with the sequence obtained from the MRE site of lncRNA00138536, circRNA007386n (two sites), and *Spag5* 3′ UTR, respectively; for each oligonucleotide, a control was designed with the MRE being mutated. Both (for the circRNA) or each decoy (for others) was transfected into C2C12 cells and expression of respective transcripts was analyze using RT-qPCR. (D) Schematic diagrams of plasmids expressing transcripts of lncRNA00138536, *Spag5*, *Spag5* 3′ UTR, circRNA007386, and their MRE mutants. (E) Western blot analysis of protein samples from HEK293T cells transfected with 1 μg plasmid expressing each above-mentioned transcript using an anti-T7 antibody. (F) EGFP fluorescence imaging confirming efficient transfection of the empty vector, circRNA007386, and its mutant (scale bar, 100 μm). (G–H) Expression of lncRNA00138536, *Spag5* 3′ UTR, *Spag5* mRNA, circRNA007386 (Target WT), and mutants (Target Mut) expressed from the above-mentioned plasmids was verified by RT-qPCR. (I and J) Downregulation of miR-34a-5p in HEK293T cells expressing either one of the three miR-34a-5p targets compared with respective mutants and empty vectors. For all experiments, *n* = 3; ∗*p* < 0.05, ∗∗*p* < 0.01, ∗∗∗*p* < 0.001 compared with mutants.

To further establish the ceRNET, we synthesized four oligonucleotides: one with a 22-nt sequence obtained from lncRNA00138536 harboring the 8-nt MRE, two with a 21-nt sequence obtained from circRNA007386 harboring one of the two 6-/7-nt MRE motifs, and one with a 23-nt sequence obtained from the *Spag5* 3′ UTR harboring the 10-nt MRE (Table S4). Each oligonucleotide had a matched MRE mutant. We used the oligonucleotides as decoys to capture miR-34a-5p, somehow resembling overexpression of the transcripts from the three target genes. As shown in Figure 7A, *Spag5* mRNA levels were markedly increased in C2C12 cells transfected with decoy oligonucleotides for the two ncRNAs compared with their respective mutants. Similarly, the decoy for the *Spag5* 3′ UTR markedly increased the expression of the two ncRNAs in C2C12 cells (Figures 7B and 7C). These results provide direct evidence of the presence of the ceRNET.

To gain insight into the effects of the ceRNAs on miR34a-5p, we constructed vectors expressing transcripts of *Spag5*, *Spag5* 3′ UTR, lncRNA00138536, circRNA007386, and their MRE mutants, respectively (Figure 7D). Western blotting with a specific anti-T7 antibody, expression of EGFP fluorescence, and qPCR analysis confirmed effective expression of these transcripts (Figures 7E–7H). As shown in Figures 7I and 7J, all the wild-type RNA transcripts but not the MRE mutants, were able to downregulate miR-34a-5p expression.

### Upregulation of miR-34a disrupts cell-cycle progression

**Figure 8:**
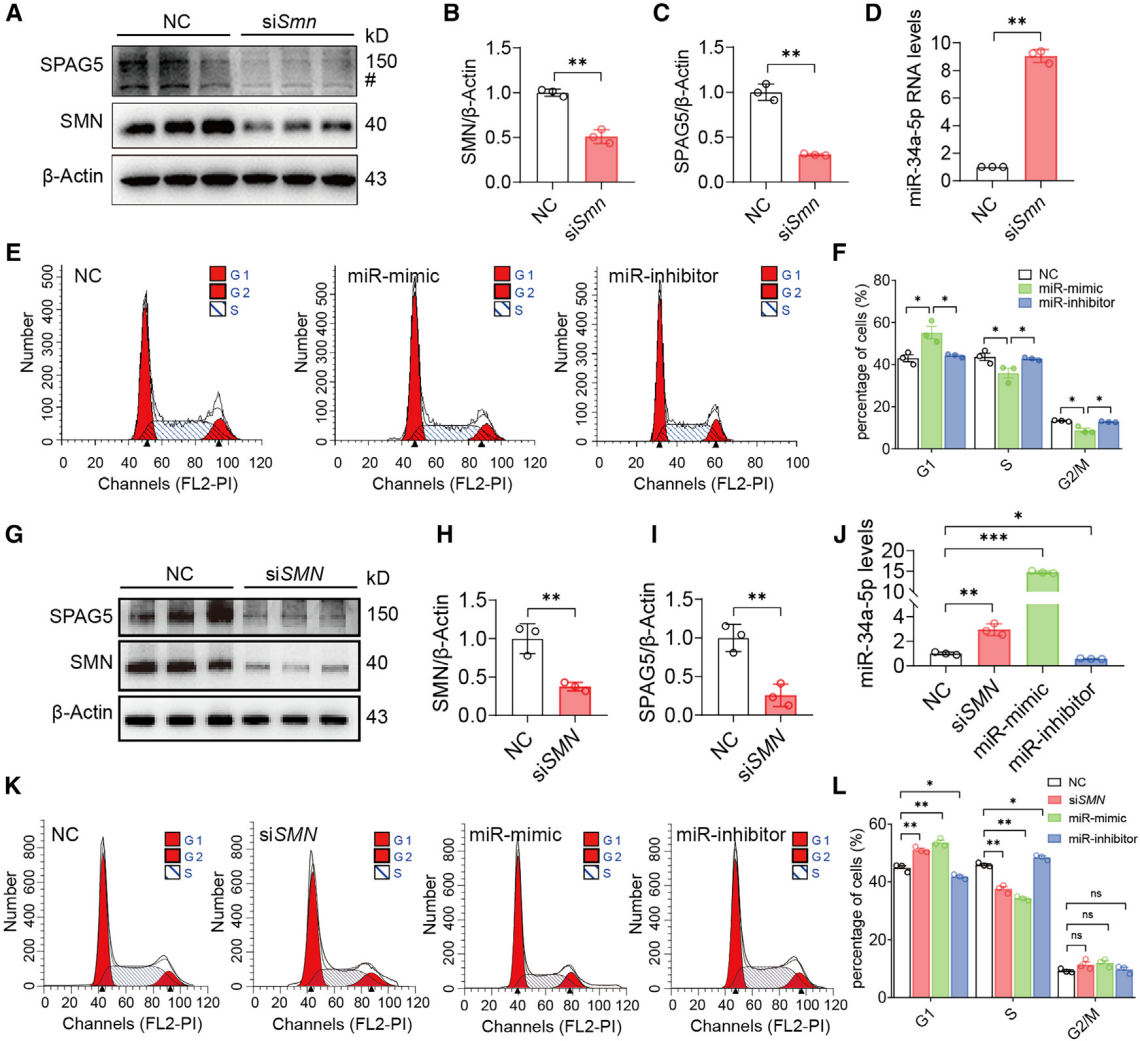
Decrease of SMN levels in both mouse and human cells potently enhanced expression of miR-34a-5p and disrupted cell cycle progression (A) A representative western blot showing that C2C12 cells transfected with si*Smn* reduced SMN and SPAG5 levels. NC-oligo was used as negative control and β-Actin used as loading control. An unknown shorter protein band, detected by the anti-SPAG5 antibody was also responded to si*Smn* treatment. (B and C) Quantitation of western blotting analysis as shown in (A). (D) RT-qPCR showing that knockdown of SMN in C2C12 cells transfected with si*Smn* resulted in robust upregulation of miR-34a-5p. (E) Flow cytometry analysis of C2C12 cells treated with miR-mimic, miR-inhibitor, or NC-oligo. miR-mimic treated cells were apparently arrested in G1 phase. (F) Quantitation of cells in each phase is shown on right. (G–I) Expression of SMN and SPAG5 in AC16 cells transfected with an siRNA against *SMN1/2* (si*SMN*) was analyzed by western blotting. Non-related NC-oligo (NC) was used as negative control. Protein levels were quantitated using β-Actin as internal control. (J) Expression changes of miR-34a-5p in AC16 cells transfected with si*SMN,* miR-mimic, miR-inhibitor, and NC-oligo, respectively. (K) Flow cytometry analysis of AC16 cells treated with si*SMN,* miR-mimic, miR-inhibitor, or NC-oligo. Cells transfected with si*SMN* and miR-mimic cells were apparently arrested in G1 phase. (L) Quantitation of cells in each phase is shown on right. For all samples, *n* = 3, ∗*p* < 0.05, ∗∗*p* < 0.01, ∗∗∗*p* < 0.001.

Overexpression of miR-34a is involved in multiple cellular defects such as cell-cycle arrest and apoptosis.^45^^,^^46^ We hypothesized that miR-34a-5p is a downstream target of SMN deficiency and plays a crucial role in cardiac abnormalities observed in the mouse model. To test our hypothesis, we first validated the cause-and-effect relationship between SMN deficiency and miR-34a-5p deregulation in C2C12 cells. In cells transfected with a specific siRNA against the *Smn* gene (si*Smn*), SMN protein levels were reduced to less than 50%; meanwhile, expression of miR-34a-5p was increased by approximately 10-fold and SPAG5, a protein known to play a pivotal role in cell cycle and proliferation processes, were downregulated to approximately 30% (Figures 8A–8D).

After establishment of miR-34a-5p being sensitive to SMN level changes in cultured cells, we next asked whether overexpression of the miRNA alone is sufficient to induce cell-cycle arrest. C2C12 cells were transfected with miR-mimic and the effect of miR-34a-5p overexpression on cell-cycle progression were analyzed. Cell number in G1 phase was dramatically increased, whereas the cell numbers in S and G2/M phases were markedly decreased (Figures 8E and 8F). We also tested miR-inhibitor and unexpectedly, in the miR-inhibitor-treated cells, the cell cycle was not obviously affected (Figure 8F). It is possible that a potential compensatory mechanism was activated to maintain cell mitosis after miR-34a-5p inhibition, which requires further investigation. Nevertheless, our data demonstrate that upregulation of miR-34a-5p affects cell cycle progression of the cell line, supporting our conclusion that upregulation of miR-34a-5p is critical in cardiac pathology in SMA mice.

We next asked if the detected upregulation of miR-34a-5p in SMN-deficient mouse cells is relevant to human cells. SMN levels in human AC16 cardiomyocytes transfected with a specific siRNA against the protein were decreased to approximately 35%, and indeed we observed a more than 2-fold increase in miR-34a-5p levels and a robust near 4-fold decrease in SPAG5 levels (Figures 8G–8J). Knockdown of SMN to about 34% also resulted in a comparable 2-fold increase in miR-34a-5p levels in HEK293 cells (Figures S15A–S15D). Furthermore, we performed flow cytometry to assess the effects of SMN knockdown, miR-mimic, and miR-inhibitor on cell-cycle progression in the two human cell lines. Both cell types transfected with miR-mimic showed a similar pattern of cell-cycle progression to that by siRNA knockdown of SMN, with an increased proportion of cells in G1 phase and a decreased proportion in the S phase, whereas treatment of miR-inhibitor had an opposite effect with reduced cell number in G1 phase (Figures 8J–8L and S15D–S15F).

## Discussion

High-throughput microarray and RNA-seq technologies have been widely used to explore key genes downstream of SMN deficiency in SMA patients’ cells and mouse models.^22^^,^^35^^,^^47^^,^^48^^,^^49^^,^^50^^,^^51^^,^^52^^,^^53^ However, the molecular basis how lack of SMN causes motor neuron death remains largely unknown. The findings of widespread defects in non-neural tissues are adding to the complexity of understanding of the disease. In the present study, we conducted a whole transcriptome analysis using the severe Taiwan mouse model to explore deregulated mRNAs and ncRNAs in the spinal cord and two non-neural tissues heart and liver at two early postnatal days. We identified a large number of DE-mRNAs, DE-lncRNAs, DE-circRNAs, and DE-miRNAs. Based on GO term analysis, detected DEGs are involved in various cellular processes such as cell-cycle progression, neuronal development and function, and blood-related processes. We also constructed six ceRNETs and uncovered a key miRNA, miR-34a, that is deregulated in all tissues. Multiple novel targets of the miRNA functioning as ceRNAs in the heart were defined. To the best of our knowledge, this is the first comprehensive study on both coding and ncRNAs differentially expressed in multiple tissues of an SMA mouse model. Our data not only offer potential in-depth explanations for widespread defects observed in neural and non-neural tissues in the context of SMA but also serve as a valuable resource for further investigation of the pathogenesis of the disease.

Investigations of DEGs in multiple tissues at presymptomatic and early symptomatic stages provide clues on which tissue is affected more severely or earlier than others in a multi-organ condition. We observed that at P1, the numbers of four differently expressed RNA types are higher in the heart of SMA mice than those in the spinal cord and liver. The numbers of DE-mRNAs and DE-miRNAs in spinal cord samples at P1 were merely 51 and 21, respectively, strikingly lower than those in the two non-neural tissues at P1, particularly heart. However, the differences between numbers of DEGs in all P4 tissues become much less prominent. In addition, the constructed P1/P4 heart ceRNETs contains 958 and 1,689 DEGs, respectively, far more than 160 and 1,303 in P1/P4 spinal cord ceRNETs, respectively, and 305 and 1,333 in P1/P4 liver ceRNETs, respectively. These data suggest that heart is a tissue with early onset of gene perturbations while spinal cord is affected relatively late but with a quick catchup in the days afterward. Moreover, GO-BP term analysis indicates that enriched DE-mRNAs in P1 spinal cord samples are mainly associated with blood healing and circulation, further supporting the presence of serious defects in the cardiovascular system. Our data are consistent with our previous study that revealed severe cardiac abnormalities in this mouse model.^32^ The GO-BP term analysis also surprisingly revealed highly enriched DEGs associated with neuronal development and function in P4 liver samples. Considering these genes having much lower expression levels compared with hepatocyte markers (Figure S16 and Table S3), their expression alterations may have little biological significance. However, it confirms that neuron-related genes are readily affected by a lack of SMN.

One of our important findings is global deregulation of miR-34a, a ubiquitously expressed miRNA. Both strands of miR-34a are active miRNAs with the guide 5p strand as one of the most studied miRNAs. miR-34a-5p is a versatile regulator of hundreds of genes and involved in various cellular processes, particularly in cell proliferation and cell death.^54^^,^^55^ It plays an inhibitory role on cell proliferation via multiple pathways by targeting a plethora of genes such as those encoding cyclins and cyclin-dependent kinases (CDKs).^56^ miR-34a also inhibits epithelial-mesenchymal transition (EMT) through targeting, e.g., genes encoding EMT-associated transcription factors such as *SNAI1*. Therefore, miR-34a, which itself is deregulated in various cancer types, has been recently considered a tumor suppressor. miR-34a-3p is less well elucidated and several recent *in vitro* studies suggest that it has similar effects on cell proliferation and apoptosis as its guide strand.^57^^,^^58^ Although miR-34a overexpression has been pursued as an approach to treat cancer, it is apparently detrimental for early tissue development, which is characterized by rapid cell proliferation and differentiation. For example, postnatal growth of mouse heart undergoes three stages: hypoplasia until P4, rapid hypertrophy from P5 to P15, and slow hypertrophy from P16 onwards.^59^ Our RT-qPCR analysis of miR-34a in eight tissues revealed robust increase in heart and spleen, but moderate in liver and limited in spinal cord (Figure S3). Consistent with these data, our GO-BP terms analysis of DEGs in the three tissues shows that only P4 heart DEGs are substantially related to cell cycle. We previously uncovered that downregulation of *Birc5* contributes of postnatal cardiac cell-cycle arrest of the same mouse model.^32^ However, the gene that causes *Birc5* downregulation has yet to be discovered. Several lines of evidence support miR-34a being the upstream gene of *Birc5*. First, it has been recently established that miR-34a plays a vital role in myocardial physiology and pathophysiological processes and is considered as a promising therapeutic target to treat cardiovascular diseases.^60^ Second, our overexpression study in a myoblast cell line with miR-mimic confirmed that miR-34 alone is sufficient to disrupt cell-cycle progress. Third, multiple prior studies identified miR-34a as a negative regulator of Survivin; particularly, a recent study revealed that *Birc5* is a direct target of miR-34a.^42^ Moreover, our RNA-seq data also identified *Birc5* being markedly downregulated in P4 heart samples. Unfortunately, the two miRNA databases used in this study have not been updated to include *Birc5* as a target of miR-34a and thus it is not shown up in the miR-34a ceRNETs. Nonetheless, our data highlight that miR-34a is a key link downstream of SMN deficiency and upstream of *Birc5* expression.

One of the mysteries puzzling us is that we did not detected downregulation of known targets of miR-34a associated with cell-cycle progress such as CDKs in the P4 heart samples, which may reflect differences in contexts such as cell types or medical conditions. Instead, we identified at least two coding genes, *Spag5* and *Hjurp*, as well as two ncRNAs, lncRNA00138536 and circRNA007386, as direct targets of the miRNA. It is not surprising that *Spag5* was the most responsive gene targeted by miR-34a in our study as it harbors three MREs of the miRNA. SPAG5, also known as Astrin, is a microtubule-associated protein that plays a crucial role in the formation of mitotic spindles and chromosome segregation.^61^^,^^62^ SPAG5 associates with the centrosome via interacting with Ninein and plays an important role in maintaining the integrity of the centrosome and spindle poles, particularly during the S and G2 phases of the cell cycle.^63^ In line with this, we observed that the cell cycle of C2C12 cells was arrested in the G1 phase after transfection with miR-mimic, which leads to a dramatic decrease of SPAG5 expression and marked reduction of the number of cells in the S and G2 phases, demonstrating a functional linkage between miR-34a-5p and *Spag5*. The circular ceRNA, circRNA007386, is derived from *RyR2*, a ryanodine receptor gene via its aberrant splicing. The RyR2 receptor is primarily expressed in the heart and responsible for rapid release of Ca^2+^ from the sarcoplasmic reticulum/endoplasmic reticulum and subsequent activation of intracellular ion channels, a critical step in excitation and contraction coupling in skeletal and cardiac muscles.^64^^,^^65^ Interference with Ca^2+^ signaling or downstream pathways often disrupts cell-cycle progression.^66^ Therefore, circRNA007386 is also a potential contributor to cardiac cell-cycle arrest observed in severe SMA mice. The other non-coding ceRNA, lncRNA00138536, did not align with any known gene. Future studies are required to determine the characteristic features and biological functions of the two ncRNAs. Overall, our data illustrate miR-34a-5p as a major player in cardiac pathologies of the SMA mouse model with *Birc5*, *Spag5*, circRNA007386, and potentially lncRNA00138536 as important downstream targets, although we do not rule out the possibilities that miR-34a or other factors may contribute to cardiac abnormalities in SMA mice via distinct pathways.

Our data also offer an explanation for the structural and functional abnormalities observed in the spleen of SMA mice in a previous study, in which Khairallah et al.^67^ reported that SMN deficiency selectively impacts postnatal development and size of spleen in three SMA mouse models, resembling the heart defects we observed.^32^ To our knowledge, heart and spleen are the only two organs that so far have been reported as undersized in mouse models, consistent with our observation that heart and spleen were the only two tissues presenting with over 5-fold upregulation of miR-34a-5p (Figure S3). Although we have not examined cell-cycle progression in the spleen of SMA mice, it is reasonable to postulate that the postnatal defects in this tissue were attributable at least in part to cell-cycle arrest due to upregulation of miR-34a. Moderate perturbation of miRNAs usually causes no obvious phenotypes. This explains why we and others did not observe cell-cycle disruption in other tissues such as liver and spinal cord in early postnatal days.

In this study, miR-34a-3p was moderately upregulated at P1 in the spinal cord, but unlike the non-neural tissues, the expression pattern was quickly reversed at P4, suggesting a mechanism in action preventing its elevation in the tissue. However, spinal cord tissue consists of different cell types and we do not rule out the possibility that certain underpopulated cell types may share the same regulatory mechanism downstream of SMN deficiency as in non-neuronal tissues such as heart. Interestingly, consistent with our data, a recent study unveiled downregulation of miR-34a in patient-derived iPSC-differentiated motor neurons and SMNΔ7 mouse model.^22^ The authors also reported partial rescues of motor neuron-related phenotypes after intravenous administration of scAAV vector serotype 9 expressing miR-34a. However, no survival extension was described in the study. We believe, overexpression of miR-34, while partly benefiting motor neuron survival and integrity, seriously affects non-neural tissues, particularly the heart and spleen of neonatal mice.

While our work represents the first whole transcriptome study of heart defects in the Taiwanese model, gene expression alterations, particularly U12-dependent splicing, in the spinal cord, muscle, and liver of the same model have been previously explored using RNA-seq.^35^ When comparing our data with the data reported by Doktor et al.,^35^ we found approximately 20% overlapping DE-mRNAs between the two studies at the late time point, although the time point was slightly different (P4 vs. P5) (Figure S17A). Notably, *Spag5* was identified as a DEG in both studies (P4 heart and P5 muscle). These common DEGs overcame the bias introduced by differences in sampling ages and library construction methods, indicating a high reliability. Moreover, when comparing DE-miRNAs from heart, liver, and spinal cord tissues in our study with the muscle biopsy data obtained from SMA patients in an early study,^21^ we found approximately 10% overlapping DE-miRNAs between the two studies, with the most overlapping miRNAs detected in the heart (Figure S17B). These overlapping DE-miRNAs common between mice and humans warrant further investigation.

Abnormal expression of specific miRNAs has been delineated in various neurodegenerative diseases including SMA, Alzheimer’s disease, and amyotrophic lateral sclerosis.^68^^,^^69^^,^^70^ In particular, a few neuron-specific miRNAs have been implicated as contributors to motor neuron death of SMA.^71^ For example, miR-9, which regulates the heavy neurofilament subunit, is downregulated in an SMA model.^72^ However, we found that miR-9-3p and miR-9-5p were differentially expressed in the liver at P1 and P4, but not in the spinal cord and heart. Moreover, no expression change in any examined tissue was observed for other miRNAs such as miR-132 and miR-206, which have been previously reported as deregulated in SMN-depleted cell lines or SMA animal models.^20^ One of the reasons that cause the discrepancy is the time points when the tissue samples were collected. For example, Catapano et al.^23^ observed that miR-9, miR-132, and/or miR-206 were deregulated in spinal cord and muscle samples collected at P10, a near-death stage, as well as in serum samples at P7 but not in P2 samples in the same severe mouse model. On the other hand, we identified multiple DE-ncRNAs at early postnatal days that have never been reported previously including a plethora of lncRNAs and circRNAs. The potential roles of these new ncRNA targets downstream of SMN deficiency warrants further investigation.

In summary, using whole transcriptome sequencing, this study identified numerous differentially expressed coding and ncRNAs in the severe Taiwan mouse model. Analysis of DE-miRNAs in multiple tissues unveiled global upregulation of miR-34a in non-neuronal tissues, particularly a robust increase in the heart, which is responsible for cell cycle disruption observed in the tissue. Moreover, we defined the ceRNET axis, lncRNA008536/circRNA007386/miR34a/*Spag5*, shedding light into the molecular basis behind postnatal cardiac cell-cycle arrest in the mouse model. Although how the ceRNET axis is affected by lack of SMN has yet to be investigated, our data enhanced the understanding of the molecular pathogenesis of SMA and provide potential new avenues for early diagnosis and therapeutic intervention.

## Materials and methods

### Animals and sample collection

The severe Taiwanese mouse model (*Smn*^−/−^, *SMN2*^2TG/0^) was generated by crossing heterozygous knockout mice (*Smn*^+/−^) with mild Taiwanese mice (*Smn*^−/−^, *SMN2*^2TG/2TG^), which was a gift from Krainer laboratory at Cold Spring Harbor Laboratory and was originally purchased from the Jackson Laboratory (FVB.Cg*Smn1*^tm1Hung^Tg(*SMN2*)^2^Hung/J, stock number 005058).^29^ Mice were housed in a specific pathogen-free barrier facility at the Experimental Animal Center, Nantong University. All animal experiments were approved by the Laboratory Animal Ethics Committee of Nantong University (IACUC20170316-1001). Each group for tissue collection had three pups: two males and one female. Mice were sacrificed by CO_2_ asphyxiation; tissues were quickly placed in liquid N_2_ and stored at −80°C.

### RNA extraction, library construction, and sequencing

Total RNA was extracted using Trizol reagent (Thermo Fisher Scientific). The RNA integrity number (>8) of samples was determined using a Bioanalyzer 2100 system with RNA 6000 Nano kit (Agilent Technologies). RNA samples were first subjected to rRNA removal and RNA fragmentation, and then single-strand cDNAs were synthesized with random hexamers, followed by double-strand cDNA synthesis and purification. After end repair, A-tailing, adapter ligation, and size sorting of cDNA samples, the dUTP-marked strand was degraded by Uracil-DNA-Glycosylase (Vazyme) and PCR enrichment was performed to amplify the cDNA libraries of mRNAs and ncRNAs. We isolated 18–30 nt noncoding sRNAs from RNA samples using 15% polyacrylamide gel and cDNA libraries were prepared with the TruSeq sRNA sample preparation kit (Illumina). RNA-seq was performed with HiSeq 2500 platform (Illumina). Library insert sizes were detected with Agilent 2100 (Agilent Technologies) and quantitation was performed using RT-qPCR with CFX96 instrument (Bio-Rad Laboratories). The concentration of each library was more than 2 nM.

The whole transcriptome sequencing data reported in this study have been deposited to the NCBI SRA repository with accession numbers SRR 28204395–28204430 (RNA-seq) and SRR 28205788–28205823 (sRNA-seq) and NCBI BioProject repository with accession number PRJNA1076644.

### Sequencing data analysis

We used pheatmap R package^73^ and Euclidean distance matrix^74^ to perform hierarchical clustering analysis on gene expression profiles, and the results are represented by heat maps. Each column represents a sample, and each row represents a log_2_ ratio value of a gene. The y axis in volcano plots represents the distributions of −log_10_ (*p* value) and the x axis represents the log_2_ ratio value.

To identify circRNAs, we used CircRNA_Finder^75^ and Bowtie2^76^ to align clean reads with the reference genome and used the back-splice algorithm to extract the junction of unmapped reads, followed by verification with Circbase.^77^ To collect the annotations of sRNAs, they were mapped to only one RNA category using the following priority rule: rRNAetc (in which GenBank > Rfam) > known miRNA > piRNA > repeat > exon > intron; rRNAetc includes rRNA, tRNA, snRNA, scRNA, and snoRNA deposited in the GenBank and Rfam databases as described.^78^

### Differential expression analysis, GO term analysis, and prediction of ceRNETs

RNA-seq data of the three tissues between SMA and control mice were analyzed using both significance levels and fold change to define differentially expressed RNAs (DE-RNAs). The significance levels were estimated with Student’s t-test and adjusted with the Benjamini-Hochberg method. For all types of ncRNAs, those with an absolute fold change of more than 2 and a *p* value of less than 0.05 were considered differentially expressed. For more abundant mRNAs, we used absolute fold change of more than 2 and a q value of less than 0.05, a more stringent threshold, to identify DE-mRNAs. The enriched GO terms of all DEGs were analyzed using the GO database (http://www.geneontology.org/), and the number of genes in each term was calculated. Hypergeometric tests were used to find GO entries that were significantly enriched in DEGs compared with the entire genome background. Identification of putative MREs in RNAs was performed by Targetscan^37^ with the context score percentile of more than 90 and miRanda software^38^ with the MRE score being 150 or greater and binding energy of less than −7, and targets shared by both software were extracted. DE-mRNAs, DE-lncRNAs, and DE-circRNAs corresponding with their respective DE-miRNAs were obtained; mechanism-specific miRNA-ceRNA regulatory networks and eventual ceRNETs were constructed by visual editing with Cytoscape software.^39^

### RT-qPCR and regular RT-PCR

RT-qPCR was performed using an SYBR RT-qPCR kit (Vazyme). In brief, reverse transcription reaction of 20 μL was conducted using mixed oligo (dT) and random primers for RNAs other than miRNAs, or special primers designed with the neck loop method for miRNAs, followed by removal of contaminating DNA. cDNAs were amplified with 45 cycles using specific primers and expression levels were calculated using the ΔΔ Ct method. *RNU6-1*, whose expression levels were not altered in all tissues based on our RNA-seq data, was used as internal control for miRNAs, and *Gapdh* for mRNAs, lncRNAs, and circRNAs. Primer sequence information is listed in Table S4 lncRNAs and circRNAs were validated by PCR using 2 × Taq Master Mix (Vazyme), followed by agarose gel electrophoresis of PCR products and sequencing. Sequences were blasted in the NCBI GenBank to confirm their identity and the cyclization site of each circRNA. circRNAs were also verified by the RNase R (Epicentre) degradation assay (Figure S13).

### FISH assay of heart tissue of SMA mice

Tissue samples were fixed with 4% formaldehyde at 4°C overnight and paraffin-embedded, 4-μm sections were cut. After dewaxing, rehydration, protease treatment, and denaturation at 65°C–70°C for 5 min, sections were incubated with Cy3-labelled miR-34a-5p probe (GenePharma) in a humid box at 37°C overnight. Next day, sections were treated with DAPI, sealed with fluorescent mounting tablet, and examined with a fluorescence microscope (Olympus BX51). The probe sequence is listed in Table S4.

### Plasmid construction

The *Spag5*, *Spag5* 3′ UTR, and lncRNA00138536 expression plasmids were constructed using the pCGT7 vector. The *Spag5* plasmid expresses not only mRNA but also N-terminal T7-tagged SPAG protein. circRNA007386 expression plasmid was constructed using the pLCDH-ciR vector and dual-fluorescence reporter plasmids using the pmiR-Report vector. Mutant plasmids were generated as previously described.^3^ The MRE mutation sequences in the *Spag5* 3′ UTR, lincRNA00138536, and circRNA007386 (both MREs mutated) were generated by replacing each MRE nucleotide with its complementary counterpart. Primer sequence information is shown in Table S4.

### Cell culture, transfection, luciferase assay, and flow cytometry

C2C12 and HEK293T cells were cultured in six-well plates in DMEM and AC16 cells in DMEM/F12 medium; all medium (Thermo Fisher Scientific) was supplemented with 10% (v/v) fetal bovine serum and antibiotics (100 U/mL penicillin and 100 μg/mL streptomycin). MiR-mimic, miR-inhibitor, si*Smn*, si*SMN*, NC-oligo, and MRE sequence oligos were purchased from Genepharma and sequence information is shown in Table S4. Synthetic oligos as well as plasmid(s) were transfected into cells using Lipofectamine 2000 (Thermo Fisher Scientific). At 48 h after transfection, cells were collected for RNA or protein sample extraction. For dual luciferase assay, cells were harvested at 48 after transfection and luciferase signals were assessed using the Dual Luciferase Reporter Gene Assay Kit (Beyotime). The Renilla signal was normalized to firefly signal. For flow cytometry, cells were fixed in 70% chilled ethanol for 30 min, and then stained with propidium iodide for 30 min, followed by fluorescence-activated cell sorting using Beckman Coulter FC500 (BD Biosciences). Distribution of cells in different cell-cycle phases was determined by ModFit LT software (Verity Software House).

### Western blotting

Protein samples from tissues or cells were separated by 10% SDS-PAGE and electroblotted onto polyvinylidene fluoride membranes, followed by blocking with 5% skim milk. Then the membranes were incubated with primary antibodies overnight at 4°C and secondary antibodies for 2 h at room temperature. The primary antibodies used were as follows: rabbit anti-SPAG5 antibody (Proteintech), rabbit anti-HJURP antibody (Proteintech), mouse anti-T7 tag (Beyotime), and mouse anti-β-Actin antibody (Santa Cruz Biotechnology). Secondary rabbit anti-mouse and goat anti-rabbit antibodies were purchased from Sangong. Protein signals were detected with the Tanon-5200 Multi Gel Imaging System (Tanon Science & Technology). The scanned images were imported into ImageJ software 7.0. The signals were normalized to β-Actin.

### Statistical analysis

Software SPSS 16.0 (SPSS, Inc) was used for statistical analysis of experimental results. Experimental data are presented as mean ± SD. The statistical significance of the differences between groups was analyzed using Student’s t test. A *p* value of less than 0.05 was considered statistically significant.

## Data availability

The data generated in this study are available upon request from the corresponding author.

## Acknowledgments

This work was supported by the 10.13039/501100001809National Natural Science Foundation of China (NSFC grants 81530035, 82073753, and 32271346 to Y.H).

## Author contributions

Y.H. designed the study. L.Wu, J.S., L.Wang, Z.C., Z.G., L.D., and R.Q. performed the experiments and analyzed data. Y.H., L.Wu, and L.Wang wrote the manuscript. Y.H., C.L., and Y.S. contributed to the material support of the study. All authors have read and approved the final manuscript.

## Declaration of interests

Authors declare no conflict of interest.

## Supplemental information

Document S1. Figures S1–S17 and Tables S3 and S4

Table S1. List of DEGs in all ceRNETs

Table S2. List of DEGs in miR-34a networks

Document S2. Article plus supplemental information
