# Supplementary material for: Whole-transcriptome sequencing in neural and non-neural tissues of a mouse model identifies miR-34a as a key regulator in SMA pathogenesis
Source: Mol Ther Nucleic Acids. 2025 Feb 20;36(2):102490. doi: 10.1016/j.omtn.2025.102490 (PMC11930137; doi:10.1016/j.omtn.2025.102490)
Supplement: Document S1. Figures S1–S17 and Tables S3 and S4 [file mmc1.pdf]

OMTN, Volume *36*

Supplemental information

Whole-transcriptome sequencing in neural and non-neural tissues of a mouse model identifies miR-34a as a key regulator in SMA pathogenesis

Liucheng Wu, Junjie Sun, Li Wang, Zhiheng Chen, Zeyuan Guan, Lili Du, Ruobing Qu, Chun Liu, Yixiang Shao, and Yimin Hua

Figure S1

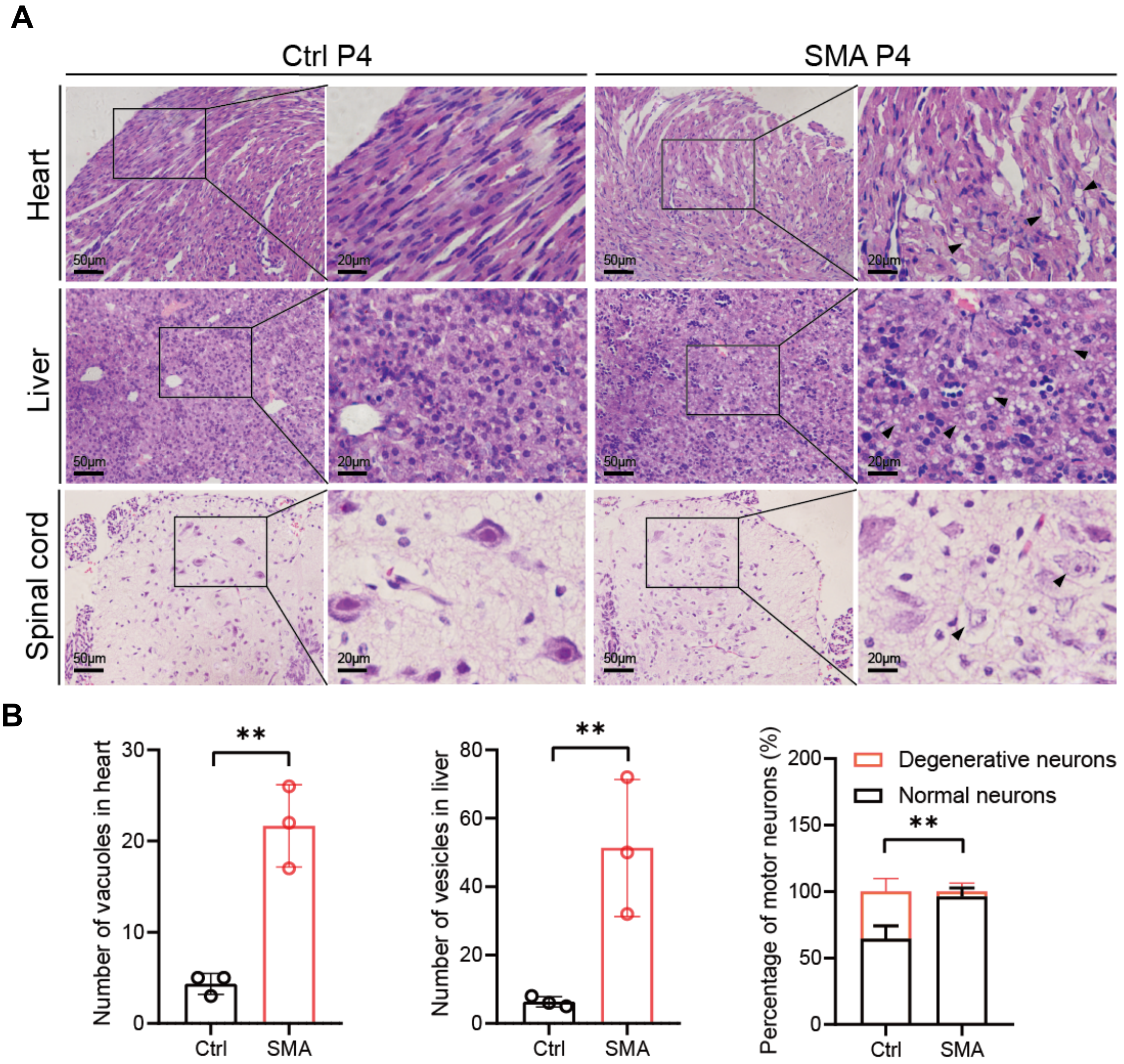

Figure S1. The hematoxylin and eosin (H&E) staining of heart, liver, and spinal cord tissues of SMA and heterozygous (Ctrl) mice.

(**A**) Tissues were collected from SMA mice (n = 3) at P4 and fixed with 4% formaldehyde at 4 ºC overnight. After washing in 0.01 M phosphate-buffered saline, tissues were embedded in paraffin blocks and 4-μm–thick sections were cut for H&E staining. The number of cells in heart tissue of SMA mice was relatively lower than that in heterozygous mice and tissue vacuolization (arrows) was observed. In the liver, a large number of lightly stained vesicles (arrows) were detected. As for the spinal cord, considerable motor neurons in the anterior horn displayed intranuclear vacuolization (arrows). Scale bar = 50 or 20 μm as indicated. (**B**) Quantitation of the number of vacuoles in cardiomyocytes per high-power field, the number of vesicles in hepatocytes, the percentage of degenerative neurons per high-power field. ** $p < 0.01$ (n = 3).

Figure S2

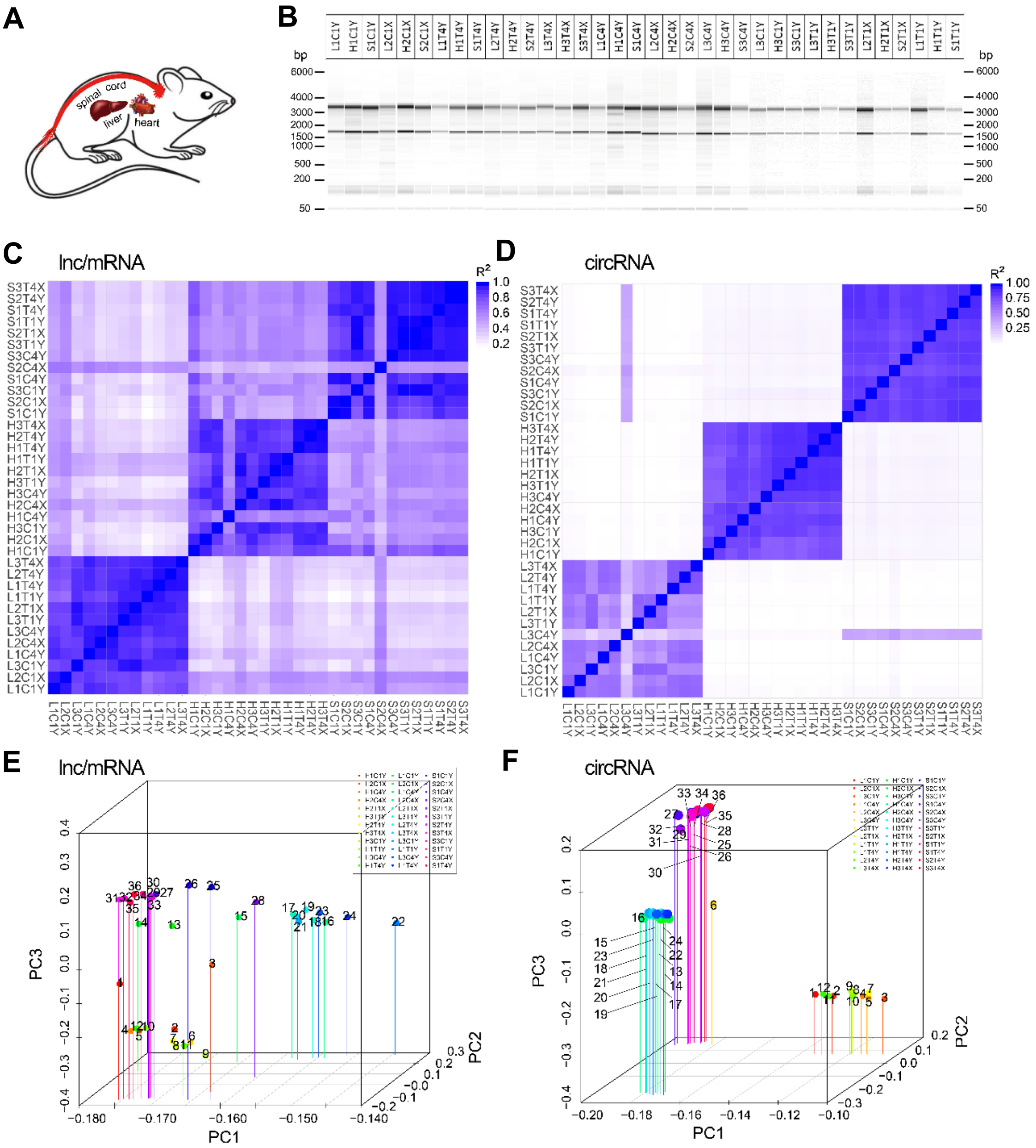
A
spinal cord
liver
heart
B
C
lnc/mRNA
D
circRNA
E
lnc/mRNA
PC1
PC2
PC3
F
circRNA
PC1
PC2
PC3

Figure S2. Quality analysis of the total RNA samples used in the present study. (**A**) The schematic diagram of mouse tissues used in the present study. (**B**) Capillary gel electrophoresis was performed to assess the quality of total RNA samples using Labchip GX (PerkinElmer, MA, USA). Each lane represents a sample, named as tissue-mouse #-mouse type-age-sex with L stands for liver, H heart, S spinal cord, C control mice, T SMA mice, Y male, and X female. For example, L1T4Y represents liver tissue collected from #1 SMA mouse at P4 that was male. (**C** and **D**) The Pearson correlation analysis of lncRNAs, mRNAs and circRNAs in mouse samples. (**E** and **F**) Three-dimension principal component analysis (PCA) plots with 36 balls (for all 36 tissue samples) shown. The principal components were converted from original variable data using orthogonal transformation to achieve data dimensionality reduction. The position of each ball in PCA plots represents the value of the sample on each principal component, and different colors represent different samples. PC1, PC2, and PC3 represent different calculation methods for gene expression profiles in matrix form, respectively.

Figure S3

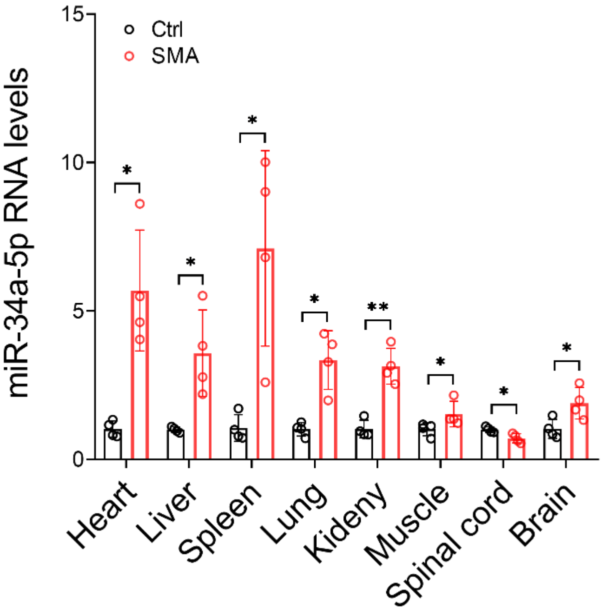

Figure S3. Expressions of miR-34a-5p in eight tissues of SMA mice.
Examination of miR-34a-5p expression levels in eight tissues of SMA mice, including heart, liver, spleen, lung, kidney, muscle, spinal cord and brain. * $p < 0.05$, ** $p < 0.01$, $n = 4$.

Figure S4

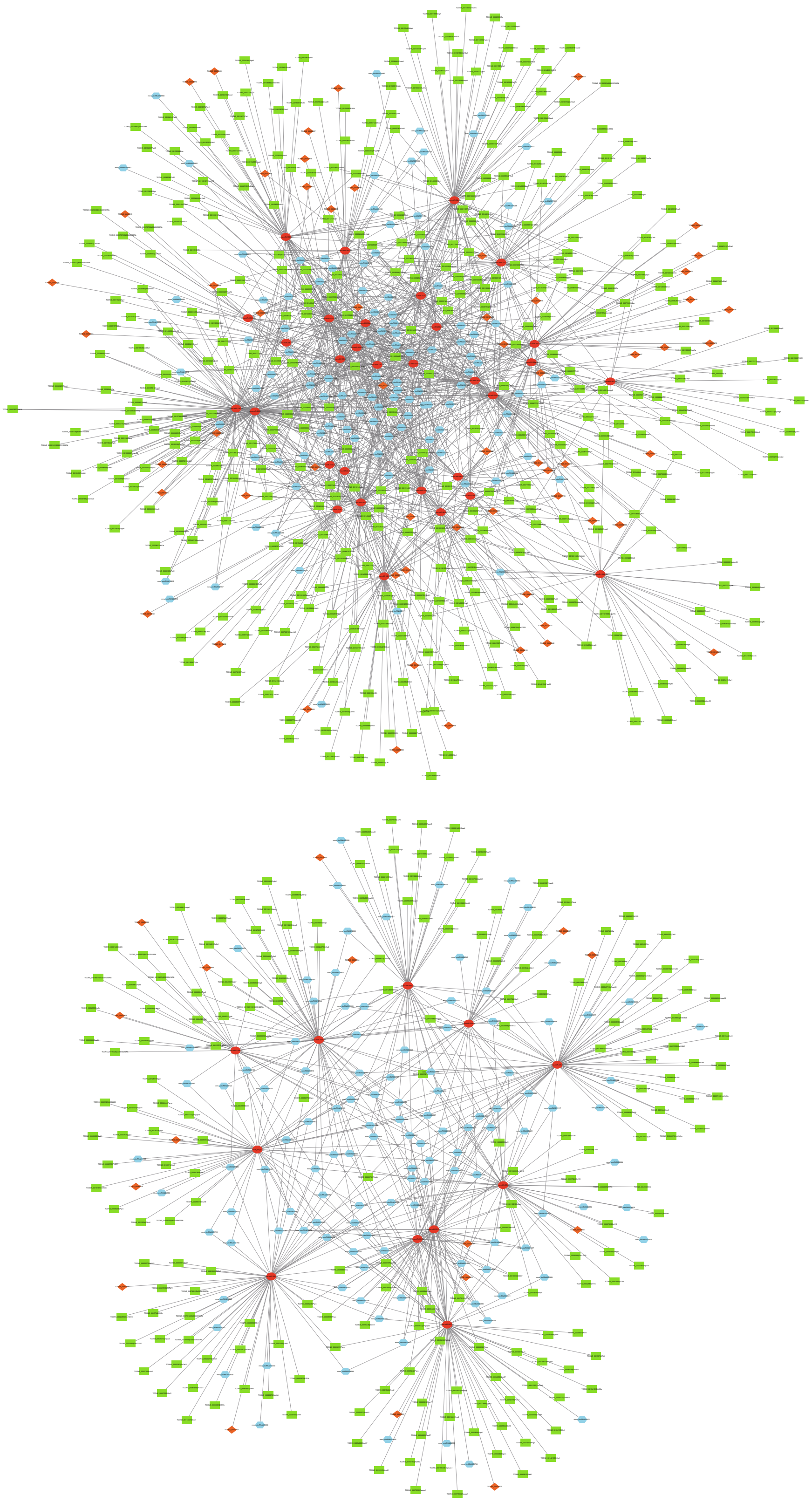

Figure S4. lncRNA-circRNA-miRNA-mRNA networks for P1 heart samples of SMA mice.

The left network shows decreased miRNAs and corresponding increase of their target ceRNAs, while the right one shows increased miRNAs and corresponding decrease of their target ceRNAs. All ceRNAs detected in P1 heart tissues are shown in **Figure S10** and **Table S1**. Rectangles represent mRNAs, diamonds represent lncRNAs, hexagons represent circRNAs, and ellipses represent miRNAs.

Figure S5

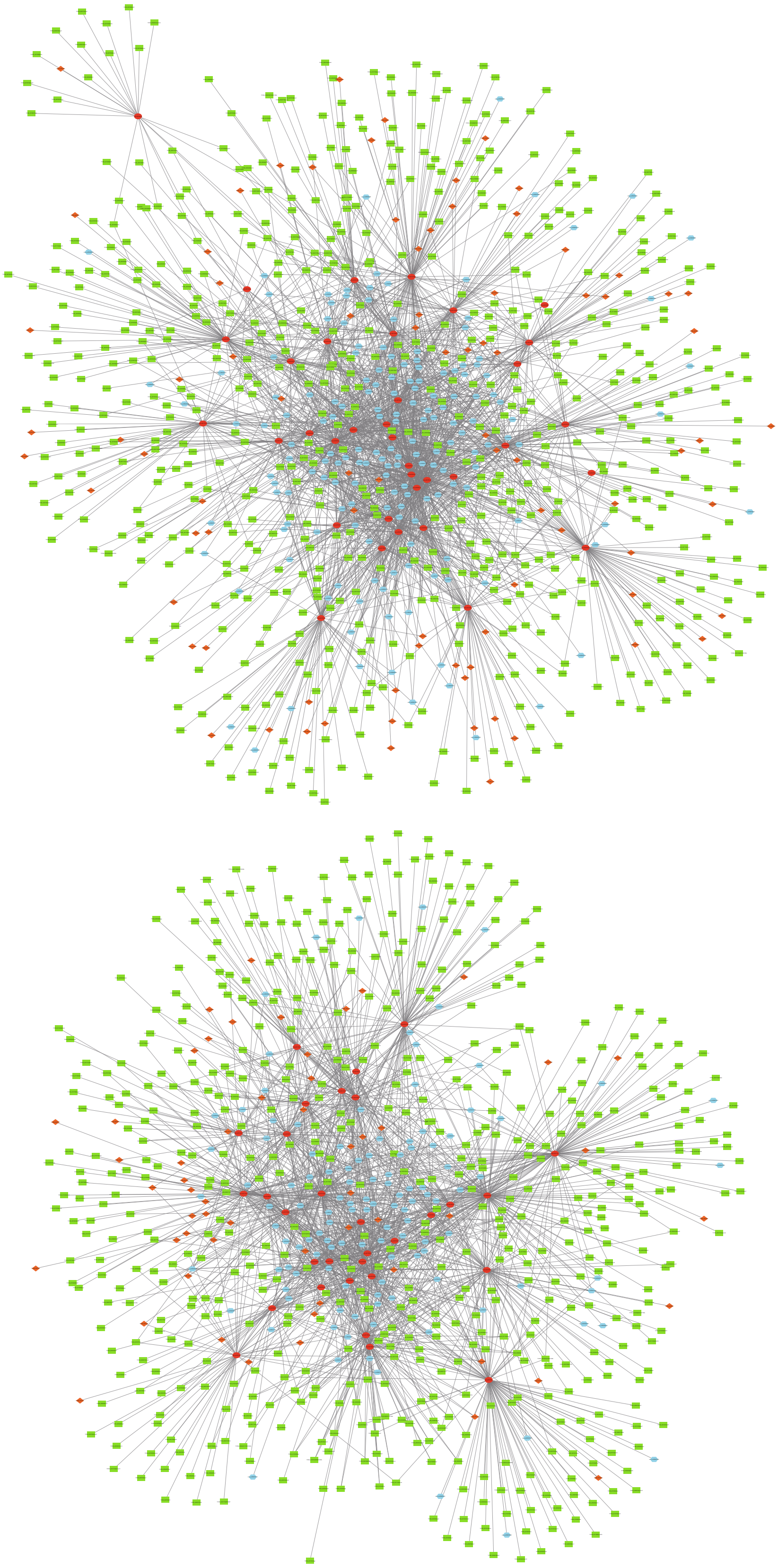

Figure S5. lncRNA-circRNA-miRNA-mRNA networks for P4 heart samples of SMA mice.

The left network shows decreased miRNAs and corresponding increase of their target ceRNAs, while the right one shows increased miRNAs and corresponding decrease of their target ceRNAs. All ceRNAs detected in P4 heart tissues are shown in **Figure S10** and **Table S1**. Rectangles represent mRNAs, diamonds represent lncRNAs, hexagons represent circRNAs, and ellipses represent miRNAs.

Figure S6

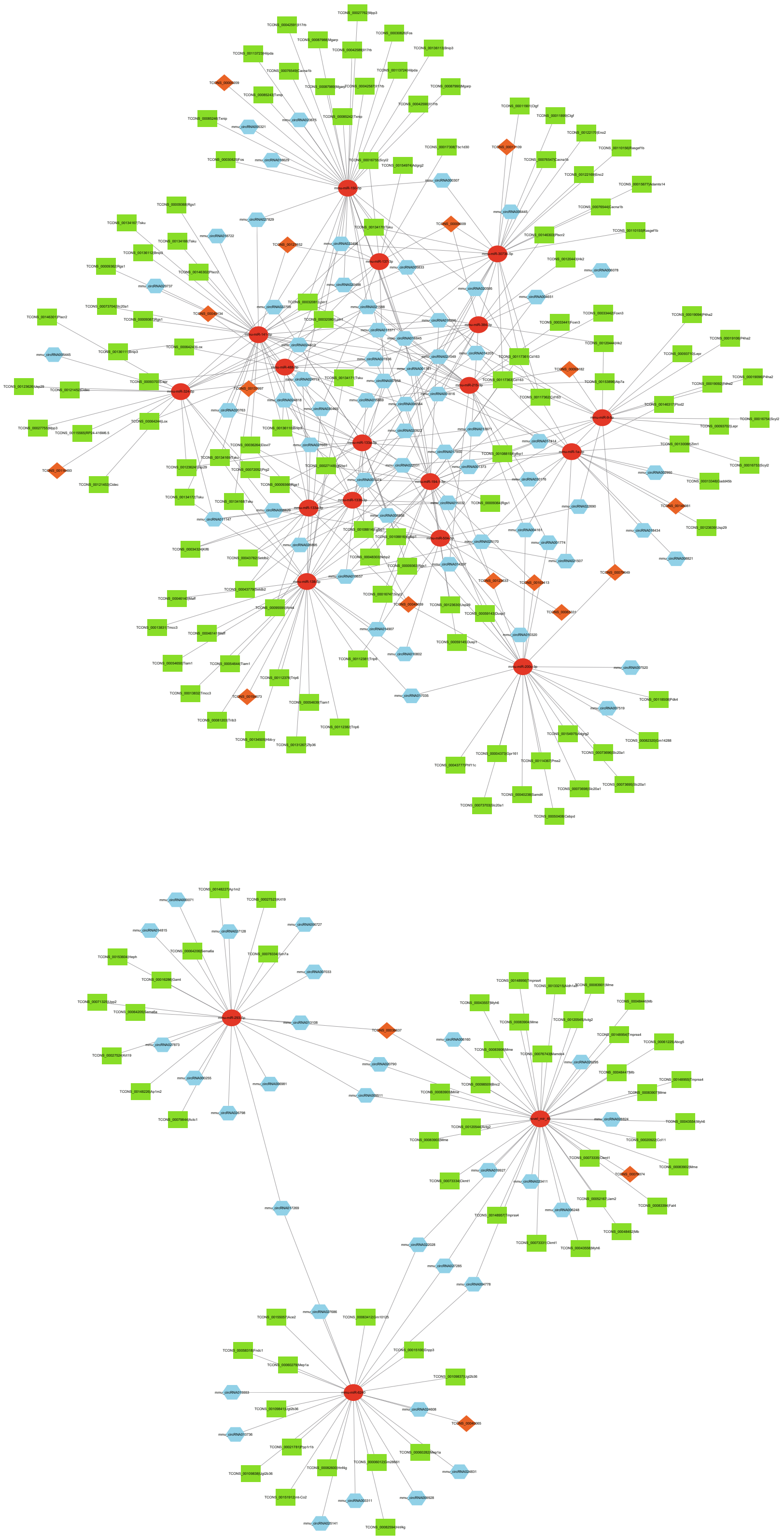

Figure S6. lncRNA-circRNA-miRNA-mRNA networks for P1 liver samples of SMA mice.

The left network shows decreased miRNAs and corresponding increase of their target ceRNAs, while the right one shows increased miRNAs and corresponding decrease of their target ceRNAs. All ceRNAs detected in P1 liver tissues are shown in **Figure S10** and **Table S1**. Rectangles represent mRNAs, diamonds represent lncRNAs, hexagons represent circRNAs, and ellipses represent miRNAs.

Figure S7

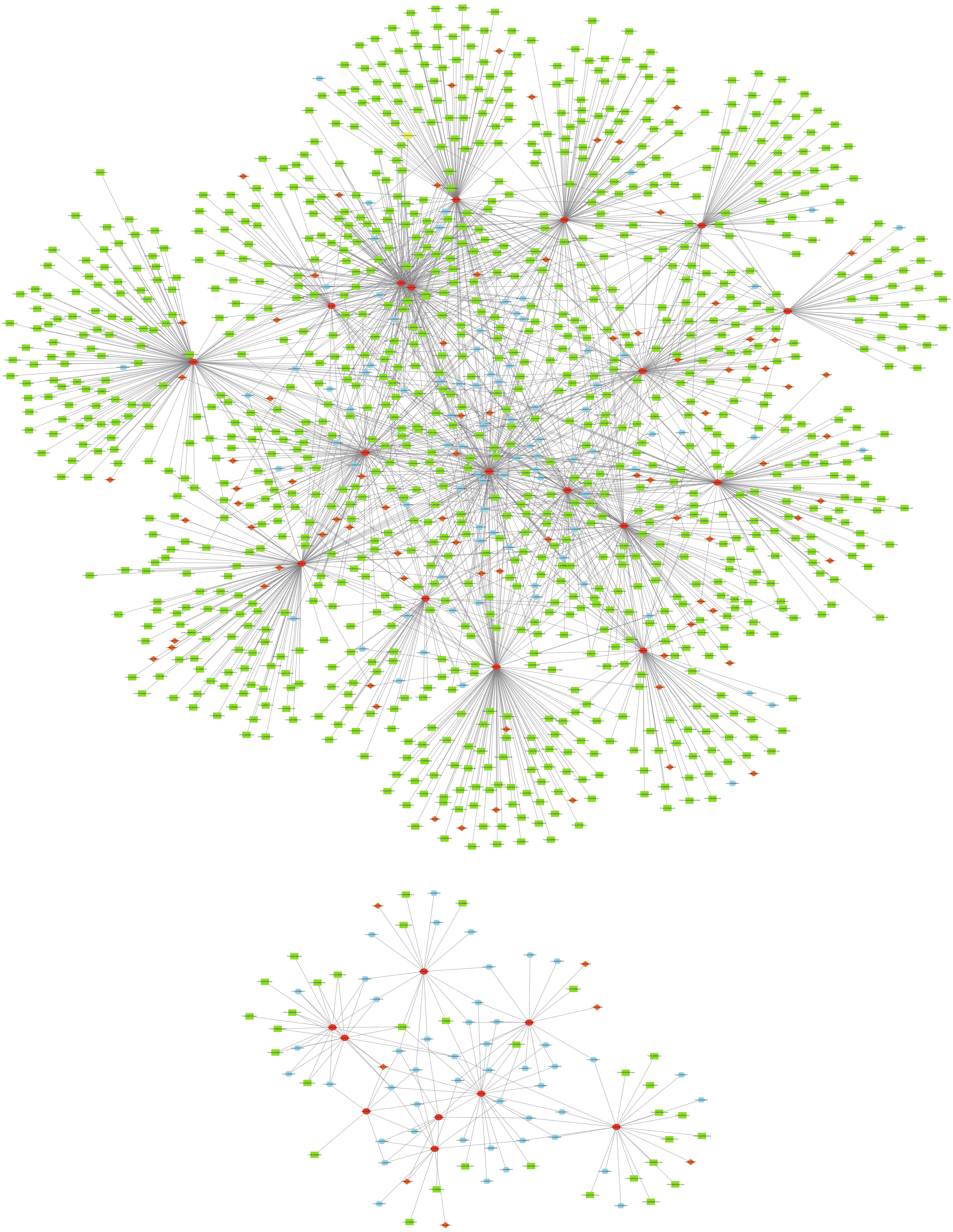

Figure S7. lncRNA-circRNA-miRNA-mRNA networks for P4 liver samples of SMA mice.

The left network shows decreased miRNAs and corresponding increase of their target ceRNAs, while the right one shows increased miRNAs and corresponding decrease of their target ceRNAs. All ceRNAs detected in P4 liver tissues are shown in **Figure S10** and **Table S1**. Rectangles represent mRNAs, diamonds represent lncRNAs, hexagons represent circRNAs, and ellipses represent miRNAs.

Figure S8

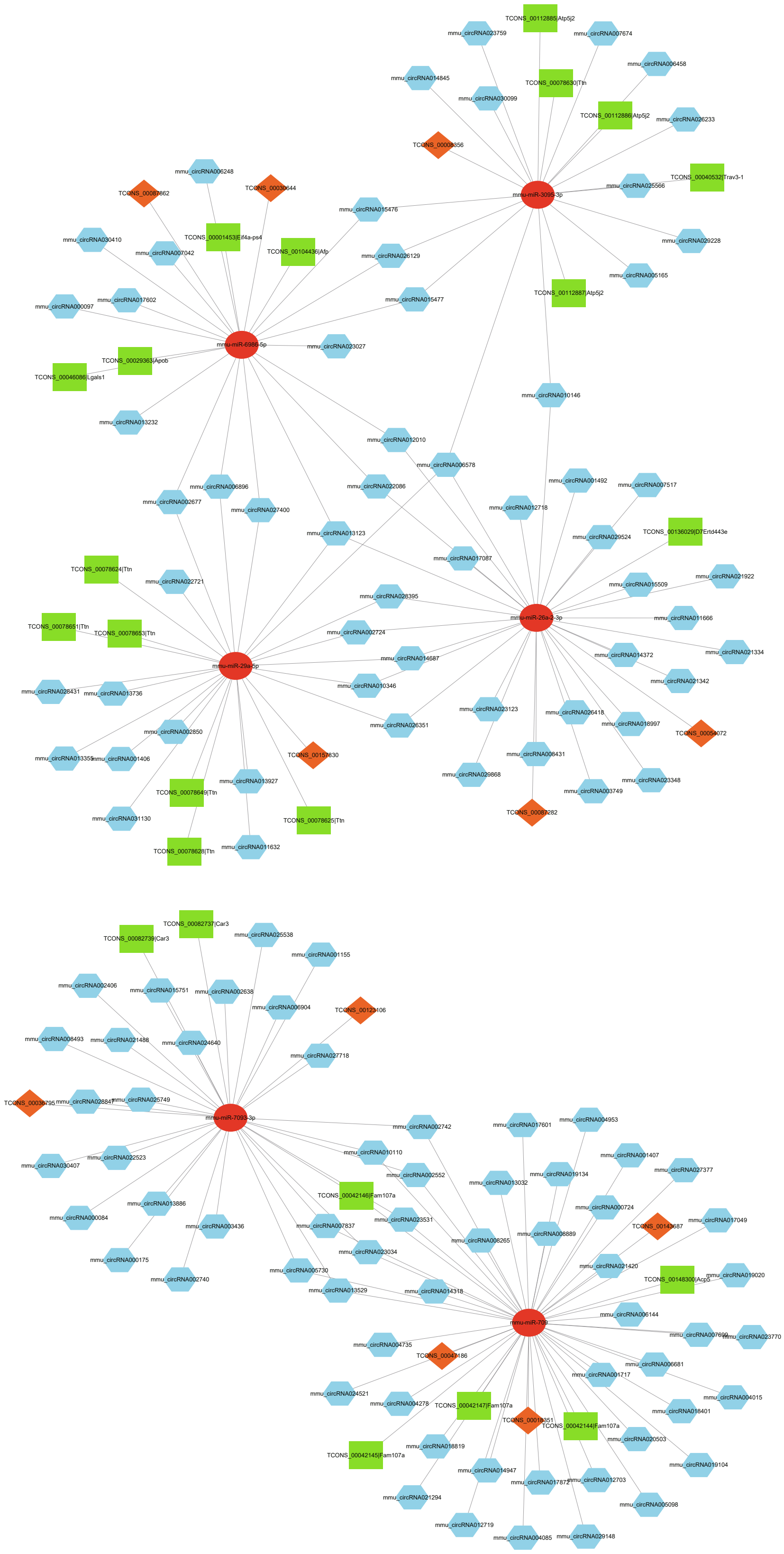

Figure S8. lncRNA-circRNA-miRNA-mRNA networks for P1 spinal cord samples of SMA mice.

The left network shows decreased miRNAs and corresponding increase of their target ceRNAs, while the right one shows increased miRNAs and corresponding decrease of their target ceRNAs. All ceRNAs detected in P1 spinal cord tissues are shown in **Figure S10** and **Table S1**. Rectangles represent mRNAs, diamonds represent lncRNAs, hexagons represent circRNAs, and ellipses represent miRNAs.

Figure S9

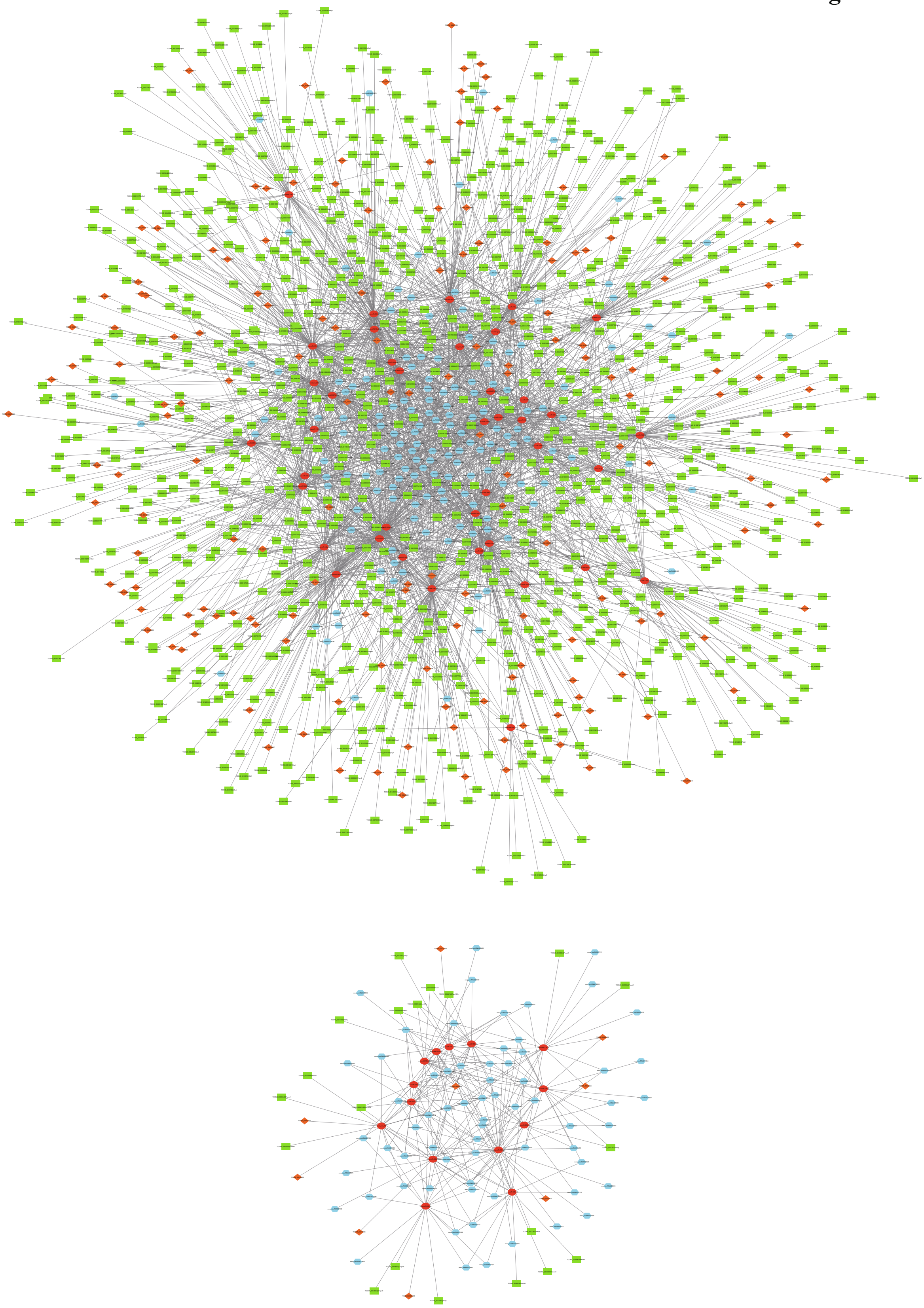

Figure S9. lncRNA-circRNA-miRNA-mRNA networks for P4 spinal cord samples of SMA mice.

The left network shows decreased miRNAs and corresponding increase of their target ceRNAs, while the right one shows increased miRNAs and corresponding decrease of their target ceRNAs. All ceRNAs detected in P4 spinal cord tissues are shown in **Figure S10** and **Table S1**. Rectangles represent mRNAs, diamonds represent lncRNAs, hexagons represent circRNAs, and ellipses represent miRNAs.

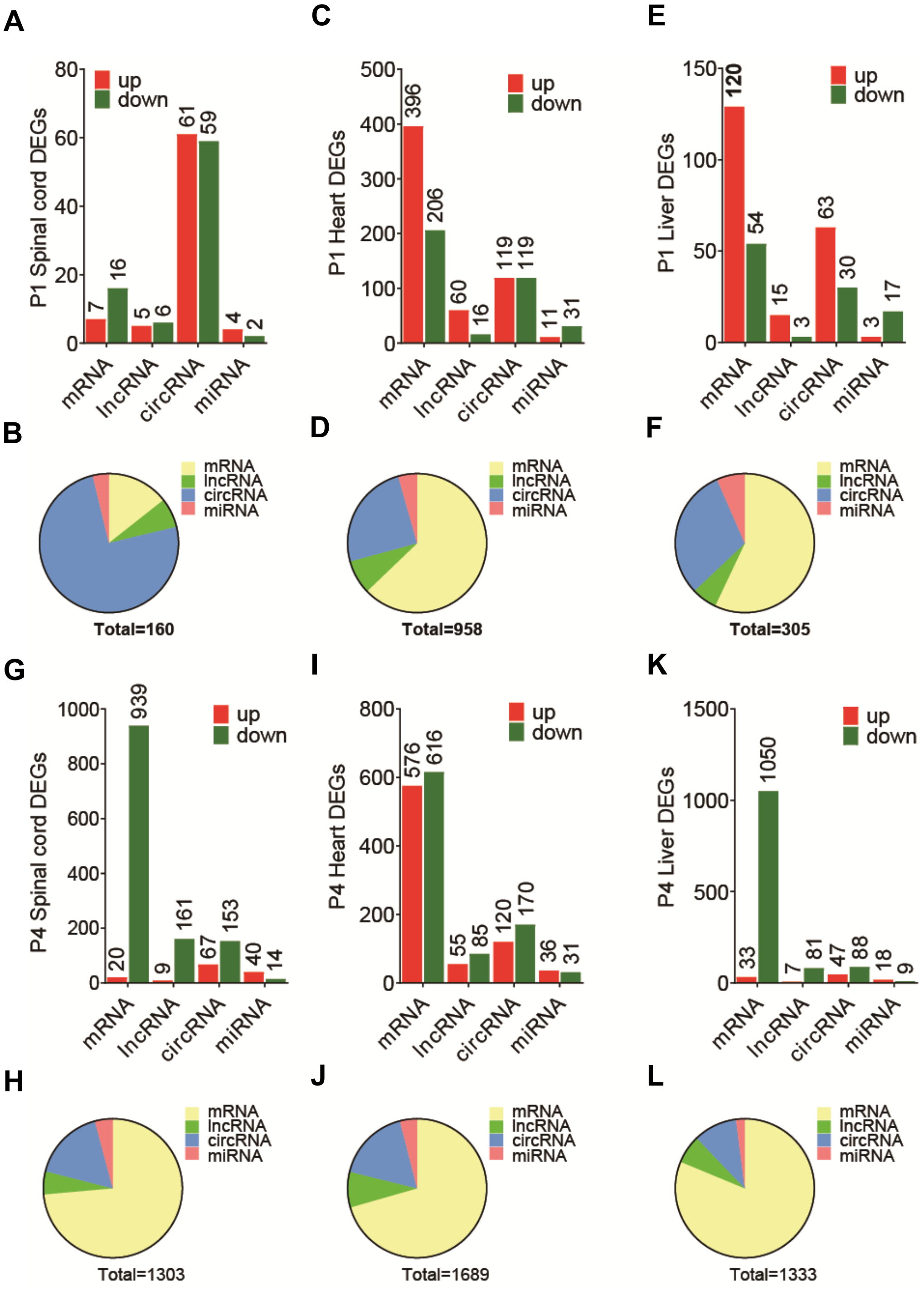

Figure S10. The numbers of differentially expressed ceRNAs in heart, liver, and spinal cord tissues of SMA mice.

Histograms and sector diagrams showing the numbers of differentially expressed ceRNAs of P1 and P4 spinal cord (**A**, **B**, **G**, and **H**), heart (**C**, **D**, **I**, and **J**), and liver (**E**, **F**, **K**, and **L**) samples.

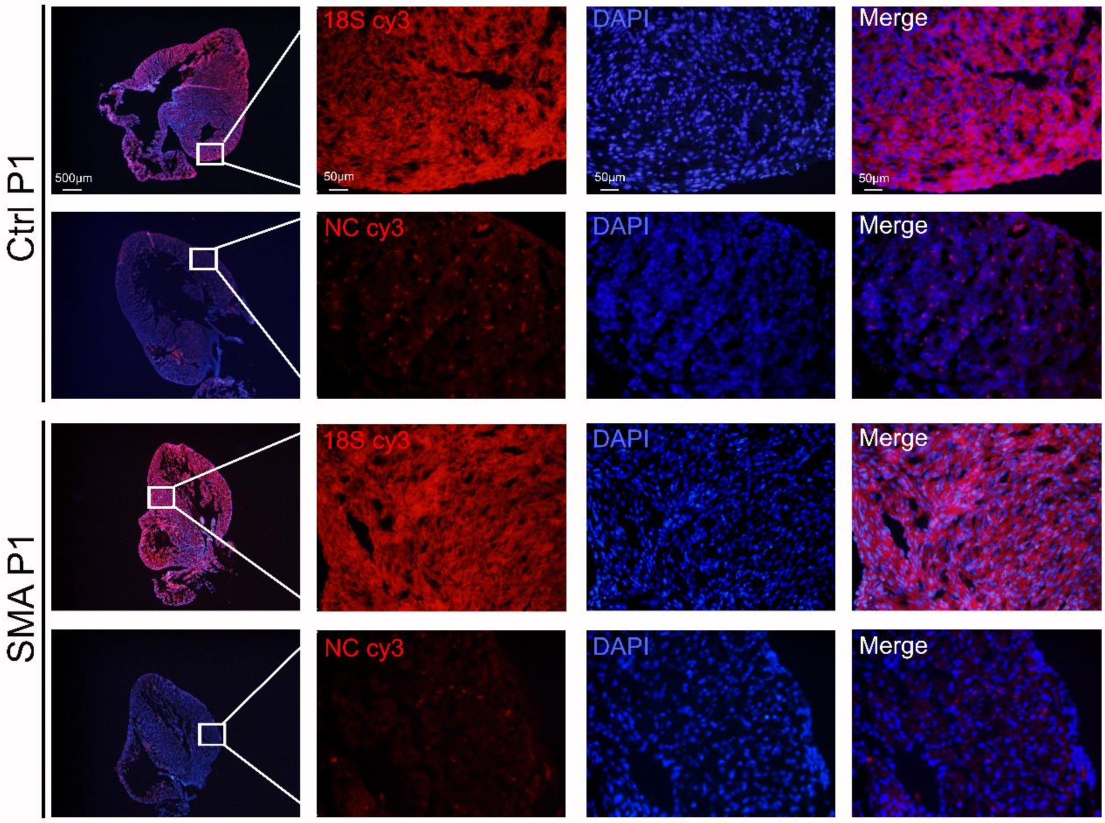

Figure S11. Positive and negative controls of the FISH assay on heart tissue of SMA and heterozygous (Ctrl) mice.

The Cy3-labelled 18S RNA probe (sequence in **Table S4**) and non-related control oligo (NC; sequence in **Table S4**) (red) were used as positive and negative controls, respectively, for FISH analysis of P1 heart samples (n = 3); DAPI was used for nuclear staining (blue). Scale bar = 500μm or 50μm as indicated.

Figure S12

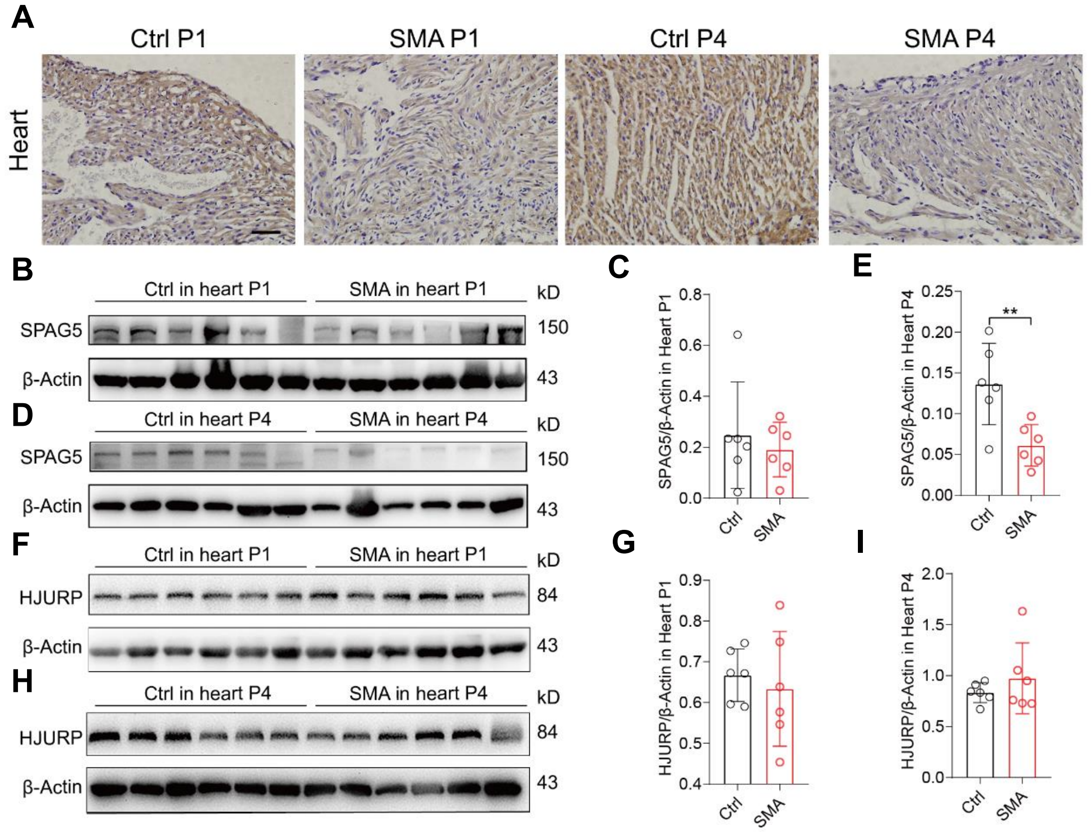

Figure S12. SPAG5 protein levels in heart samples of SMA mice.
(**A**) Immunohistochemistry analysis indicated that expression of SPAG5 were lower in SMA mice (n = 6) than that in heterozygous mice (Ctrl). Scale bar = 50μm as indicated. (**B-E**) Western blot showing that SPAG5 levels were lower in SMA mice at P4 compared to heterozygous mice. β-Actin was used as loading control. Histograms showing quantitation of protein levels of **B** and **D**, respectively. (**F-I**) No significant changes were observed for HJURP. ** $p < 0.01$.

Figure S13

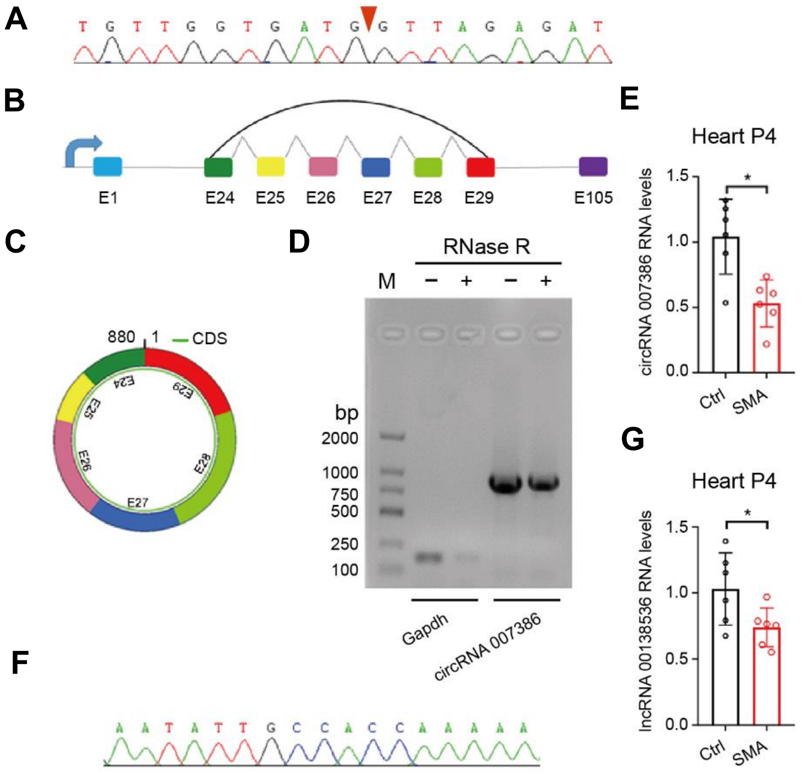

Figure S13. Validation of circRNA007386 and lncRNA00138536 in heart tissue of SMA mice.

(**A**) Cyclization site of circRNA007386 was identified by DNA sequencing (red arrow). (**B-C**) Diagram showing that the circular RNA was generated by cyclization from exon 24 (E24) to E29 of the *Ryr2* gene. (**D**) RT-PCR and agarose gel electrophoresis showed the correct product amplified from circRNA007386 as predicted. Total RNA samples were treated with (+) or without (-) RNase R for 15 min at 37 ºC. The first-strand cDNA was synthesized using random hexamer primers. *Gapdh* was used as control. (**E**) The expression of circRNA007386 was detected by qPCR in heart samples of P4 SMA mice (n = 6) compared to P4 heterozygous controls (Ctrl, n = 6). * $p < 0.05$. (**F**) lncRNA00138536 was identified by DNA sequencing. (**G**) Expression levels of the lncRNA00138536 were lower in P4 heart tissue of SMA mice than control heterozygous mice as detected by qRT-PCR. * $p < 0.05$.

Figure S14

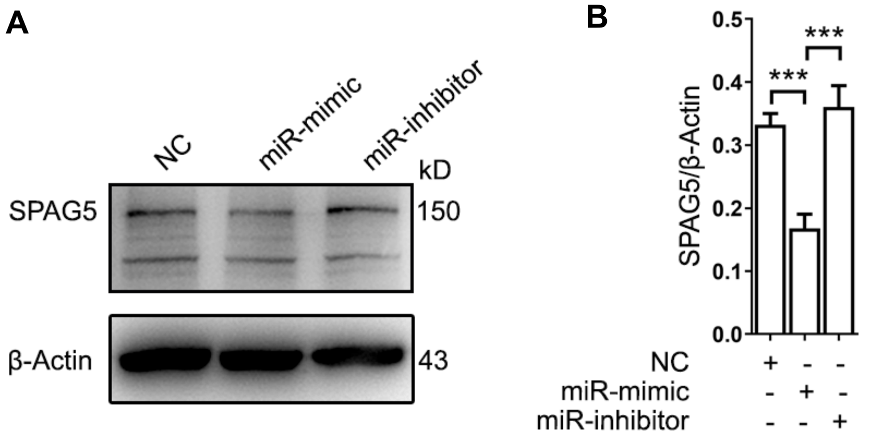

Figure S14. The effect of miR-34a-5p on SPAG5 in C2C12 cells.
(**A**) Western blotting showing protein level changes in C2C12 cells after transfection with 50 nM miR-mimic or 50 nM miR-inhibitor compared 50 nM control NC-oligo (NC). (**B**) Histogram showing protein level decrease after treatment of the mimic and increase after treatment of the inhibitor. β-Actin was used as loading control. *** $p < 0.001$, $n = 3$.

Figure S15

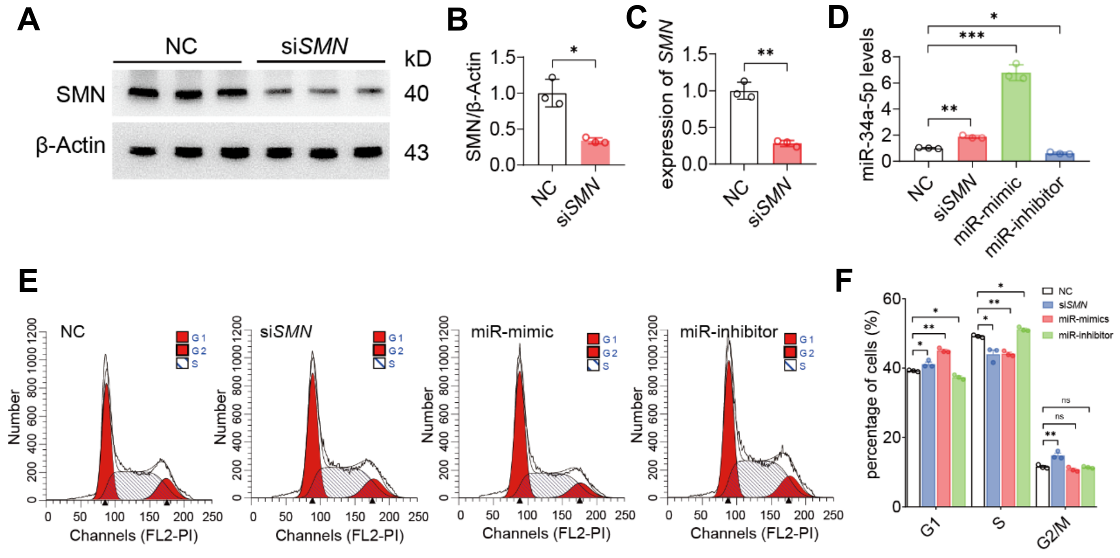

Figure S15. The effects of SMN knockdown, miR-mimic, and miR-inhibitor on cell cycle progression of HEK293T cells.

(**A-C**) Detection of SMN expression in HEK293T cells transfected with si*SMN* or non-related NC-oligo using Western blotting (with β-Actin as loading control) and qRT-PCR. *SMN* represents a mixture of both *SMN1* and *SMN2* transcripts. (**D**) Detection of miR-34a-5p in HEK293T cells transfected with si*SMN,* miR-mimic, miR-inhibitor, or NC-oligo using qRT-PCR. (**E**) Flow cytometry analysis of 293T cells treated with si*SMN,* miR-mimic, miR-inhibitor, or NC-oligo. si*SMN* and miR-mimic treated cells were apparently arrested in G1 phase. (**F**) Quantitation of cells in each phase is shown on right. For all samples, n = 3, * $p < 0.05$, ** $p < 0.01$, *** $p < 0.001$.

Figure S16

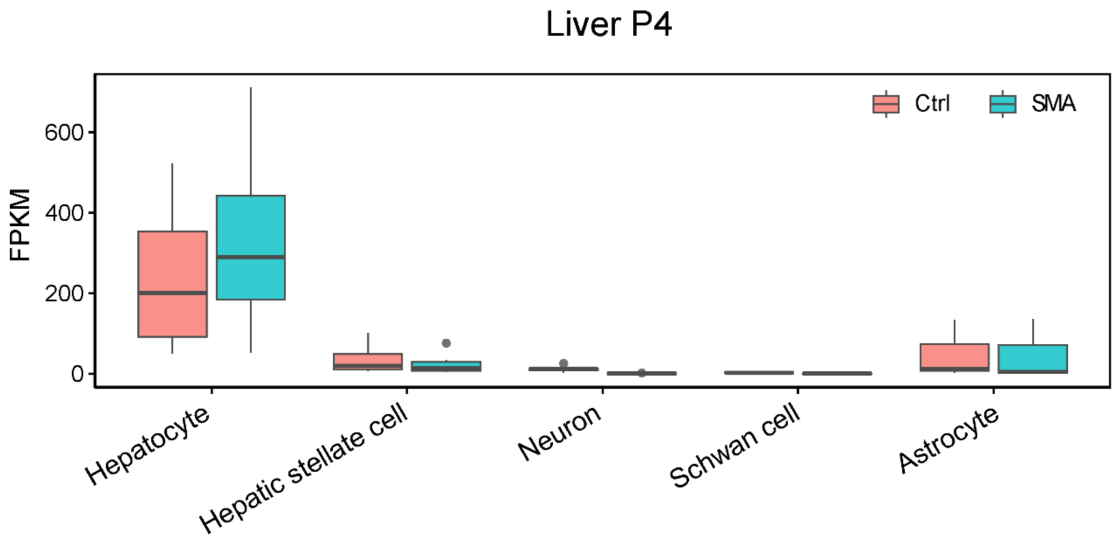

Figure S16. Expression analysis of specific gene markers for different cell types in the P4 liver RNA-seq data.

A subset of gene markers for each cell type were analyzed. The identity of the source as mouse liver was confirmed.

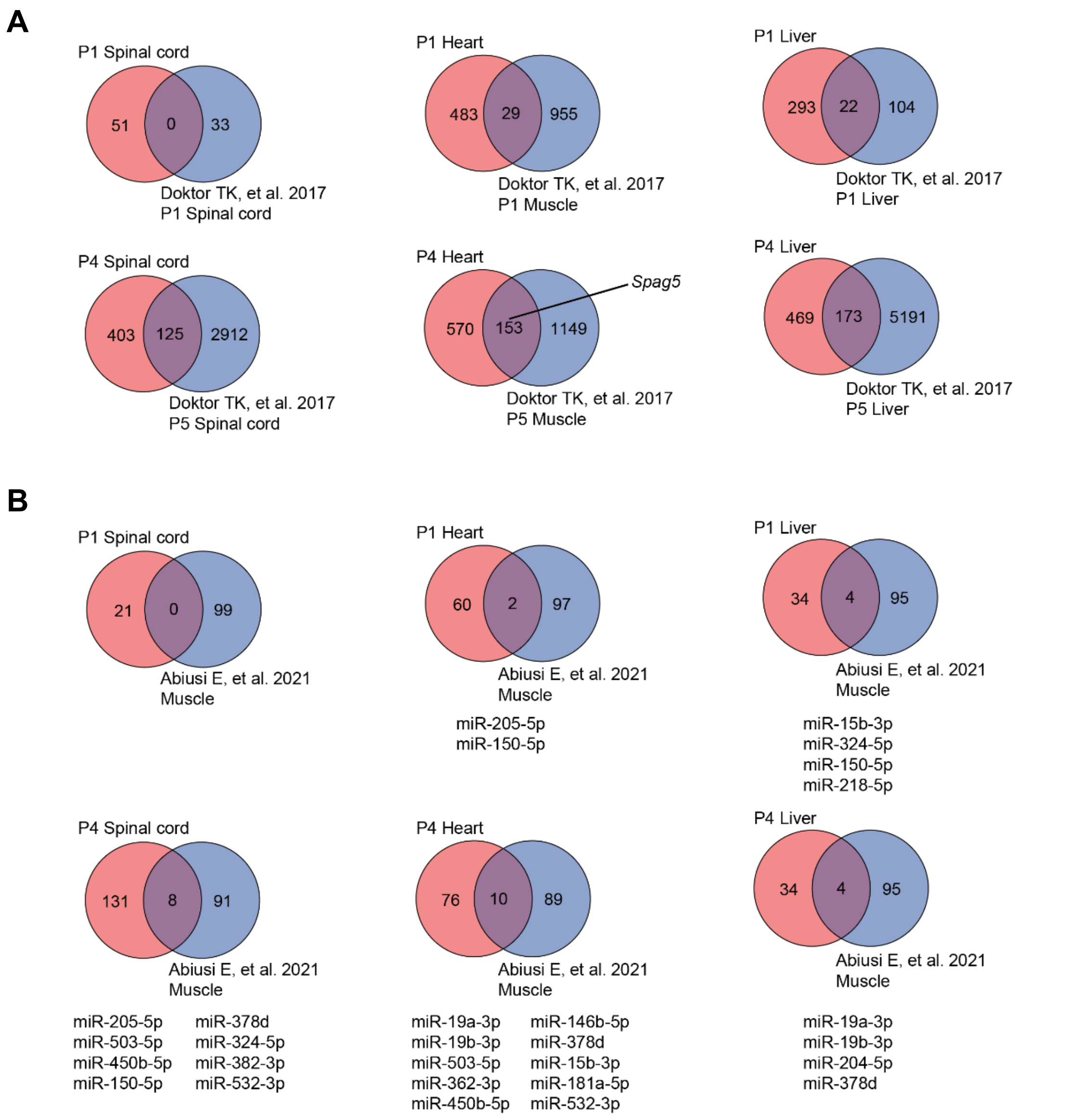

Figure S17. Comparative analysis of DEGs between the present study and two previous studies.

(**A**) Venn diagrams show shared DE-mRNAs between this study (orange, q value < 0.05 and fold-changes > 2) and the one by Doktor TK, et al. (blue, adjusted p value < 0.1) using the same mouse model. Note, Doktor TK, et al. examined spinal cord, liver and skeletal muscle but no heart, and their sampling time points were P1 and P5, so here heart was compared to skeletal muscle, and P4 to P5. (**B**) Venn diagrams show shared DE-miRNAs between this study (orange, p value < 0.05 and fold-changes > 2) and the one by Abiusi E et al. using SMA patients' muscle samples (blue, FDR < 0.05). For the two previous studies, see references #21 and #35 in the manuscript.

Table S1. List of DEGs in all ceRNETs.

Table S2. List of DEGs in miR-34a networks.

Table S3. The FPKM of specific markers for different cell types in the liver at P4.

Cell type	Marker		Ctrl	SMA
Hepatocyte	*ALB*	*Albumin*	NA	NA
	HAMP	*Hepcidin Antimicrobial Peptide*	522.704	710.887
	ARG1	*Arginase 1*	105.227	227.348
	PCK1	*Phosphoenolpyruvate Carboxykinase 1*	296.995	352.808
	AFP	*Alpha Fetoprotein*	NA	NA
	BCHE	*Butyrylcholinesterase*	49.9527	52.6371
Hepatic stellate cell	*ACTA2*	*Actin Alpha 2*	8.90057	5.48844
	COL1A1	*Collagen Type I Alpha 1 Chain*	13.3493	9.04922
	TAGLN	*Transgelin*	6.5482	3.66155
	COL1A2	*Collagen Type I Alpha 2 Chain*	25.3216	17.7321
	COL3A1	*Collagen Type III Alpha 1 Chain*	56.8307	33.3901
	SPARC	*Secreted Protein Acidic And Cysteine Rich*	101.796	75.755
Neuron	*RBFOX3*	*RNA Binding Fox-1 Homolog 3*	2.17763	0.135221
	TAU	*Microtubule Associated Protein Tau*	NA	NA
	NEFL	*Neurofilament Light Chain*	12.5567	0.093592
	MAPT	*Microtubule Associated Protein Tau*	12.6414	0.142636
	MAP2	*Microtubule Associated Protein 2*	25.1072	1.53473
	ENO2	*Enolase 2*	8.36235	0.517755
Schwan cell	*SOX10*	*SRY-Box Transcription Factor 10*	5.97526	0.245251
	S100B	*S100 Calcium Binding Protein B*	0.870555	0.038472
Astrocyte	*S100B*	*S100 Calcium Binding Protein B*	1.84123	0.076897
	GFAP	*Glial Fibrillary Acidic Protein*	1.84123	0.076897
	NDRG2	*NDRG Family Member 2*	10.9429	4.24871

NA, not available.

Table S4. Information of primers, siRNAs, and oligonucleotides used in the present study.

Primers for miRNAs expression and validation	
Name	Sequence (5'-3')
miR-34a-5p (RT)	CCTGTTGTCTCCAGCCACAAAAGAGCACAATATTTCAGGAGACAAC AGGACAACCA
miR-34a-5p (forward primer)	CGGGCTGGCAGTGTCTTAGC
miRNA (reverse primer)	CAGCCACAAAAGAGCACAAT
RNU6 (forward)	CTCGCTTCGGCAGCACATATACT
RNU6 (reverse)	ACGCTTCACGAATTTGCGTGTC
RNA probe information	
NC	Cy3-5´-UGCUUUGCACGGUAACGCCUGUUUU-3´
18S	Cy3-5´-CUUCCUUGGAUGUGGTAGCCGUUUC-3´
miR-34a-5p	Cy3-5´-ACAACCAGCUAAGACACUGCCA-3´

Table S4, continued.

Primers for lncRNAs expression and validation		
Name	Forward sequence (5'-3')	Reverse sequence (5'-3')
lnc00007921	TTGGTCGGTTGGTTGGTAA	GGCTGTGGGGAATGAGATG
lnc00138536	CCCTCAGTGTTTTGATGCC	AGTGTATGTGGGAGTTGGAAGT
lnc00150507	GCCTCTAATTTTGTTCACTGCC	TTCCAAGAATGAAAACCTCTAACC
Primers for circRNAs expression and validation		
Name	Left sequence (5'-3')	Right sequence (5'-3')
circ007386	TCTGAGCTGGCATTCAAGGA	CCCAATGCCAGCAAAGTCTT
circ014460	AAGCCACTTCCTTTGTTCCT	TTGTGACGCGACTGGAGTAT
circ007386seq	GGCGCTGGTACTTTGAATTT	GTGAGCATTTTCAGCCAACT
Primers for mRNAs expression		
Name	Forward sequence (5'-3')	Reverse sequence (5'-3')
Gapdh	CCGTAGACAAAATGGTGAAGGT	CGTGAGTGGAGTCATACTGGAA
Cdca8	ATGGCTCCCAAGAAACGC	GGTCTGTCTGTCGGACTCAAT
Cenpe	TAAAGTCCCGACAAGCATAAC	CTCCACTCTACCTCAGCCAAT
Hjurp	CCTTCCGTGACCTCATCTGTC	GCTGCTTACGCTGTTGCTG
Spag5	CTGAAGTTGGAAAATAGTCGCC	GCTCCTTGTTGCTCTGGGTA
GAPDH	AAGGTGAAGGTCGGAGTCAACG G	CCACTTGATTTTGGAGGGATCTC
SMN1/2	AGCTGTGGCTTCATTTAAGCAT	CAGAACATTTGTCCCCAACTTT

Table S4, continued.

Primers for construction of dual-fluorescence report plasmid	
Name	Sequence (5'-3')
lnc38536Wt (XhoI) F	GCGGCTCGAGTCACCTCTCAGGTCACTTGCC
lnc38536Wt (notI) R	AATGCGGCCGCGTATTTGCTTCCTCCTAAGTCAGTG
lnc8536Mut F	ACCTGAGTGCCAAAACTTGGTGACGGTAGGGCCCAGGAAAGT GACACAGATG
lnc38536Mut R	CAAGTTTTGGCACTCAGGTGACAAGAGGGGAGTCTT
circ7386-182Wt (ASI) F	GCGGGCGATCGCGTTAGAGATGACAACAAGAGACAG
circ7386-649Wt(notI) R	AATGCGGCCGCCATCACCAACATCAAAGTCCTT
circ7386-182Mut F	TGAAGAAAATGAAACTGCGATTCTATTACCAGCTGACCAG
circ7386-182Mut R	GCAGTTTCATTTTCTTCACCTTCTCTTCAGCATG
circ7386-649Mut F	TGGAGCAGGCCAGGCTGCGTTGGTCATCTAGAACTGGGTTC
circ7386-649Mut R	GCAGCCTGGCCTGCTCCAGCCCACCCGCATGTCT
*Spag5-1424*Wt (ASI) F	GCGGGCGATCGCATCTGGCTACCTTGTCCCG
*Spag5-1424*Wt (NOTI) R	AATGCGGCCGCTGTTCCAGTTGGCTGATGC
*Spag5-1424*Mut F	GCTCTCCTTGTGGGGTCAGATTCTGTGCTAAACATCTTCAGGA
*Spag5-1424*Mut R	TGACCCCACAAGGAGAGCTGTCAGTCTGTGTACT
*Spag5-3285*Wt (AsiSI) F	GCGGGCGATCGCTGCAGAGGGAAATCTGTGAAC
*Spag5-3285*Wt (NOTI) R	AATGCGGCCGCTCCAGGATGTGCCTATGGC
*Spag5-3285*Mut F	AGGCCAGCTAGATCCCAGGACGGTGCTGATGGCTACTAAC
*Spag5-3285*Mut R	CTGGGATCTAGCTGGCCTTCCAGGGCTTCCTGGA
*Spag5-3675*Wt (XhoI) F	GCGGCTCGAGAACAGCTGATGGACAAGTATCTGAG
*Spag5-3675*Wt (notI) R	AATGCGGCCGCAATAACATAAAACATGGTCGGCTC
*Spag5-3675*Mut F	ATTTGGGAACCTAACACAGTGTTGAGTCGAAAAACCCTGAAA AAT
*Spag5-3675*Mut R	CTGTGTTAGGTTCCCAAATTCCTGGGATTTGGCAGC

Table S4, continued.

Primers for construction of overexpression plasmid	
Name	Sequence (5'-3')
circ007386OE F	CGGAATTCTGAAATATGCTATCTTACAGGTTAGAGATGACAACAAGAGACAG
circ007386OE R	CGGGATCCTCAAGAAAAAATATATTCACCATCACCAACATCAAAGTCCTT
lnc00138536OEkpn1 F	ACGGTACCTCACCTCTCAGGTCACTTGCC
lnc00138536OEBamHI R	ATGGATCCGTATTTGCTTCCTCCTAAGTCAGTG
Spag5 3’UTRxbaI F	CGGTCTAGAAACAGCTGATGGACAAGTATCTGAG
Spag5 3’UTRBamHI R	AATGGATCCAATAACATAAAACATGGTCGGCTC
*Spag5*cdsxbaI F	ACTCTAGAATGTGGAGGGTGAAAACACTGAA
*Spag5*cdskpn1 R	ACGGTACC TTAGCTCAGAAATTCTAGCAATCCT

siRNAs and oligonucleotides		
Name	Sense sequence (5'-3')	Antisense sequence (5'-3')
Negative control (NC)	UUCUCCGAACGUGUCACGUTT	ACGUGACACGUUCGGAGAATT
si*Smn*	GACCUGUGAAGUAGCUAAUTT	AUUAGCUACUUCACAGGUCTT
si*SMN*	CUUGAUGAUGCUGAUGCUUU	AAAGCAUCAGCAUCAUCAAG
miR-34a-5p mimic	UGGCAGUGUCUUAGCUGGUUGU	AACCAGCUAAGACACUGCCAUU
miR-34a-5p inhibitor	ACAACCAGCUAAGACACUGCCA	

Table S4, continued.

Sequences of decoy MRE oligonucleotides for target genes with modified phosphorothioate backbone	
Spag5 3’UTR WT	ACAACACAGCAAAAACCCUGAAA
Spag5 3’UTR Mut	UGUUGAGUCGAAAAACCCUGAAA
lnc00138536 WT	AGUGCCAAAACUUGCACUGCCA
lnc00138536 Mut	AGUGCCAAAACUUGGUGACGGU
circ007386 WT1	GAAACUGCCUAAGAAUUACCA
circ007386 WT2	CCAACCAGAUCUAGAACUGGGU
circ007386 Mut1	GAAACUGCGAUUCUAUUACCA
circ007386 Mut2	CGUUGGUCAUCUAGAACUGGGU

Figure S1

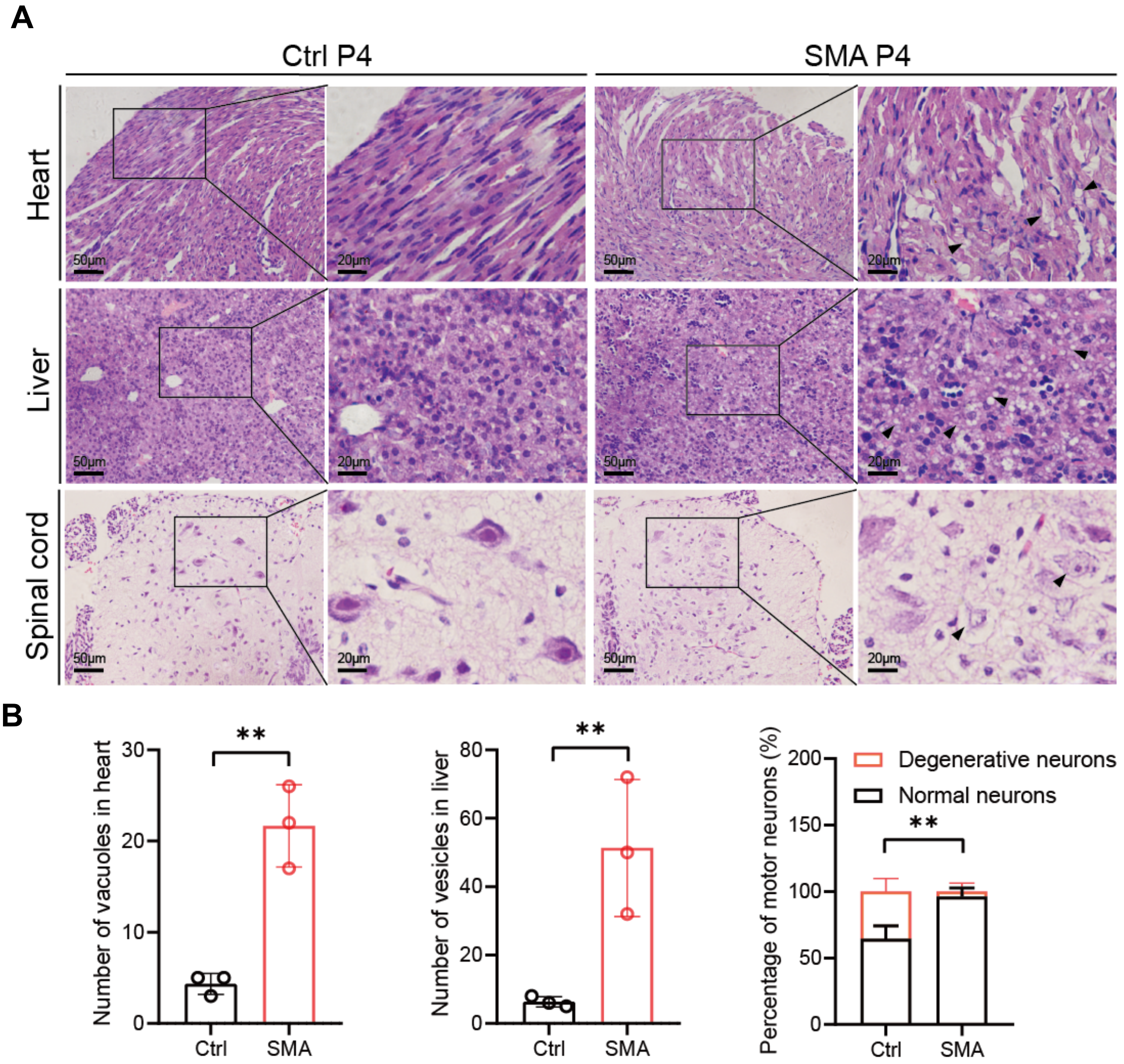

Figure S1. The hematoxylin and eosin (H&E) staining of heart, liver, and spinal cord tissues of SMA and heterozygous (Ctrl) mice.

(**A**) Tissues were collected from SMA mice (n = 3) at P4 and fixed with 4% formaldehyde at 4 ºC overnight. After washing in 0.01 M phosphate-buffered saline, tissues were embedded in paraffin blocks and 4-μm–thick sections were cut for H&E staining. The number of cells in heart tissue of SMA mice was relatively lower than that in heterozygous mice and tissue vacuolization (arrows) was observed. In the liver, a large number of lightly stained vesicles (arrows) were detected. As for the spinal cord, considerable motor neurons in the anterior horn displayed intranuclear vacuolization (arrows). Scale bar = 50 or 20 μm as indicated. (**B**) Quantitation of the number of vacuoles in cardiomyocytes per high-power field, the number of vesicles in hepatocytes, the percentage of degenerative neurons per high-power field. ** $p < 0.01$ (n = 3).

Figure S2

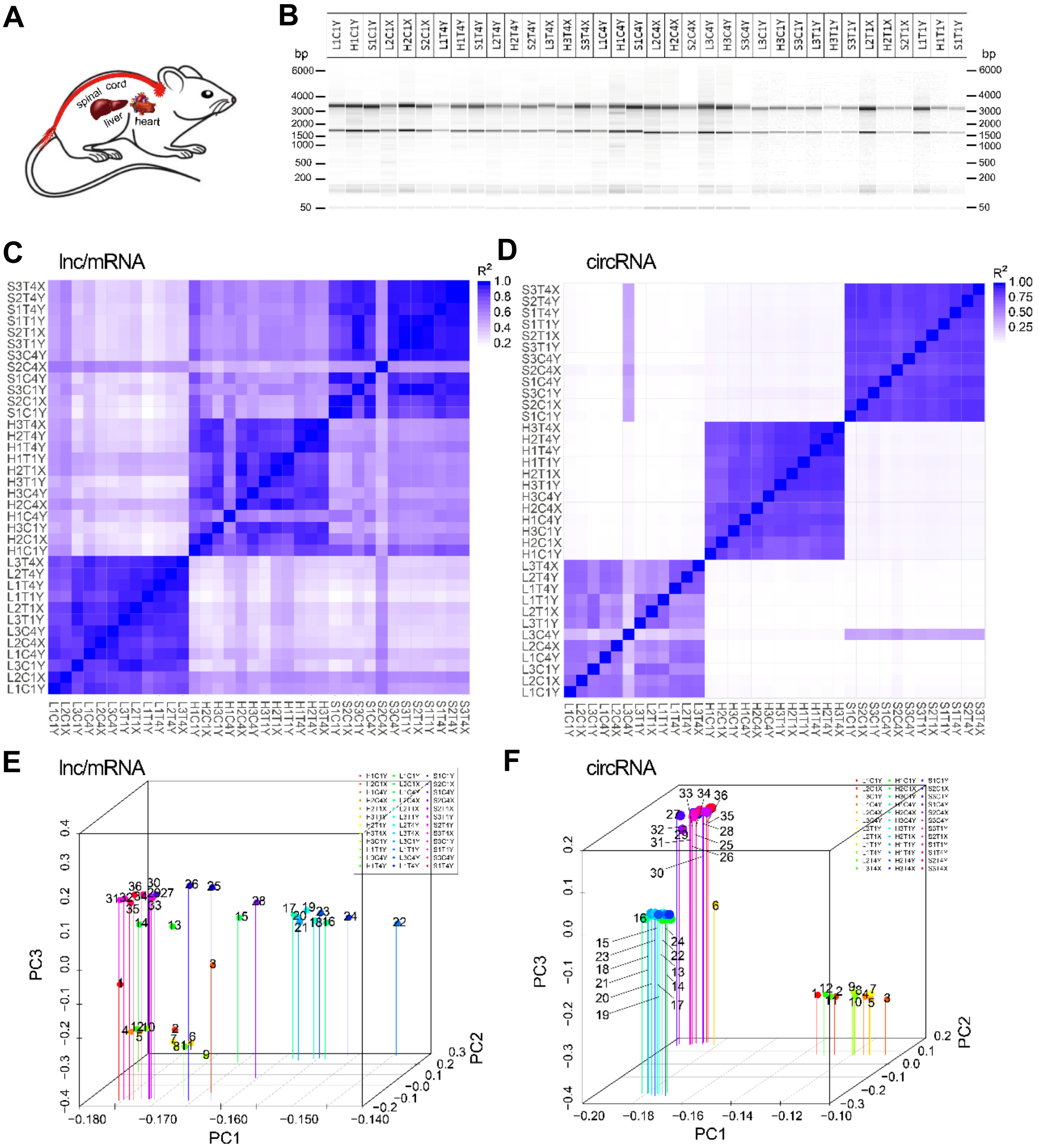
A
spinal cord
liver
heart
B
C
lnc/mRNA
D
circRNA
E
lnc/mRNA
F
circRNA
PC1
PC2
PC3

Figure S2. Quality analysis of the total RNA samples used in the present study. (**A**) The schematic diagram of mouse tissues used in the present study. (**B**) Capillary gel electrophoresis was performed to assess the quality of total RNA samples using Labchip GX (PerkinElmer, MA, USA). Each lane represents a sample, named as tissue-mouse #-mouse type-age-sex with L stands for liver, H heart, S spinal cord, C control mice, T SMA mice, Y male, and X female. For example, L1T4Y represents liver tissue collected from #1 SMA mouse at P4 that was male. (**C** and **D**) The Pearson correlation analysis of lncRNAs, mRNAs and circRNAs in mouse samples. (**E** and **F**) Three-dimension principal component analysis (PCA) plots with 36 balls (for all 36 tissue samples) shown. The principal components were converted from original variable data using orthogonal transformation to achieve data dimensionality reduction. The position of each ball in PCA plots represents the value of the sample on each principal component, and different colors represent different samples. PC1, PC2, and PC3 represent different calculation methods for gene expression profiles in matrix form, respectively.

Figure S3

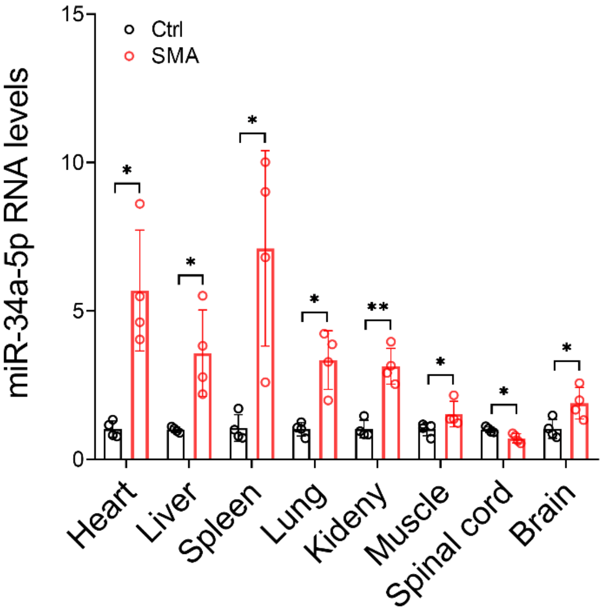

Figure S3. Expressions of miR-34a-5p in eight tissues of SMA mice.
Examination of miR-34a-5p expression levels in eight tissues of SMA mice, including heart, liver, spleen, lung, kidney, muscle, spinal cord and brain. * $p < 0.05$, ** $p < 0.01$, $n = 4$.

Figure S4

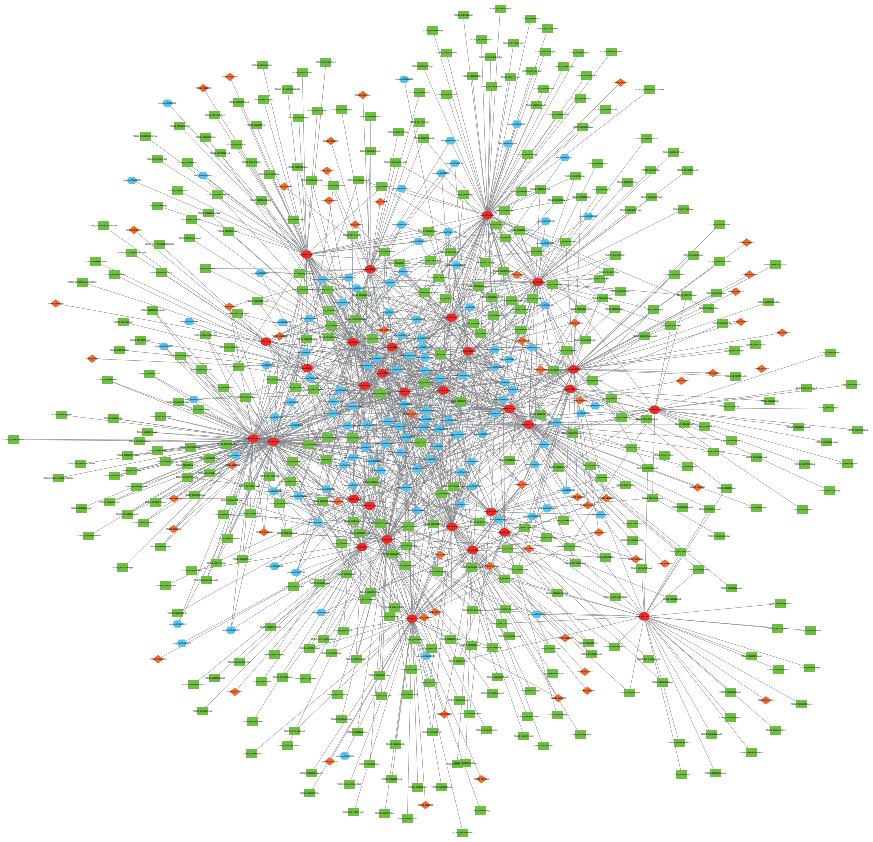

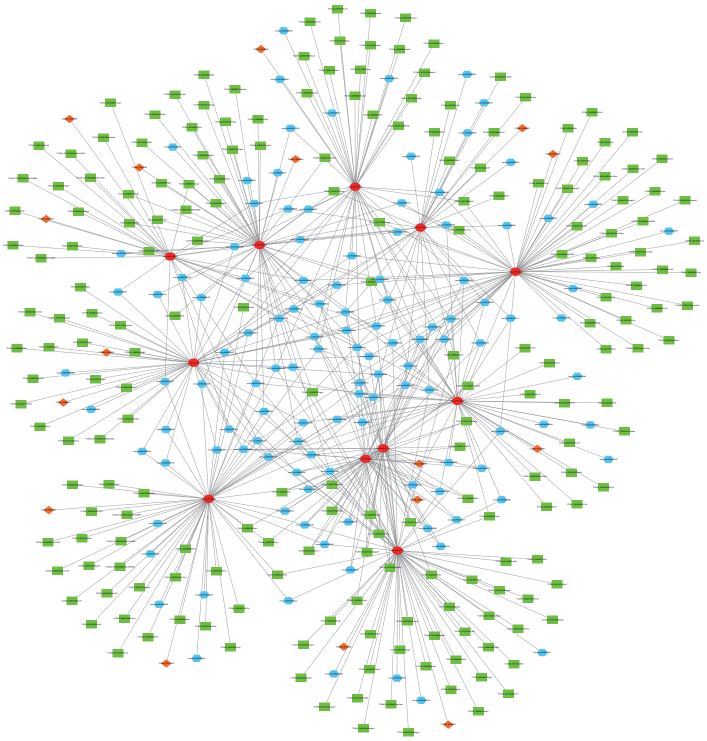

Figure S4. lncRNA-circRNA-miRNA-mRNA networks for P1 heart samples of SMA mice.

The left network shows decreased miRNAs and corresponding increase of their target ceRNAs, while the right one shows increased miRNAs and corresponding decrease of their target ceRNAs. All ceRNAs detected in P1 heart tissues are shown in **Figure S10** and **Table S1**. Rectangles represent mRNAs, diamonds represent lncRNAs, hexagons represent circRNAs, and ellipses represent miRNAs.

Figure S5

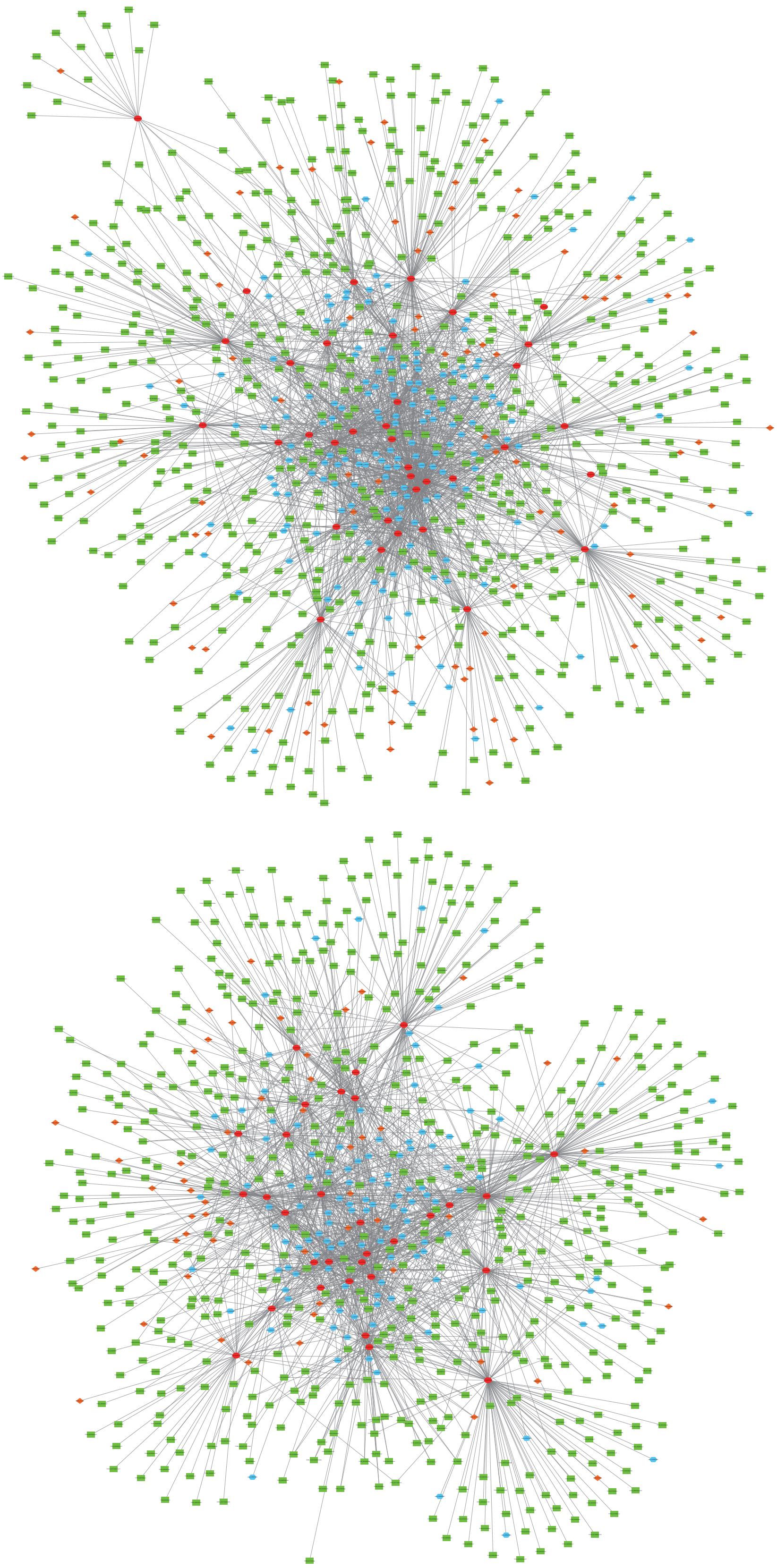

Figure S5. lncRNA-circRNA-miRNA-mRNA networks for P4 heart samples of SMA mice.

The left network shows decreased miRNAs and corresponding increase of their target ceRNAs, while the right one shows increased miRNAs and corresponding decrease of their target ceRNAs. All ceRNAs detected in P4 heart tissues are shown in **Figure S10** and **Table S1**. Rectangles represent mRNAs, diamonds represent lncRNAs, hexagons represent circRNAs, and ellipses represent miRNAs.

Figure S6

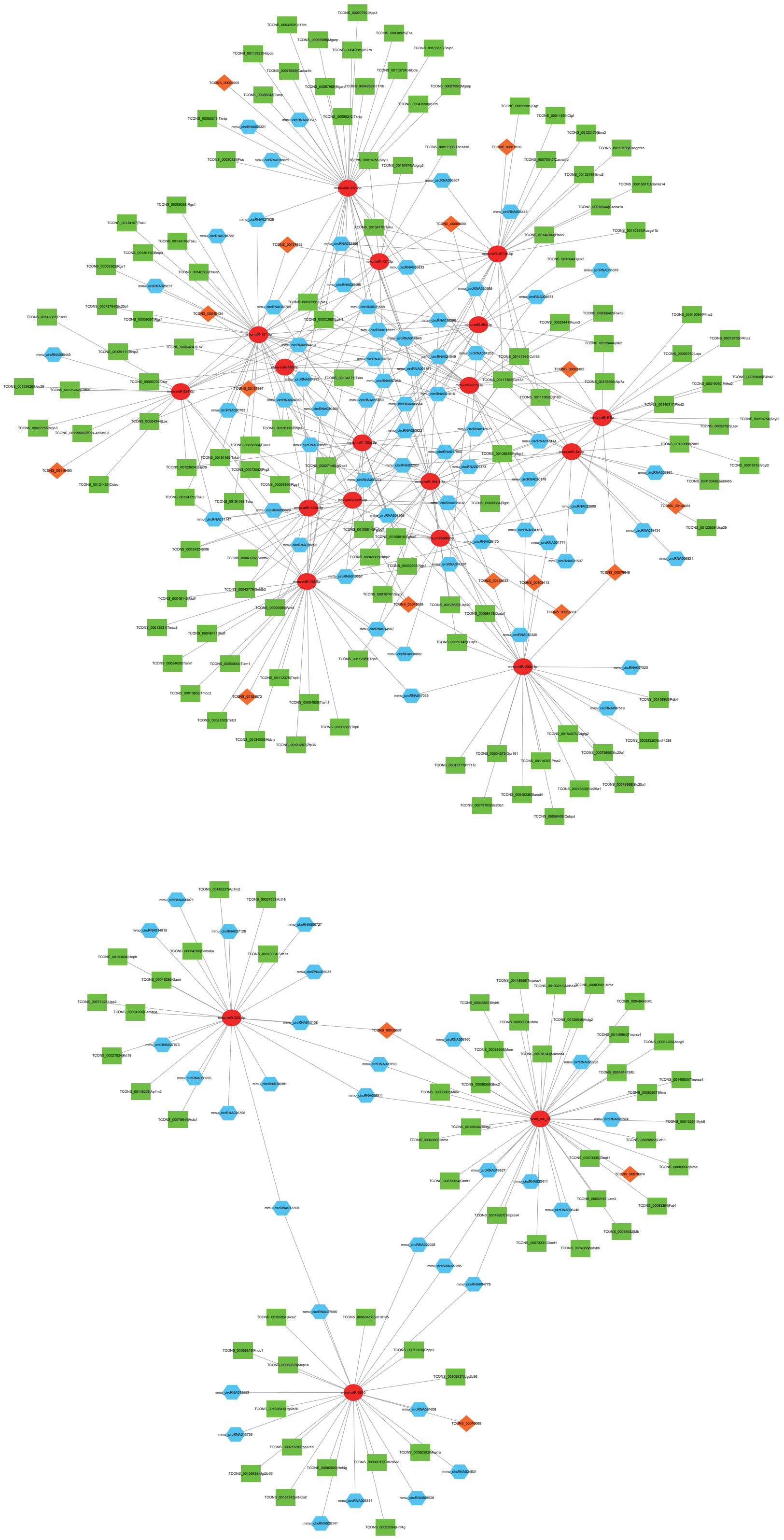

Figure S6. lncRNA-circRNA-miRNA-mRNA networks for P1 liver samples of SMA mice.

The left network shows decreased miRNAs and corresponding increase of their target ceRNAs, while the right one shows increased miRNAs and corresponding decrease of their target ceRNAs. All ceRNAs detected in P1 liver tissues are shown in **Figure S10** and **Table S1**. Rectangles represent mRNAs, diamonds represent lncRNAs, hexagons represent circRNAs, and ellipses represent miRNAs.

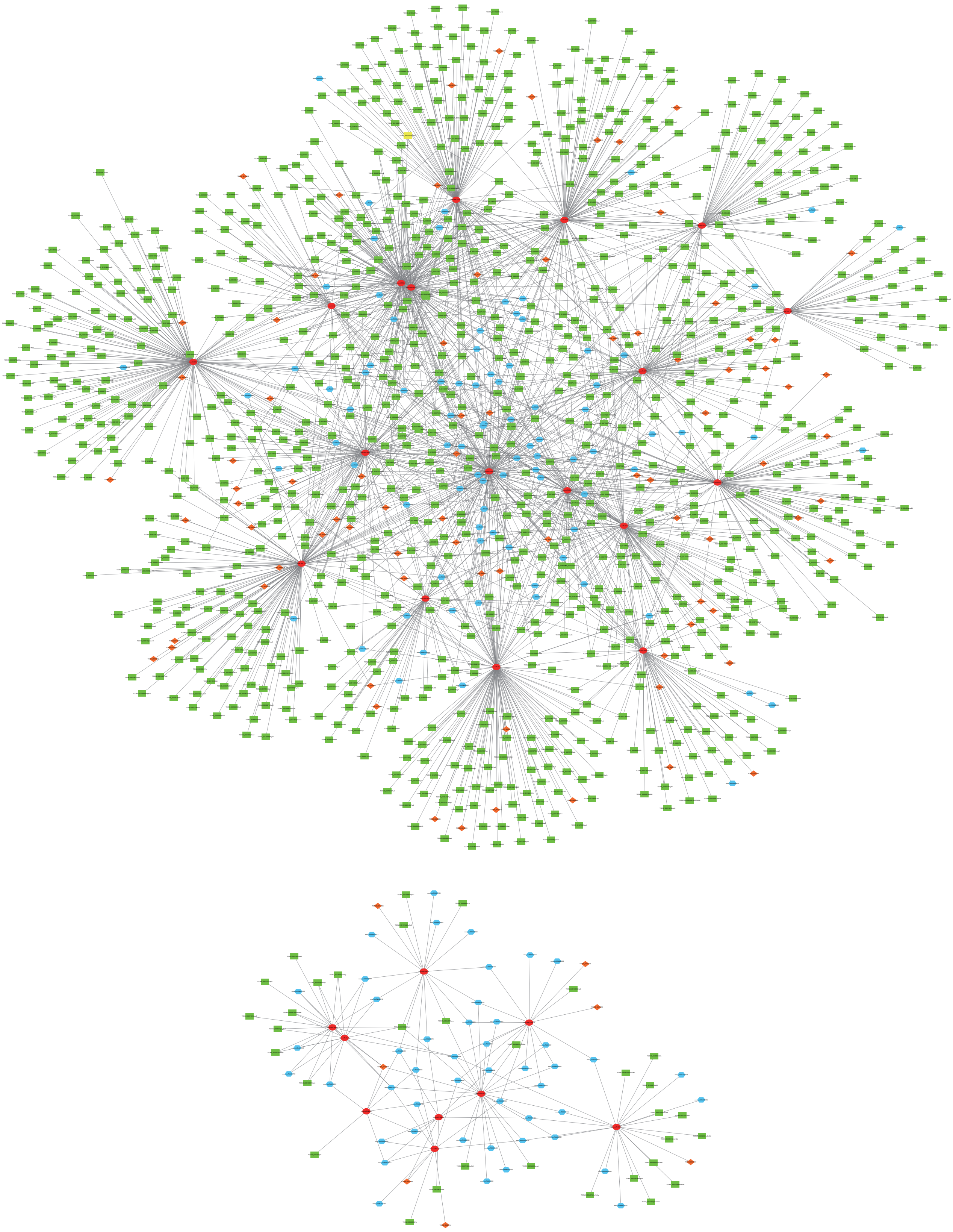

Figure S7. lncRNA-circRNA-miRNA-mRNA networks for P4 liver samples of SMA mice.

The left network shows decreased miRNAs and corresponding increase of their target ceRNAs, while the right one shows increased miRNAs and corresponding decrease of their target ceRNAs. All ceRNAs detected in P4 liver tissues are shown in **Figure S10** and **Table S1**. Rectangles represent mRNAs, diamonds represent lncRNAs, hexagons represent circRNAs, and ellipses represent miRNAs.

Figure S8

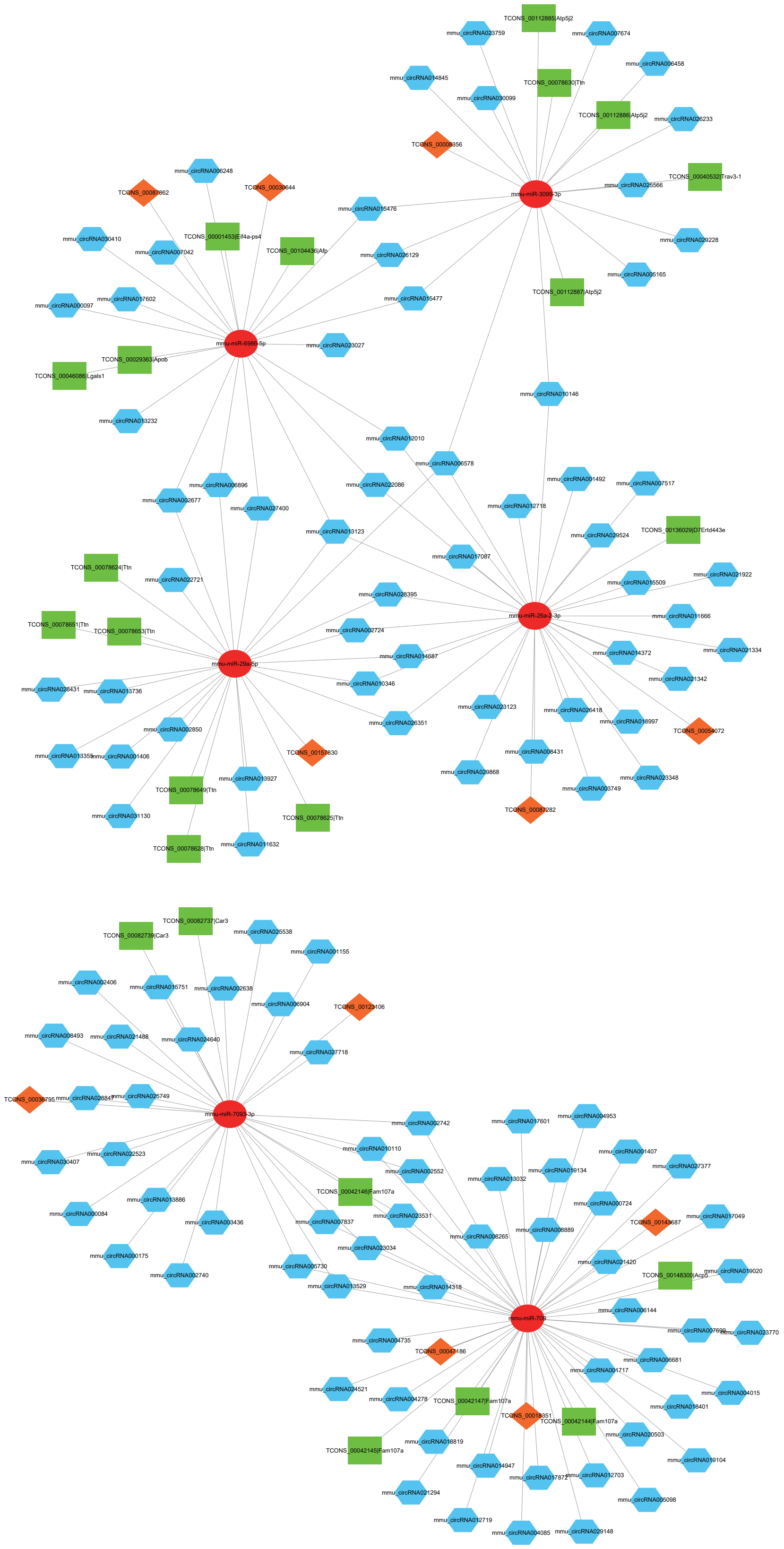

Figure S8. lncRNA-circRNA-miRNA-mRNA networks for P1 spinal cord samples of SMA mice.

The left network shows decreased miRNAs and corresponding increase of their target ceRNAs, while the right one shows increased miRNAs and corresponding decrease of their target ceRNAs. All ceRNAs detected in P1 spinal cord tissues are shown in **Figure S10** and **Table S1**. Rectangles represent mRNAs, diamonds represent lncRNAs, hexagons represent circRNAs, and ellipses represent miRNAs.

Figure S9

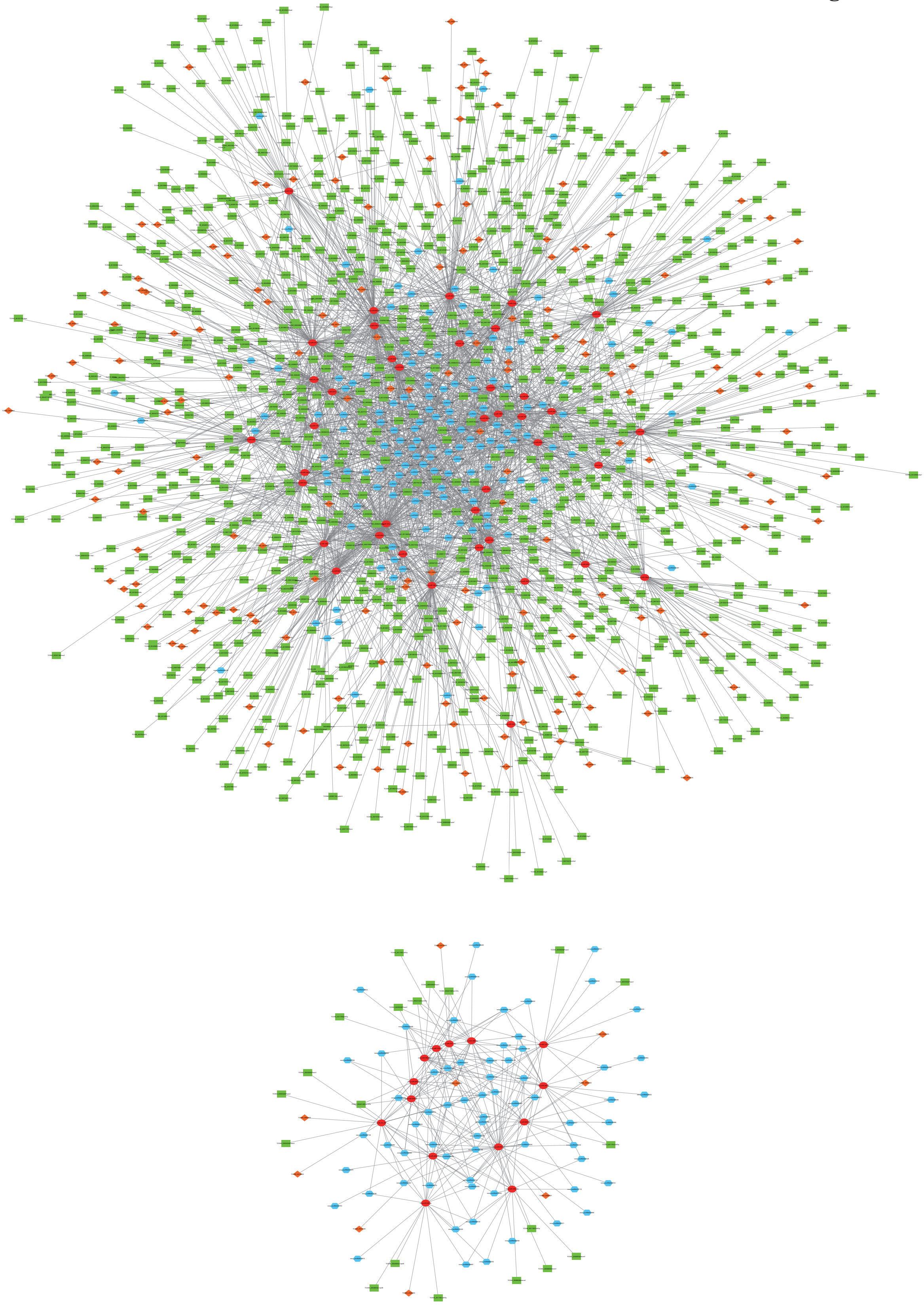

Figure S9. lncRNA-circRNA-miRNA-mRNA networks for P4 spinal cord samples of SMA mice.

The left network shows decreased miRNAs and corresponding increase of their target ceRNAs, while the right one shows increased miRNAs and corresponding decrease of their target ceRNAs. All ceRNAs detected in P4 spinal cord tissues are shown in **Figure S10** and **Table S1**. Rectangles represent mRNAs, diamonds represent lncRNAs, hexagons represent circRNAs, and ellipses represent miRNAs.

Figure S10

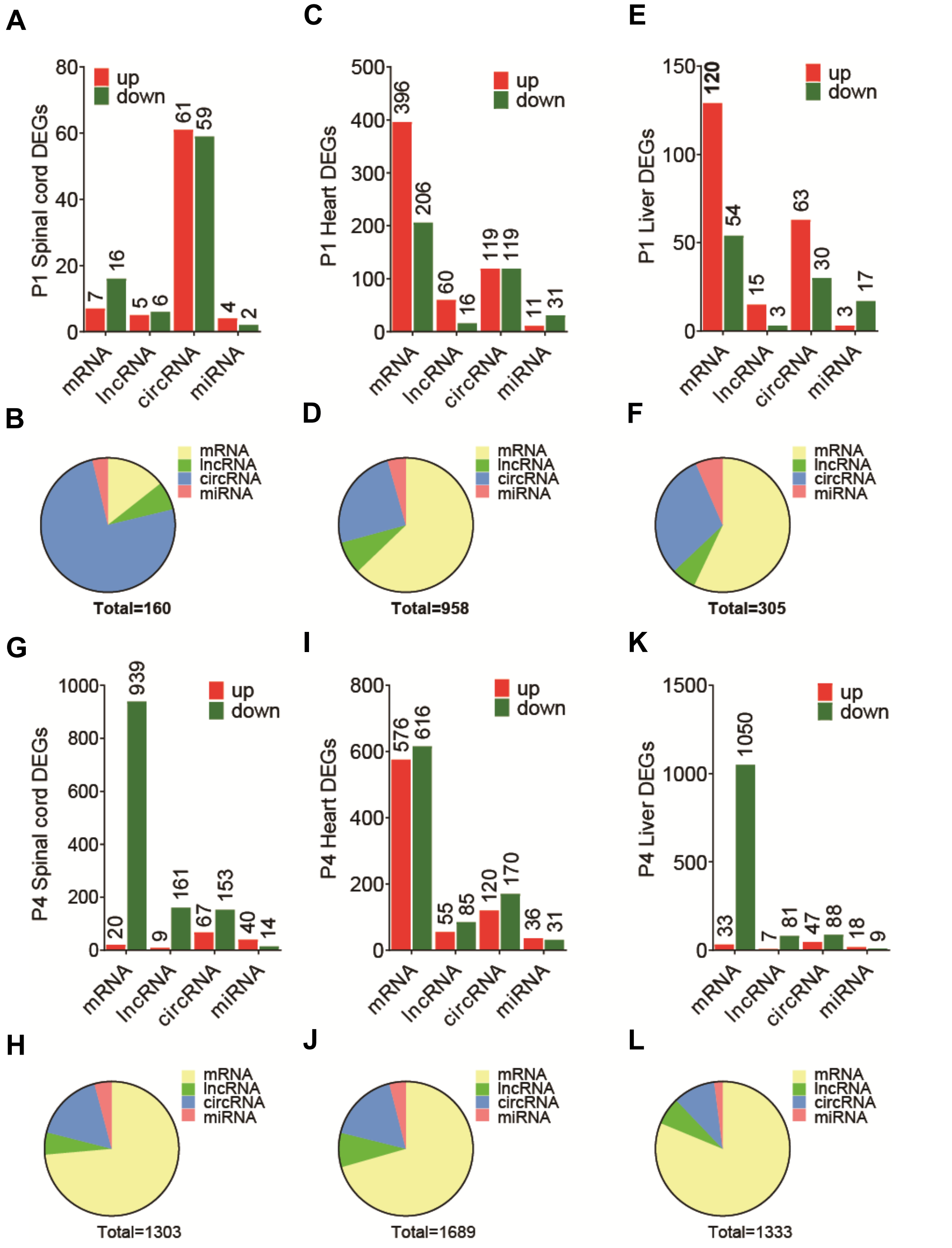

Figure S10. The numbers of differentially expressed ceRNAs in heart, liver, and spinal cord tissues of SMA mice.

Histograms and sector diagrams showing the numbers of differentially expressed ceRNAs of P1 and P4 spinal cord (**A**, **B**, **G**, and **H**), heart (**C**, **D**, **I**, and **J**), and liver (**E**, **F**, **K**, and **L**) samples.

Figure S11

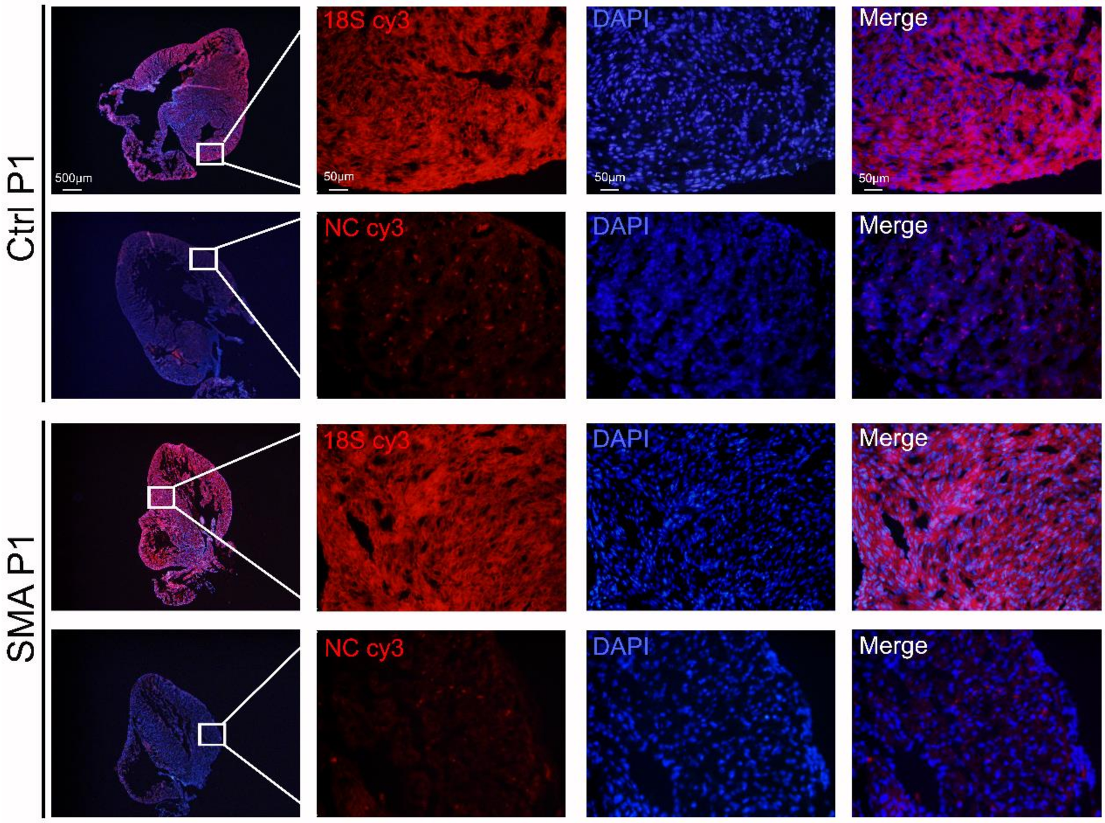

Figure S11. Positive and negative controls of the FISH assay on heart tissue of SMA and heterozygous (Ctrl) mice.

The Cy3-labelled 18S RNA probe (sequence in **Table S4**) and non-related control oligo (NC; sequence in **Table S4**) (red) were used as positive and negative controls, respectively, for FISH analysis of P1 heart samples (n = 3); DAPI was used for nuclear staining (blue). Scale bar = 500μm or 50μm as indicated.

Figure S12

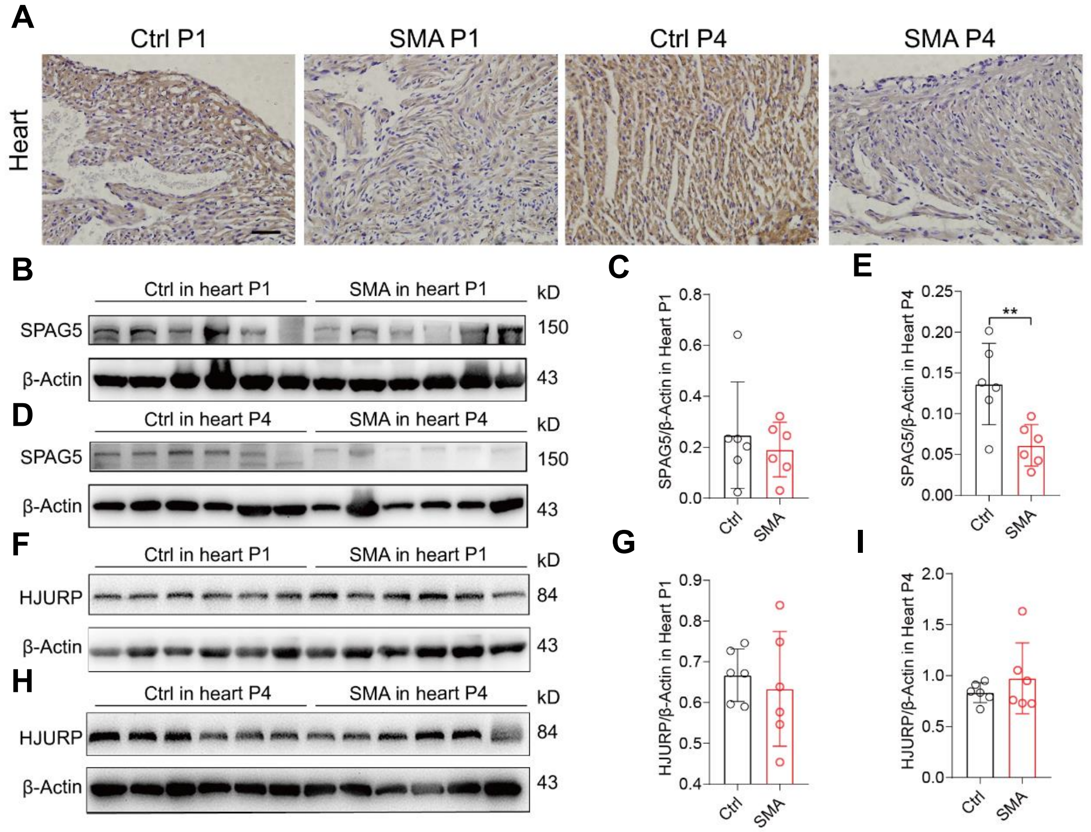

Figure S12. SPAG5 protein levels in heart samples of SMA mice.

(**A**) Immunohistochemistry analysis indicated that expression of SPAG5 were lower in SMA mice (n = 6) than that in heterozygous mice (Ctrl). Scale bar = 50μm as indicated. (**B-E**) Western blot showing that SPAG5 levels were lower in SMA mice at P4 compared to heterozygous mice. β-Actin was used as loading control. Histograms showing quantitation of protein levels of **B** and **D**, respectively. (**F-I**) No significant changes were observed for HJURP. ** $p < 0.01$.

Figure S13

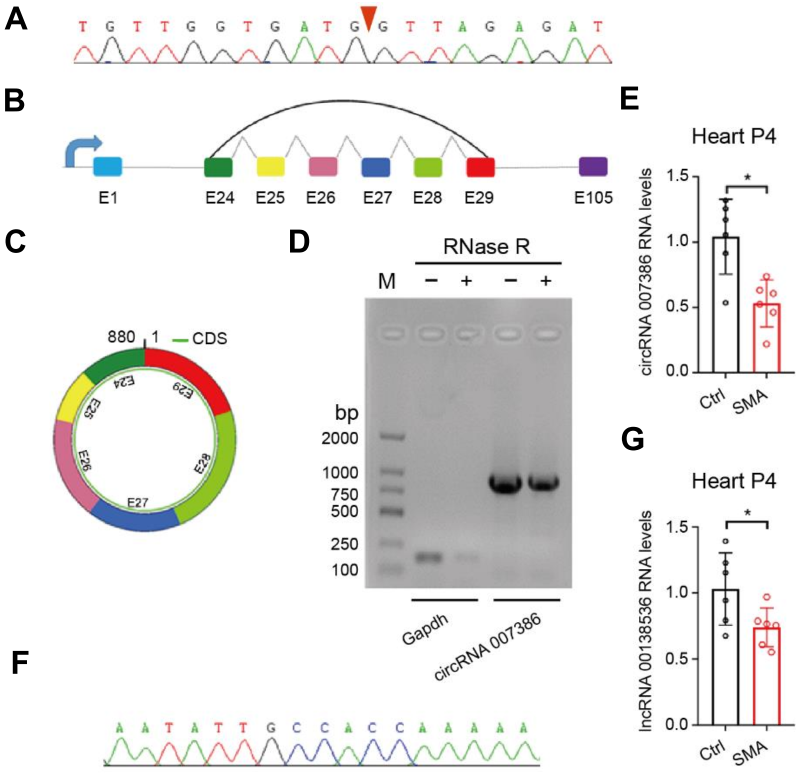

Figure S13. Validation of circRNA007386 and lncRNA00138536 in heart tissue of SMA mice.

(**A**) Cyclization site of circRNA007386 was identified by DNA sequencing (red arrow). (**B-C**) Diagram showing that the circular RNA was generated by cyclization from exon 24 (E24) to E29 of the *Ryr2* gene. (**D**) RT-PCR and agarose gel electrophoresis showed the correct product amplified from circRNA007386 as predicted. Total RNA samples were treated with (+) or without (-) RNase R for 15 min at 37 °C. The first-strand cDNA was synthesized using random hexamer primers. *Gapdh* was used as control. (**E**) The expression of circRNA007386 was detected by qPCR in heart samples of P4 SMA mice (n = 6) compared to P4 heterozygous controls (Ctrl, n = 6). * $p < 0.05$. (**F**) lncRNA00138536 was identified by DNA sequencing. (**G**) Expression levels of the lncRNA00138536 were lower in P4 heart tissue of SMA mice than control heterozygous mice as detected by qRT-PCR. * $p < 0.05$.

Figure S14

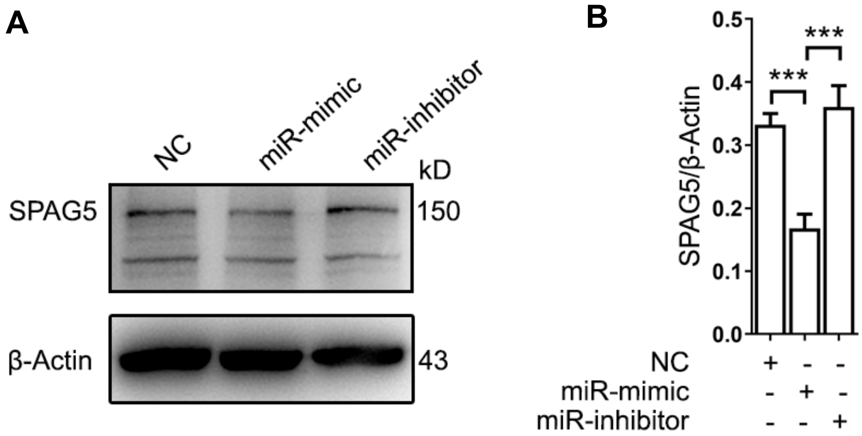

Figure S14. The effect of miR-34a-5p on SPAG5 in C2C12 cells.

(**A**) Western blotting showing protein level changes in C2C12 cells after transfection with 50 nM miR-mimic or 50 nM miR-inhibitor compared 50 nM control NC-oligo (NC). (**B**) Histogram showing protein level decrease after treatment of the mimic and increase after treatment of the inhibitor. β-Actin was used as loading control. *** $p < 0.001$, $n = 3$.

Figure S15

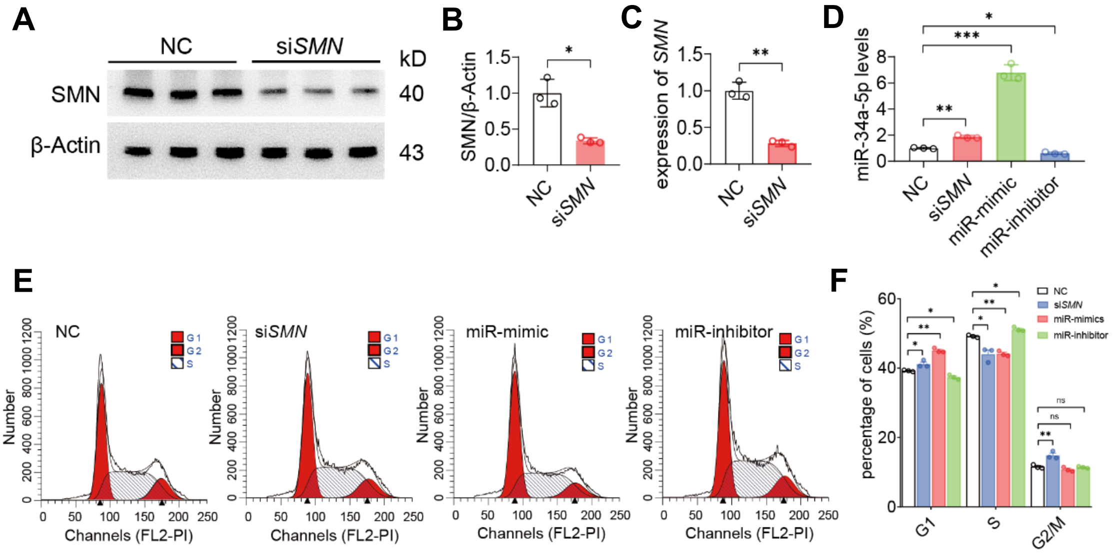

Figure S15. The effects of SMN knockdown, miR-mimic, and miR-inhibitor on cell cycle progression of HEK293T cells.

(**A-C**) Detection of SMN expression in HEK293T cells transfected with si*SMN* or non-related NC-oligo using Western blotting (with β-Actin as loading control) and qRT-PCR. *SMN* represents a mixture of both *SMN1* and *SMN2* transcripts. (**D**) Detection of miR-34a-5p in HEK293T cells transfected with si*SMN,* miR-mimic, miR-inhibitor, or NC-oligo using qRT-PCR. (**E**) Flow cytometry analysis of 293T cells treated with si*SMN,* miR-mimic, miR-inhibitor, or NC-oligo. si*SMN* and miR-mimic treated cells were apparently arrested in G1 phase. (**F**) Quantitation of cells in each phase is shown on right. For all samples, n = 3, * $p < 0.05$, ** $p < 0.01$, *** $p < 0.001$.

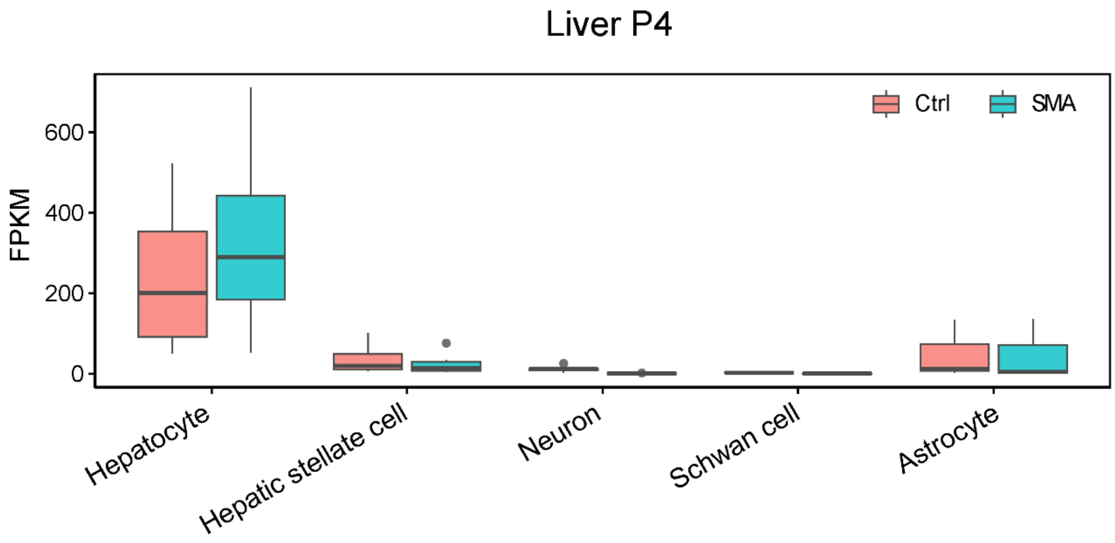

Figure S16. Expression analysis of specific gene markers for different cell types in the P4 liver RNA-seq data.

A subset of gene markers for each cell type were analyzed. The identity of the source as mouse liver was confirmed.

Figure S17

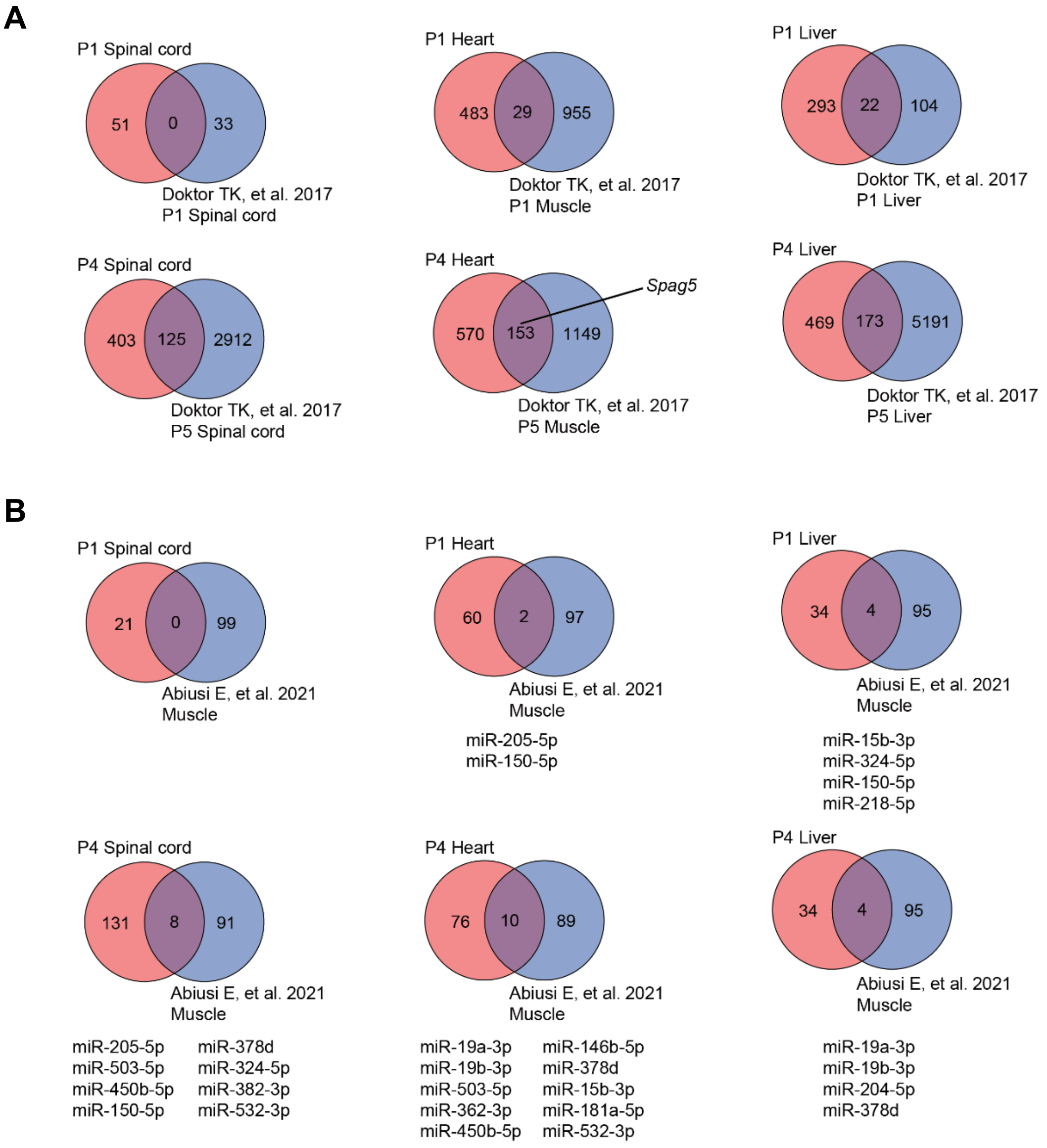

Figure S17. Comparative analysis of DEGs between the present study and two previous studies.

(**A**) Venn diagrams show shared DE-mRNAs between this study (orange, q value < 0.05 and fold-changes > 2) and the one by Doktor TK, et al. (blue, adjusted p value < 0.1) using the same mouse model. Note, Doktor TK, et al. examined spinal cord, liver and skeletal muscle but no heart, and their sampling time points were P1 and P5, so here heart was compared to skeletal muscle, and P4 to P5. (**B**) Venn diagrams show shared DE-miRNAs between this study (orange, p value < 0.05 and fold-changes > 2) and the one by Abiusi E et al. using SMA patients' muscle samples (blue, FDR < 0.05). For the two previous studies, see references #21 and #35 in the manuscript.

Table S1. List of DEGs in all ceRNETs.

Table S2. List of DEGs in miR-34a networks.

Table S3. The FPKM of specific markers for different cell types in the liver at P4.

Cell type	Marker		Ctrl	SMA
Hepatocyte	*ALB*	*Albumin*	NA	NA
	HAMP	*Hepcidin Antimicrobial Peptide*	522.704	710.887
	ARG1	*Arginase 1*	105.227	227.348
	PCK1	*Phosphoenolpyruvate Carboxykinase 1*	296.995	352.808
	AFP	*Alpha Fetoprotein*	NA	NA
	BCHE	*Butyrylcholinesterase*	49.9527	52.6371
Hepatic stellate cell	*ACTA2*	*Actin Alpha 2*	8.90057	5.48844
	COL1A1	*Collagen Type I Alpha 1 Chain*	13.3493	9.04922
	TAGLN	*Transgelin*	6.5482	3.66155
	COL1A2	*Collagen Type I Alpha 2 Chain*	25.3216	17.7321
	COL3A1	*Collagen Type III Alpha 1 Chain*	56.8307	33.3901
	SPARC	*Secreted Protein Acidic And Cysteine Rich*	101.796	75.755
Neuron	*RBFOX3*	*RNA Binding Fox-1 Homolog 3*	2.17763	0.135221
	TAU	*Microtubule Associated Protein Tau*	NA	NA
	NEFL	*Neurofilament Light Chain*	12.5567	0.093592
	MAPT	*Microtubule Associated Protein Tau*	12.6414	0.142636
	MAP2	*Microtubule Associated Protein 2*	25.1072	1.53473
	ENO2	*Enolase 2*	8.36235	0.517755
Schwan cell	*SOX10*	*SRY-Box Transcription Factor 10*	5.97526	0.245251
	S100B	*S100 Calcium Binding Protein B*	0.870555	0.038472
Astrocyte	*S100B*	*S100 Calcium Binding Protein B*	1.84123	0.076897
	GFAP	*Glial Fibrillary Acidic Protein*	1.84123	0.076897
	NDRG2	*NDRG Family Member 2*	10.9429	4.24871

NA, not available.

Table S4. Information of primers, siRNAs, and oligonucleotides used in the present study.

Primers for miRNAs expression and validation	
Name	Sequence (5'-3')
miR-34a-5p (RT)	CCTGTTGTCTCCAGCCACAAAAGAGCACAATATTTCAGGAGACAAC AGGACAACCA
miR-34a-5p (forward primer)	CGGGCTGGCAGTGTCTTAGC
miRNA (reverse primer)	CAGCCACAAAAGAGCACAAT
RNU6 (forward)	CTCGCTTCGGCAGCACATATACT
RNU6 (reverse)	ACGCTTCACGAATTTGCGTGTC
RNA probe information	
NC	Cy3-5´-UGCUUUGCACGGUAACGCCUGUUUU-3´
18S	Cy3-5´-CUUCCUUGGAUGUGGTAGCCGUUUC-3´
miR-34a-5p	Cy3-5´-ACAACCAGCUAAGACACUGCCA-3´

Table S4, continued.

Primers for lncRNAs expression and validation		
Name	Forward sequence (5'-3')	Reverse sequence (5'-3')
lnc00007921	TTGGTCGGTTGGTTGGTAA	GGCTGTGGGGAATGAGATG
lnc00138536	CCCTCAGTGTTTTGATGCC	AGTGTATGTGGGAGTTGGAAGT
lnc00150507	GCCTCTAATTTTGTTCACTGCC	TTCCAAGAATGAAAACCTCTAACC
Primers for circRNAs expression and validation		
Name	Left sequence (5'-3')	Right sequence (5'-3')
circ007386	TCTGAGCTGGCATTCAAGGA	CCCAATGCCAGCAAAGTCTT
circ014460	AAGCCACTTCCTTTGTTCCT	TTGTGACGCGACTGGAGTAT
circ007386seq	GGCGCTGGTACTTTGAATTT	GTGAGCATTTTCAGCCAACT
Primers for mRNAs expression		
Name	Forward sequence (5'-3')	Reverse sequence (5'-3')
Gapdh	CCGTAGACAAAATGGTGAAGGT	CGTGAGTGGAGTCATACTGGAA
Cdca8	ATGGCTCCCAAGAAACGC	GGTCTGTCTGTCGGACTCAAT
Cenpe	TAAAGTCCCGACAAGCATAAC	CTCCACTCTACCTCAGCCAAT
Hjurp	CCTTCCGTGACCTCATCTGTC	GCTGCTTACGCTGTTGCTG
Spag5	CTGAAGTTGGAAAATAGTCGCC	GCTCCTTGTTGCTCTGGGTA
GAPDH	AAGGTGAAGGTCGGAGTCAACG G	CCACTTGATTTTGGAGGGATCTC
SMN1/2	AGCTGTGGCTTCATTTAAGCAT	CAGAACATTTGTCCCCAACTTT

Table S4, continued.

Primers for construction of dual-fluorescence report plasmid	
Name	Sequence (5'-3')
lnc38536Wt (XhoI) F	GCGGCTCGAGTCACCTCTCAGGTCACTTGCC
lnc38536Wt (notI) R	AATGCGGCCGCGTATTTGCTTCCTCCTAAGTCAGTG
lnc8536Mut F	ACCTGAGTGCCAAAACTTGGTGACGGTAGGGCCCAGGAAAGTGACACAGATG
lnc38536Mut R	CAAGTTTTGGCACTCAGGTGACAAGAGGGGAGTCTT
circ7386-182Wt (ASI) F	GCGGGCGATCGCGTTAGAGATGACAACAAGAGACAG
circ7386-649Wt(notI) R	AATGCGGCCGCCATCACCAACATCAAAGTCCTT
circ7386-182Mut F	TGAAGAAAATGAAACTGCGATTCTATTACCAGCTGACCAG
circ7386-182Mut R	GCAGTTTCATTTTCTTCACCTTCTCTTCAGCATG
circ7386-649Mut F	TGGAGCAGGCCAGGCTGCGTTGGTCATCTAGAACTGGGTTC
circ7386-649Mut R	GCAGCCTGGCCTGCTCCAGCCCACCCGCATGTCT
*Spag5-1424*Wt (ASI) F	GCGGGCGATCGCATCTGGCTACCTTGTCCCG
*Spag5-1424*Wt (NOTI) R	AATGCGGCCGCTGTTCCAGTTGGCTGATGC
*Spag5-1424*Mut F	GCTCTCCTTGTGGGGTCAGATTCTGTGCTAAACATCTTCAGGA
*Spag5-1424*Mut R	TGACCCCACAAGGAGAGCTGTCAGTCTGTGTACT
*Spag5-3285*Wt (AsiSI) F	GCGGGCGATCGCTGCAGAGGGAAATCTGTGAAC
*Spag5-3285*Wt (NOTI) R	AATGCGGCCGCTCCAGGATGTGCCTATGGC
*Spag5-3285*Mut F	AGGCCAGCTAGATCCCAGGACGGTGCTGATGGCTACTAAC
*Spag5-3285*Mut R	CTGGGATCTAGCTGGCCTTCCAGGGCTTCCTGGA
*Spag5-3675*Wt (XhoI) F	GCGGCTCGAGAACAGCTGATGGACAAGTATCTGAG
*Spag5-3675*Wt (notI) R	AATGCGGCCGCAATAACATAAAACATGGTCGGCTC
*Spag5-3675*Mut F	ATTTGGGAACCTAACACAGTGTTGAGTCGAAAAACCCTGAAAAAT
*Spag5-3675*Mut R	CTGTGTTAGGTTCCCAAATTCCTGGGATTTGGCAGC

Table S4, continued.

Primers for construction of overexpression plasmid	
Name	Sequence (5'-3')
circ007386OE F	CGGAATTCTGAAATATGCTATCTTACAGGTTAGAGATGACAACAAGAGACAG
circ007386OE R	CGGGATCCTCAAGAAAAAATATATTCACCATCACCAACATCAAAGTCCTT
lnc00138536OEkpn1 F	ACGGTACCTCACCTCTCAGGTCACTTGCC
lnc00138536OEBamHI R	ATGGATCCGTATTTGCTTCCTCCTAAGTCAGTG
Spag5 3’UTRxbaI F	CGGTCTAGAAACAGCTGATGGACAAGTATCTGAG
Spag5 3’UTRBamHI R	AATGGATCCAATAACATAAAACATGGTCGGCTC
*Spag5*cdsxbaI F	ACTCTAGAATGTGGAGGGTGAAAACACTGAA
*Spag5*cdskpn1 R	ACGGTACC TTAGCTCAGAAATTCTAGCAATCCT

siRNAs and oligonucleotides		
Name	Sense sequence (5'-3')	Antisense sequence (5'-3')
Negative control (NC)	UUCUCCGAACGUGUCACGUTT	ACGUGACACGUUCGGAGAATT
si*Smn*	GACCUGUGAAGUAGCUAAUTT	AUUAGCUACUUCACAGGUCTT
si*SMN*	CUUGAUGAUGCUGAUGCUUU	AAAGCAUCAGCAUCAUCAAG
miR-34a-5p mimic	UGGCAGUGUCUUAGCUGGUUGU	AACCAGCUAAGACACUGCCAUU
miR-34a-5p inhibitor	ACAACCAGCUAAGACACUGCCA	

Table S4, continued.

Sequences of decoy MRE oligonucleotides for target genes with modified phosphorothioate backbone	
Spag5 3’UTR WT	ACAACACAGCAAAAACCCUGAAA
Spag5 3’UTR Mut	UGUUGAGUCGAAAAACCCUGAAA
lnc00138536 WT	AGUGCCAAAACUUGCACUGCCA
lnc00138536 Mut	AGUGCCAAAACUUGGUGACGGU
circ007386 WT1	GAAACUGCCUAAGAAUUACCA
circ007386 WT2	CCAACCAGAUCUAGAACUGGGU
circ007386 Mut1	GAAACUGCGAUUCUAUUACCA
circ007386 Mut2	CGUUGGUCAUCUAGAACUGGGU
